# Unveiling novel serum biomarkers in intrahepatic cholangiocarcinoma: a pilot proteomic exploration

**DOI:** 10.3389/fphar.2024.1440985

**Published:** 2024-09-02

**Authors:** Lavinia Patricia Mocan, Cristiana Grapa, Rareș Crăciun, Ioana Ecaterina Pralea, Alina Uifălean, Andreea Maria Soporan, Ximena Maria Mureșan, Maria Iacobescu, Nadim Al Hajjar, Carmen Mihaela Mihu, Zeno Spârchez, Tudor Mocan, Cristina Adela Iuga

**Affiliations:** ^1^ Department of Histology, “Iuliu Haţieganu” University of Medicine and Pharmacy, Cluj-Napoca, Romania; ^2^ Department of Gastroenterology, “Prof. Dr. Octavian Fodor” Regional Institute of Gastroenterology and Hepatology, Cluj-Napoca, Romania; ^3^ Department of Gastroenterology and Hepatology, “Iuliu Haţieganu” University of Medicine and Pharmacy, Cluj-Napoca, Romania; ^4^ Department of Proteomics and Metabolomics, Institute of Medical Research and Life Sciences – Medfuture, “Iuliu Haţieganu” University of Medicine and Pharmacy, Cluj-Napoca, Romania; ^5^ Department of Pharmaceutical Analysis, Faculty of Pharmacy, “Iuliu Haţieganu” University of Medicine and Pharmacy, Cluj-Napoca, Romania; ^6^ Department of Translational Medicine, Institute of Medical Research and Life Sciences – Medfuture, “Iuliu Haţieganu” University of Medicine and Pharmacy, Cluj-Napoca, Romania; ^7^ Department of Surgery, “Iuliu Haţieganu” University of Medicine and Pharmacy, Cluj-Napoca, Romania; ^8^ UBBMed Department, Babeş-Bolyai University, Cluj-Napoca, Romania

**Keywords:** biomarker, intrahepatic cholangiocarcinoma, hepatocellular carcinoma, liver cirrhosis, primary sclerosing cholangitis, proteomics

## Abstract

Recent advancements in proteomics have shown promise in identifying biomarkers for various cancers. Our study is the first to compare the serum proteomes of intrahepatic cholangiocarcinoma (iCCA) with cirrhosis (CIR), primary sclerosing cholangitis (PSC), and hepatocellular carcinoma (HCC), aiming to identify a proteomic signature that can effectively distinguish among these conditions. Utilizing high-throughput mass spectrometry on serum samples, we identified 845 proteins, of which 646 were suitable for further analysis. Unique clustering patterns were observed among the five groups, with significant proteomic differences. Our key findings include: S100 calcium-binding protein A9 (S100A9) and haptoglobin (HP) were more abundant in iCCA, while intercellular adhesion molecule 2 (ICAM2) was higher in HCC. Serum amyloid A1 (SAA1) and A4 (SAA4) emerged as potential biomarkers, with SAA1 significantly different in the iCCA vs healthy controls (HC) comparison, and SAA4 in the HCC vs HC comparison. Elevated levels of vascular cell adhesion molecule 1 (VCAM-1) in HCC suggested its potential as a differentiation and diagnostic marker. Angiopoietin-1 receptor (TEK) also showed discriminatory and diagnostic potential in HCC. ELISA validation corroborated mass spectrometry findings. Our study underscores the potential of proteomic profiling in distinguishing iCCA from other liver conditions and highlights the need for further validation to establish robust diagnostic biomarkers.

## 1 Introduction

Cholangiocarcinoma (CCA), originating from cholangiocytes in the biliary duct system, is the second most common primary liver cancer, following hepatocellular carcinoma (HCC). Known for its aggressive behavior among biliary tract malignancies, global projections forecast a surge in its incidence (Rumgay et al., 2022). CCA is typically categorized as either intrahepatic (iCCA) or extrahepatic and predominantly affects individuals in their seventies, with a slight male predominance (Shaib et al., 2005). Contributing risk factors include viral hepatitis infections, inflammatory conditions such as obesity and diabetes mellitus, and congenital hepatic fibrosis (Shaib Hashem and Yasser, 2004). Notably, primary sclerosing cholangitis (PSC) significantly predisposes individuals to CCA, with about 40% of PSC patients at risk. The carcinogenesis mechanisms in this context involve chronic inflammation, epithelial proliferation, bile mutagens, and biliary stasis (Claessen et al., 2009; Izquierdo-Sanchez et al., 2022).

Despite advancements in diagnostic techniques such as computed tomography (CT) and ultrasound (US), their sensitivity remains limited, particularly for detecting small liver lesions (Patel et al., 2000). Additionally, distinguishing between HCC, PSC and liver cirrhosis (CIR) poses significant challenges. Commonly used biomarkers like carcinoembryonic antigen (CEA) and carbohydrate antigen 19-9 (CA19-9) do not possess the necessary sensitivity or specificity for effective CCA detection (Shi et al., 2013). Furthermore, the diagnostic reliability of CA 19-9 and alpha-fetoprotein (AFP) is compromised due to their variable elevation in different liver diseases (Best et al., 2020; Zhou et al., 2011; Izquierdo-Sanchez et al., 2022).

The need for improved medical approaches highlights the critical importance of developing innovative strategies for the early detection and management of CCA (Mocan et al., 2022). Omics sciences offer promising pathways, as evidenced by their success in various other cancer types. However, the rarity of CCA cases has hindered our molecular understanding, resulting in limited comprehensive omics studies. While genomic and transcriptomic research has illuminated key genes and molecular subtypes associated with iCCA development (Voigtländer et al., 2020; Job et al., 2020; Martin-Serrano et al., 2023), the proteomic landscape of iCCA remains relatively unexplored. Current studies have primarily focused on specific histotypes or ethnic backgrounds, often linked to relevant risk factors. For instance, Lapitz et al. focused on CCA with an emphasis on PSC-CCA patients, employing proteome profiling of serum extracellular vesicles (EVs). This study identified diagnostic biomarkers such as C-reactive protein (CRP), Fibrinogen, and von Willebrand factor (VWF) for early diagnosis and prognosis (Lapitz et al., 2023). Christensen et al. targeted biliary tract cancer (BTC) broadly, using proximity extension assays and statistical modeling to generate multiprotein signatures, including CA19-9 and chemokine C-C motif ligand 20, for differentiating BTC from non-cancer controls (Christensen et al., 2023).

Recently, an integrated proteogenomic profile delineating iCCA subtypes was introduced, laying the groundwork for future research (Dong et al., 2022). Proteomics, as a leading technology, holds significant promise for identifying disease-specific biomarkers essential for diagnosis, prognosis, and treatment response assessment (B. B. Sun, Suhre, and Gibson, 2024). Despite extensive knowledge of CCA’s transcriptomic and mutational landscapes, its proteomic landscape remains largely uncharted.

Consequently, there is an urgent need for proteomics-driven research to discover new biomarkers, which are vital for improving our understanding and management of CCA across various populations and subtypes.

From a clinical perspective, there is a pressing need for biomarkers to differentiate between iCCA and HCC, as well as between iCCA and PSC. Imagine the ideal scenario where we could screen for HCC in cirrhotic patients, detect a nodule, and inform the patient whether it is HCC or iCCA, all based on just two drops of blood. Wouldn't it be incredible to have the capability to distinguish early between PSC and iCCA based on non-invasive biomarkers?

Hence, our study aims to explore the potential of proteomics in uncovering valuable serum biomarkers capable of distinguishing between iCCA and HCC, PSC, CIR and healthy controls (HC). By leveraging proteomic analysis, we seek to identify novel biomarkers with enhanced diagnostic accuracy and clinical relevance. Through comparative analysis of the serum proteome profiles, our research endeavors to unveil unique protein signatures associated with distinct liver pathologies, thereby facilitating early detection and the development of personalized treatment strategies.

## 2 Results

### 2.1 Baseline characteristics of the study participants

A detailed overview of the study participants baseline characteristics can be found in Supplementary Table S1. The table provides a detailed comparison of demographics, environmental factors, and laboratory values for both discovery and validation cohorts. The mean age of the patients varies across cohorts, with iCCA, HCC, CIR patients averaging around 60 years, PSC patients around 50 years, and HC at around 30 years. Gender distribution shows a predominance of males in the iCCA and CIR cohorts and a higher proportion of females in the PSC cohort. Environmental exposure indicates that most iCCA and PSC patients are from urban areas, while the HCC cohort shows a more balanced distribution. Laboratory findings highlight variations in albumin levels, AFP, CA 19-9, and other liver function tests across cohorts, reflecting the different disease states. This comprehensive data underscores the clinical profiles and underlying health conditions of the patients and is in line with the characteristics of the liver diseases.

### 2.2 Serum proteome characterization

845 proteins with at least one unique peptide were identified. After applying the criterion for inclusion in the analysis (please see Section 4.3.3 Statistical Analysis), a total number of 646 proteins were further subjected to the biomarker analysis. The complete list of the proteins considered for the analysis is shown in Supplementary Table S2. A complete overview of the statistical analysis results is shown in Supplementary Table S3 where only the significant proteins among all comparisons are shown.

### 2.3 Serum proteome pattern exploration

**FIGURE 1:**
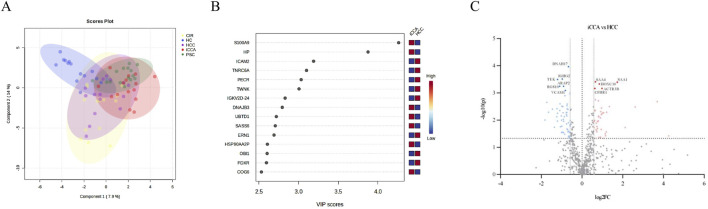
**(A)**. Sampling group clustering by the serum proteome of the groups studied (CIR = liver cirrhosis; HC = healthy controls; HCC = hepatocellular carcinoma; iCCA = intrahepatic cholangiocarcinoma; PSC = primary sclerosing cholangitis). **(B)**. Top discriminatory serum proteins among the iCCA and HCC patient’s groups. **(C)**. Volcano plot showing all significantly different serum proteins (*p* < 0.05, |FC| >1.5, proteins with FDR<0.05 are labeled) among the iCCA and the HCC patients’ groups.

As an initial demonstration of the study’s viability, we conducted a group clustering analysis. The serum patterns identified exhibited a noteworthy clustering pattern among the studied groups as shown through the utilization of partial least squares discriminant analysis (PLS-DA), demonstrating discernible separation according to the first two components (Figure 1A).

### 2.4 Serum proteome alterations in liver cancer

By elucidating the discriminatory protein profile through PLS-DA (Figure 1A) and visualizing them in the variable importance in projection (VIP) score plot, top discriminatory proteins among the iCCA and the HCC patient groups were highlighted. The most significant three proteins were: S100 calcium-binding protein A9 (S100A9), Haptoglobin (HP), and Intercellular Adhesion Molecule 2 (ICAM2) (Figure 1B). For scouting potential biomarkers, t-test was applied. A total of 154 proteins exhibited significant differences (*p* < 0.05, no false discovery rate (FDR) cut-off) in the comparison between iCCA and HCC, out of which 65 had also a minimum of 1.5-fold change (FC) (log2FC > 0.58). Also considering the FDR criterion of <0.05, 11 proteins were highlighted and the findings are shown in a volcano-plot (Figure 1C). For the 11 proteins, a receiver operating characteristic analysis (ROC) was applied and the area under the ROC curve (AUC) was calculated showing values greater than 0.80. Table 1 presents a comprehensive overview which includes the iCCA vs. HCC analysis: the 11 statistically significant proteins, the FC, AUC, sensitivity (SEN), specificity (SPE). To gain a more in-depth understanding, we additionally assessed the significance of the proteins between the iCCA and the HC serum samples, as well as between the HCC and the HC serum samples, and provided only the FC for the significant proteins (Table 1).

**TABLE 1:** Serum proteins that could aid iCCA vs. HCC discrimination.

			iCCA vs. HCC				iCCA vs. HC	HCC vs. HC
No.	Protein name	Gene	FC	AUC	SEN	SPE	FC	FC
1	Serum amyloid A-1 protein*	SAA1	3.3	0.86	0.7	0.9	3.2	ns
2	Actin-related protein 3B*	ACTR3B	2.0	0.88	0.8	0.9	2.6	ns
3	Homeobox protein Hox-C10*	HOXC10	1.8	0.87	0.8	0.8	2.3	ns
4	Serum amyloid A-4 protein	SAA4	1.6	0.87	0.7	0.9	ns	−1.5
5	Complement factor H-related protein 1	CFHR1	1.5	0.86	0.8	0.7	ns	−1.7
6	Dynein axonemal heavy chain 17*	DNAH17	−1.6	0.87	0.7	0.8	−1.3	ns
7	Vascular cell adhesion protein 1**	VCAM1	−1.8	0.87	0.8	0.8	2.36	4.2
8	Arf-GAP with Rho-GAP domain ANK repeat and PH domain-containing protein 2*	ARAP2	−1.9	0.85	0.9	0.7	ns	2.3
9	Immunoglobulin heavy constant gamma 2**	IGHG2	−2.0	0.89	0.8	0.8	1.9	3.7
10	Regulator of G-protein signaling 10**	RGS10	−2.1	0.84	0.7	0.9	1.9	4.0
11	Angiopoietin-1 receptor**	TEK	−2.3	0.90	0.9	0.9	2.2	5.0

iCCA, intrahepatic cholangiocarcinoma; HCC, hepatocellular carcinoma; HC, healthy controls; FC , fold change (calculated as the ratio of the two group means, AUC, area under the receiver operating characteristic (ROC) curve; SEN, sensitivity; SPE, specificity; ns = no significance for t-test, therefore also no FC, reported; *significant for iCCA, vs. HCC, and either iCCA, vs. HC, or HCC, vs. HC; **significant for all three comparisons.

### 2.5 Serum proteome alterations towards anticipating the upcoming malignancy in the risk factor groups

One-way analysis of variance (ANOVA) identified 47 proteins with statistically significant differences (*p* < 0.05) in abundance across the groups. This analysis considered potential iCCA risk factors like PSC and CIR. Additionally, these proteins exhibited a fold change (FC) greater than 1.5, further supporting their statistical significance and potential relevance to iCCA. Furthermore, to delve deeper into the analysis, we evaluated the significance of proteins between iCCA, CIR, PSC and HC.

For the iCCA vs. CIR comparison, 28 proteins emerged as potential discriminators. Table 2 provides a detailed breakdown, including FC values and ROC analysis parameters specific to the iCCA vs CIR comparison. Additionally, the table presents FC values for proteins showing significant differences when comparing both iCCA and CIR to HC.

**TABLE 2:** Serum proteome alterations in the iCCA group compared to the risk factor group CIR.

No.	Protein name	Gene	FC	AUC	SEN	SPE	iCCA vs. HC	CIR vs. HC
1	Haptoglobin*	HP	3.2	0.73	0.9	0.6	12.9	ns
2	cAMP-dependent protein kinase catalytic subunit beta*	PRKACB	2.5	0.79	0.7	0.7	ns	−2.7
3	Heat shock protein HSP 90-alpha A2	HSP90AA2P	2.3	0.78	0.8	0.8	ns	ns
4	Chromatin assembly factor 1 subunit A*	CHAF1A	2.0	0.79	0.8	0.7	3.0	ns
5	Trinucleotide repeat-containing gene 18 protein	TNRC18	2.0	0.79	0.7	0.7	ns	ns
6	Protein CIP2A*	CIP2A	1.5	0.73	0.7	0.8	ns	−1.7
7	Voltage-dependent calcium channel subunit alpha-2/delta-3	CACNA2D3	1.5	0.83	0.9	0.7	ns	−1.4
8	M-phase inducer phosphatase 1*	CDC25A	−1.5	0.79	0.8	0.7	−2.0	ns
9	Unconventional myosin-XV*	MYO15A	−1.5	0.78	0.9	0.7	−1.8	ns
10	Zygote arrest protein 1**	ZAR1	−1.5	0.76	0.7	0.7	−2.5	−1.6
11	Huntingtin-interacting protein 1*	HIP1	−1.5	0.84	0.7	0.9	−1.9	ns
12	Villin-like protein*	VILL	−1.5	0.80	0.7	0.8	−1.9	ns
13	Transcription factor ETV7*	ETV7	−1.6	0.77	0.7	0.7	−2.2	ns
14	ELKS/Rab6-interacting/CAST family member 1*	ERC1	−1.7	0.80	0.7	0.7	−2.3	ns
15	Proline/serine-rich coiled-coil protein 1*	PSRC1	−1.7	0.85	0.7	0.9	−1.9	ns
16	Neurofibromin*	NF1	−1.7	0.72	0.7	0.6	ns	1.8
17	Prefoldin subunit 1*	PFDN1	−1.8	0.83	0.8	0.8	ns	2.2
18	Regulator of G-protein signaling 10**	RGS10	−1.9	0.83	0.7	0.8	1.9	3.6
19	Microtubule-associated protein 1A*	MAP1A	−1.9	0.83	0.8	0.8	ns	2.0
20	Annexin A9*	ANXA9	−1.9	0.90	0.9	0.9	−2.1	ns
21	Complexin-4*	CPLX4	−2.1	0.91	0.8	0.9	−1.9	ns
22	Caveolin-3**	CAV3	−2.1	0.74	0.5	0.9	2.1	4.5
23	Adaptin ear-binding coat-associated protein 2*	NECAP2	−2.2	0.80	0.9	0.7	ns	5.6
24	Calcium-binding mitochondrial carrier protein SCaMC-2*	SLC25A25	−2.2	0.80	0.7	0.8	ns	3.0
25	Paired box protein Pax-9*	PAX9	−2.2	0.79	0.6	0.9	ns	2.8
26	Peroxisomal trans-2-enoyl-CoA reductase*	PECR	−2.3	0.78	0.7	0.7	−2.6	ns
27	Alkaline phosphatase_ tissue-nonspecific isozyme*	ALPL	−3.1	0.83	0.7	1.0	ns	4.2
28	ORC ubiquitin ligase 1*	OBI1	−4.6	0.86	0.9	0.8	ns	3.4

iCCA, intrahepatic cholangiocarcinoma; CIR, liver cirrhosis; HC, healthy controls; FC , fold change (calculated as the ratio of the two group means, AUC, area under the receiver operating characteristic (ROC) curve; SEN, sensitivity; SPE, specificity; ns = no significance for t-test, therefore also no FC is reported; *significant for both iCCA, vs. PSC, and iCCA, vs. HC.

For the iCCA vs. PSC comparison, Table 3 presents a comprehensive overview on the data, which includes 27 statistically significant proteins, the FC, AUC, SEN, SPE. We then reported the FC only for those proteins with significant differences between iCCA and HC patients. No proteins met the same stringent criteria for showing significance between PSC and HC patients.

**TABLE 3:** Serum proteome alterations in the iCCA group compared to the risk factor group PSC.

			iCCA vs. PSC				iCCA vs. HC
No.	Protein name	Gene	FC	AUC	SEN	SPE	FC
1	Aminopeptidase Q*	LVRN	14.0	0.83	0.90	0.70	15.7
2	Leucine-rich alpha-2-glycoprotein*	LRG1	1.9	0.77	0.60	0.90	2.2
3	Melanophilin	MLPH	1.9	0.82	0.90	0.90	ns
4	Probable asparagine--tRNA ligase_ mitochondrial	NARS2	1.9	0.68	0.80	0.50	ns
5	Alpha-1-antichymotrypsin	SERPINA3	1.8	0.80	0.80	0.80	ns
6	Immunoglobulin lambda constant 7*	IGLC7	1.7	0.80	0.80	0.80	1.9
7	NACHT_ LRR and PYD domains-containing protein 14*	NLRP14	1.6	0.83	0.70	0.80	1.6
8	Huntingtin-interacting protein 1*	HIP1	−1.5	0.87	0.90	0.80	−1.9
9	Nicotinamide N-methyltransferase*	NNMT	−1.5	0.76	0.70	0.80	−2.2
10	Annexin A9*	ANXA9	−1.5	0.78	0.90	0.60	−2.1
11	Phosphatidylcholine-sterol acyltransferase*	LCAT	−1.5	0.76	0.80	0.70	−1.9
12	Unconventional myosin-XV*	MYO15A	−1.5	0.74	0.70	0.80	−1.8
13	M-phase inducer phosphatase 1*	CDC25A	−1.5	0.76	0.70	0.80	−2.0
14	EF-hand calcium-binding domain-containing protein 3*	EFCAB3	−1.5	0.75	0.90	0.50	−2.0
15	Villin-like protein*	VILL	−1.6	0.76	0.70	0.80	−1.9
16	ELKS/Rab6-interacting/CAST family member 1*	ERC1	−1.6	0.79	0.70	0.70	−2.3
17	Transcription factor ETV7*	ETV7	−1.7	0.77	0.70	0.80	−2.2
18	Sialic acid-binding Ig-like lectin 9*	SIGLEC9	−1.7	0.74	0.70	0.80	−2.3
19	Probable rRNA-processing protein EBP2*	EBNA1BP2	−1.7	0.77	0.60	0.90	−1.6
20	Complexin-4*	CPLX4	−1.7	0.80	0.80	0.80	−1.9
21	Glucose-6-phosphate 1-dehydrogenase	G6PD	−1.9	0.80	0.90	0.70	ns
22	Insulin-like growth factor-binding protein 3*	IGFBP3	−1.9	0.80	0.70	0.90	−3.0
23	Cilia- and flagella-associated protein 36*	CFAP36	−2.1	0.84	0.70	0.90	−3.0
24	Serine/threonine-protein kinase MRCK beta	CDC42BPB	−2.1	0.85	0.90	0.70	ns
25	Zinc finger protein 532*	ZNF532	−2.2	0.80	0.80	0.80	−4.1
26	Melanoma-associated antigen 2*	MAGEA2	−2.5	0.83	0.90	0.70	−2.3
27	DNA repair protein XRCC2*	XRCC2	−4.2	0.76	0.80	0.70	−10.4

iCCA, intrahepatic cholangiocarcinoma; PSC, primary sclerosing cholangitis; HC, healthy controls; FC , fold change (calculated as the ratio of the two group means, AUC, area under the receiver operating characteristic (ROC) curve; SEN, sensitivity; SPE, specificity; ns = no significance for t-test, therefore also no FC is reported; *significant for both iCCA, vs. PSC, and iCCA, vs. HC.

### 2.6 Biomarker panel scounting

**FIGURE 2:**
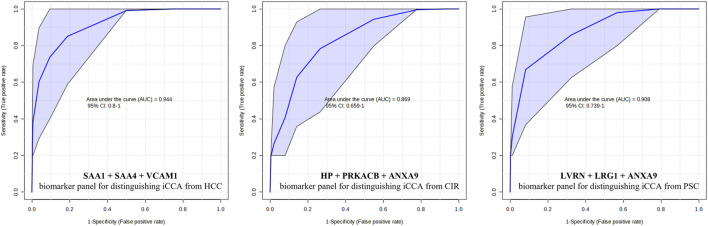
Proposed biomarker panels for distinguishing the different liver diseases. AUC = area under the receiver operating characteristic (ROC) curve; CI = confidence interval; iCCA = intrahepatic cholangiocarcinoma; HCC = hepatocellular carcinoma; CIR = liver cirrhosis; PSC = primary sclerosing cholangitis; SAA1 = Serum amyloid-1; SAA4 = Serum amyloid-4; VCAM1 = Vascular cell adhesion protein 1; LRG1 = HP = Haptoglobin, PRKACB = cAMP-dependent protein kinase catalytic subunit beta; ANXA9 = Annexin A9; LVRN = Leucine-rich alpha-2-glycoprotein.

For each of the targeted comparisons, proteins from the significant ones were tested by ROC analysis using the PLS-DA algorithm for inclusion into biomarker panels. The selection was based on their significance level both between the liver diseases groups and the HC, as well as their implication in the liver disease, as reported in the literature. The most promising panels showing an AUC 0.9 are presented in Figure 2.

### 2.7 Quantitation of serum biomarkers SAA1, SAA4, VCAM1, and LRG1 by ELISA

**FIGURE 3:**
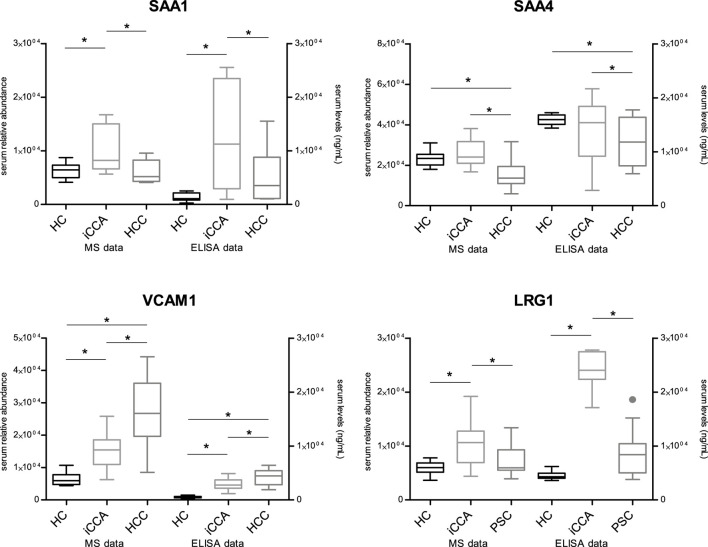
Validation of mass spectrometry-based results by ELISA HC = healthy controls; iCCA = intrahepatic cholangiocarcinoma; HCC = hepatocellular carcinoma; PSC = primary sclerosing cholangitis; SAA1 = Serum amyloid-1; SAA4 = Serum amyloid-4; VCAM1 = Vascular cell adhesion protein 1; LRG1 = Leucine-rich alpha-2-glycoprotein; * = statistically significant (*p* < 0.05); MS = mass spectrometry; ELISA = enzyme-linked immunosorbent assay.

Four proteins were selected to be analyzed on an independent patient validation cohort by a complementary method, namely, an enzyme-linked immunosorbent assay (ELISA). SAA1, SAA4, VCAM1, and LRG1 were chosen for quantification from the identified proteins in undepleted serum samples from the iCCA, HCC, and PSC groups. Selection criteria for these proteins included their abundance patterns across the groups, potential links to liver pathophysiology, and the significance of their differences between the groups. The serum levels of these markers are presented in Table 4 as mean (ng/mL) ± standard deviation (SD). The significance among the groups was tested by t-test and SAA1, SAA4, VCAM1, and LRG1 showed the same significance as the MS data. Figure 3 shows the results.

**TABLE 4:** Serum levels of SAA1, SAA4, VCAM1, and LRG1 by ELISA.

ng/mL ± SD	HC	iCCA	HCC	PSC
SAA1	1,351.08 ± 759.56	13417.21 ± 10106.14	5,766.75 ± 4667.69	
SAA4	42550.59 ± 2501.47	37907.35 ± 14592.45	31350.66 ± 11565.65	
VCAM1	879.79 ± 299.49	4901.46 ± 1815.49	7099.19 ± 2540.60	
LRG1	4510.32 ± 716.18	23974.41 ± 3411.92		10205.67 ± 6838.53

SD, standard deviation; HC, healthy controls; iCCA, intrahepatic cholangiocarcinoma; HCC, hepatocellular carcinoma; PSC, primary sclerosing cholangitis; SAA1 = Serum amyloid-1; SAA4 = Serum amyloid-4; VCAM1 = Vascular cell adhesion protein 1; LRG1 = Leucine-rich alpha-2-glycoprotein.

## 3 Discussion

Recently, there has been growing interest and investment in proteomics to identify biomarkers for various diseases, including different cancers. Our study is the first to compare the serum proteomes of iCCA with those of CIR, PSC, and HCC, aiming to identify a proteomic signature that can effectively distinguish among them. The findings highlight the urgent need for a proteomics-driven research to discover new biomarkers essential for improving our understanding and management of iCCA, especially given the largely unexplored proteomic landscape of this malignancy. Differentiating between iCCA and HCC is crucial in clinical practice, but it remains challenging, often requiring a liver biopsy for a definitive diagnosis (Mocan et al., 2023). Liver biopsy is an invasive procedure with potential risks such as bleeding and tumor seeding. Currently, there are no biomarkers available to differentiate between iCCA and HCC, or between iCCA and CIR or PSC, known as risk factors.

Our findings stem from a thorough proteomic approach, utilizing high-throughput mass spectrometry techniques on serum samples depleted of six highly abundant proteins through immunoaffinity. Following our methodology, we successfully identified 845 proteins that met the identification criteria outlined in Section 4.4. Of these, 646 proteins were considered suitable for subsequent analysis, consistent with similar studies (Lucaciu et al., 2023). Notably, our approach was the first to include the four patient groups alongside healthy controls. As proof of concept, we successfully identified unique clustering patterns among the five groups using PLS-DA analysis (Figure 1A). Interestingly, the serum proteome clusters of the four liver diseases were noticeably different compared to HC, with partial overlap among them, highlighting a shared protein profile corresponding to similarities in liver disease. Particularly, the clustering clearly illustrated the relationship between iCCA and HCC, as well as the associations with their risk factor groups. The initial investigation into serum proteome alterations commenced with a wide-ranging comparative analysis between the iCCA and HCC groups. Discriminatory proteins were identified initially using the VIP score plot generated from the PLS-DA. The top three discriminatory proteins between the iCCA and HCC groups were S100 calcium-binding protein A9 (S100A9) and haptoglobin (HP), which were more abundant in iCCA, and Intercellular adhesion molecule 2 (ICAM2), which was higher in HCC (Figure 1B).

Additionally, we employed supplementary statistical analyses, along with stringent criteria, to further confirm the identification of potential biomarkers. Consequently, our comparison revealed distinct protein profiles, with five serum proteins exhibiting increased abundance in the iCCA group and six proteins in the HCC group. Additionally, their significance for the comparison to HC was also interrogated. Remarkably, the ROC analysis for both individual markers demonstrated robust discriminatory power as shown in Table 2. Among the proteins showing increased abundance in iCCA compared to HCC, serum amyloid A1 and A4 were identified. Even more, SAA1 was found to be significantly different in the iCCA vs. HC comparison, and SAA4 in the HCC vs. HC comparison. Serum amyloid A (SAA) proteins, consisting of SAA one to 4, are small molecules (104 amino acids) intricately linked to the acute phase response. Although their precise physiological roles remain elusive (Sack, 2018), recent studies suggest their potential as biomarkers for various cancers, including gastric, colon, and breast cancer, and their correlation with tumor staging (Ignacio et al., 2019; Liang et al., 2016; Wu et al., 2017). A decreased abundance of SAA1 in HCC, correlated with reduced survival rates and involvement in anti-tumor immune pathways, was observed (Zhang Hongying et al., 2020). Moreover, through scRNA-seq analysis, a correlation between SSA1 expression and the regulation of inflammatory responses and activation of the complement system, indicating a potential role in iCCA pathogenesis, was highlighted (Zhang Min et al., 2020). Thus, our study supports the utility of SAA proteins as biomarkers for distinguishing between iCCA and HCC. In light of these findings, SAA1 and SAA4 emerged as candidates for further validation.

Elevated levels of Vascular cell adhesion molecule 1 (VCAM-1) were observed in the HCC group, indicating its potential as a differentiation marker. Furthermore, significant differences in VCAM-1 levels were also noted in comparisons between both iCCA and HCC vs. HC, highlighting its utility as a discriminatory marker between iCCA and HCC, as well as a diagnostic marker for both iCCA and HCC. Based on these findings, VCAM-1 was also selected for further validation. VCAM-1 plays a crucial role in angiogenesis, leukocyte adhesion at inflamed tissues, and tumor sites, indicating its involvement in facilitating metastasis (Fox et al., 1995; Ho et al., 2004). Consistently elevated expression of VCAM-1 in chronic liver diseases imply its association with conditions such as chronic hepatitis or CIR (Kaplanski et al., 1997; Yamaguchi et al., 1999). However, despite this, the clinical significance of increased serum VCAM-1 in HCC patients remains largely unexplored, even though the majority of HCC cases are associated with underlying chronic hepatitis or CIR. Therefore, further investigation into serum VCAM-1 levels in HCC patients is warranted (Pirisi et al., 1996). Another protein exhibiting increased abundance in the HCC group was angiopoietin-1 receptor (TEK). Like VCAM1, TEK also showed both discriminatory and diagnostic potential. Angiopoietin-1, among factors implicated in tumor angiogenesis, has been linked to HCC progression and has shown superior performance to AFP in predicting overall survival (Choi et al., 2021). Given the pivotal role of angiogenesis inhibitors in the treatment of solid cancers, investigating angiogenic cytokines such as angiopoietin-1 can yield valuable insights into the progression of HCC (Torimura et al., 2004). Our findings underscore the promising clinical significance of these proteins, reaffirming their potential utility as biomarkers for distinguishing between iCCA and HCC. Moreover, a biomarker panel was built and tested by ROC analysis using the PLS-DA algorithm. This was comprised of SAA1, SAA4 and VCAM1 and showed a promising discrimination power.

In clinical practice iCCA is often diagnosed on a background of advanced liver disease, such as CIR, or in patients with a known history of PSC. These diagnoses invariably depend on invasive procedures, such as liver biopsy. Due to the absence of reliable biomarkers, iCCA is often detected at an advanced stage, at which point curative treatment options are no longer viable. Our study is the first one to reveal 64 proteins that displayed notable differences among the iCCA, CIR and PSC groups.

In the iCCA vs. CIR comparison, seven proteins showed higher abundance in the iCCA patient group, among which haptoglobin (HP), cAMP-dependent protein kinase catalytic subunit beta (PRKACB), heat shock protein (HSP) 90-alpha A2 (HSP90AA2P) were previously reported with respect to liver cancer. Haptoglobin (HP), a plasma glycoprotein, scavenges free hemoglobin, preventing release of toxic heme and protecting tissues from oxidative damage. Its dynamic response in pathology makes HP a valuable clinical tool. Moreover, HP was shown to be elevated in liver cancer and decreased in cirrhosis (Naryzny and Legina, 2021). Our results revealed also a significant difference of HP in the iCCA vs. HC groups, confirming the above mentioned findings.

PRKACB, a vital member of the protein kinase A (PKA) family, regulates various cellular processes through the phosphorylation of target proteins. Dysregulated PKA signaling has been implicated in the progression of liver cancer, affecting tumor growth, invasion, and metastasis, as well as hepatic fibrosis in CIR (Zhang Wei et al., 2020). However, direct studies linking PRKACB to iCCA and CIR are limited. HSPs act as molecular chaperones essential for protein folding, stabilization, and degradation, thus maintaining cellular homeostasis and responding to stress. Specifically, HSP90AA2P, a member of the HSP90 family, modulates various cellular processes, including cell proliferation, survival, and apoptosis. In liver cancer, upregulated HSP90AA2P promotes cancer cell survival, proliferation, and stabilizes oncoproteins and signaling pathways associated with tumor progression. In the context of CIR, HSP90AA2P likely contributes to the cellular stress response induced by chronic liver injury and inflammation, potentially affecting fibrosis progression (Whitesell and Lindquist, 2005). Notably, HSP90 was recently identified as a prognostic marker for CCA using an immunohistochemistry approach (Shirota et al., 2015).

Among the proteins exhibiting significantly higher abundance in the CIR group compared to iCCA, Annexin A9 (ANXA9) stood out, being also significantly different in the iCCA vs. HC comparison.

Annexins, a superfamily of calcium-dependent phospholipid-binding proteins, play various roles in biological processes. Despite comprising 13 human members and frequently being dysregulated in cancer, their expression patterns and prognostic values in liver cancer remain largely unexplored. The potential of ANXAs 1-5 and 10 as therapeutic targets was described, while ANXAs 2, 5, 7, and 10 could serve as prognostic markers in liver cancer (Zhuang et al., 2019). Additionally, ANXAs 10 and 13 have shown promise as differential diagnostic biomarkers between iCCA and pancreatic cancer (Geramizadeh et al., 2021; Serag and Elsayed, 2021).

In the comparison between iCCA and PSC, some of the proteins displaying higher serum abundance in the iCCA group have been previously reported, such as alpha-1-antichymotrypsin (SERPINA3), Chromatin assembly factor 1 subunit A (CHAF1A), and Leucine-rich alpha-2-glycoprotein (LRG1). SERPINA3, predominantly secreted by the liver, has been found to have a decreased abundance in HCC, a phenomenon correlated with enhanced cell proliferation (Santamaria et al., 2013; Sun et al., 2023; Soman and Nair, 2022) with elevated expression serving as a diagnostic and poor prognosis biomarker, indicating resistance to endocrine therapy and chemotherapy, and potentially predicting immunotherapy sensitivity. LRG1, an inflammatory protein, is known to play a critical role in tumorigenesis, development, and metastasis in various tumors (Lin et al., 2022; Zou et al., 2022). Its elevated abundance was reported to serve as an independent prognostic factor in patients with postoperative iCCA (Jin et al., 2020). In our study, LRG1 showed discriminatory power between iCCA and PSC and a diagnostic potential for iCCA, because of the significant difference observed in the iCCA vs. HC comparison. Having a dual role, LRG1 was selected for further validation by ELISA.

To delve deeper into our approach towards biomarker discovery for liver diseases, particularly in discriminating iCCA from HCC, and the associated risk factors CIR and PSC, we focused on identifying biomarker panels comprising combinations of the most relevant proteins. Biomarker panels are more powerful than single biomarkers because they provide a comprehensive view of the biological processes underlying a condition. A single biomarker may offer limited insight and be influenced by various factors, whereas a panel combines multiple biomarkers, enhancing diagnostic accuracy and reliability. This comprehensive approach improves the detection of complex diseases, tracks progression, and tailors personalized treatments by capturing a broader spectrum of biological activities and interactions. Such an approach was done by Kim et al. (Kim et al., 2022) for HCC. Consequently, we proposed three panels for the powerful discrimination of iCCA *versus* HCC, CIR, and PSC. All our panels, as shown in Figure 2, demonstrated higher AUC values compared to their single biomarker counterparts, thus confirming the validity of our approach.

As mentioned above, four proteins from our MS findings emerged for further validation. For this purpose, we employed ELISA and measured serum levels of SAA1, SAA4, VCAM1, and LRG1 in independent cohorts of patients. Obtained results showed the same trends of expression of all assayed markers in the serum across the iCCA, HCC, and PSC groups, as detected by MS. This underlines that our MS approach is valid and the protein levels are disease-dependent, strengthening the biomarker utility of the proposed proteins.

While our study has several strengths, such as providing a comprehensive comparison of the serum proteomes among iCCA, HCC, CIR, and PSC, it is important to acknowledge also its limitations. The small sample size may have impacted our findings. Despite these limitation, our study fills a crucial gap in the literature by offering insights into the serum proteome alterations associated with these liver pathologies. Additionally, our preliminary analysis serves as a valuable foundation for future research in this area. The statistical tests employed in our study, combined with stringent criteria, identified proteins with at least a 1.5-fold change, signifying significant differences between the compared groups. These findings underscore the reliability of initial proteomic analysis in identifying promising novel biomarker candidates for iCCA. Moving forward, it is imperative to validate these findings in larger-scale studies to further elucidate the clinical significance and utility of the identified biomarkers.

## 4 Conclusion

Our study provides a pioneering comparative analysis of the serum proteomes of iCCA and other liver conditions, including CIR, PSC, and HCC. By employing high-throughput mass spectrometry techniques, we successfully identified a distinct proteomic signature capable of differentiating these liver diseases. Notably, the identification and validation of specific proteins such as SAA1, SAA4, VCAM1, and LRG1 underscores their potential as biomarkers for distinguishing iCCA from HCC, as well as from PSC.

The findings from our study emphasize the need for continued proteomics-driven research to discover novel biomarkers that can improve the diagnosis and management of iCCA. The observed differential expression of proteins like S100A9, haptoglobin, and ICAM2 between iCCA and HCC further highlights the utility of proteomic profiling in refining diagnostic accuracy. Furthermore, the elevated levels of VCAM1 and TEK in HCC patients suggest their role in tumor progression and potential as diagnostic markers.

While our study demonstrates the feasibility and promise of serum proteomics in distinguishing between complex liver diseases, it is essential to validate these findings in larger cohorts. The integration of proteomic data with clinical practice could potentially reduce the reliance on invasive procedures like liver biopsies, thus minimizing associated risks and improving patient outcomes. Moving forward, extensive validation and functional studies are warranted to fully elucidate the clinical relevance of these biomarkers and to establish robust diagnostic tools for early and accurate detection of iCCA and other liver pathologies.

## 5 Materials and methods

### 5.1 Study participants and sampling

This was a cross-sectional, observational, analytical case–control study. Adult subjects with an established diagnosis iCCA, HCC, CIR, PSC (n = 15 each, both for discovery and validation cohorts), undergoing regular clinic follow up or hospitalization at a tertiary care center, namely, the “Prof. Dr. Octavian Fodor” Regional Institute of Gastroenterology and Hepatology Cluj-Napoca, Romania, were prospectively recruited between 2018 and 2023, according to classical diagnosis criteria. Clinical management and decisions on diagnostic tests and medication were at the discretion of the treating physician. The HC group (both discovery and validation cohorts) consisted of 15 subjects referred to our center. Blood samples for the clinical routine analysis and the proteomics analysis were collected during admission as part of hospital protocol. Serum samples for the proteome analysis were aliquoted and stored at −80°C. The study was conducted according to the guidelines of the WMA Declaration of Helsinki and approved by the Ethics Committee of the study center (decision number 18/2021). Written informed consent was sought from all participants prior to inclusion and sample collection.

### 5.2 Sample preparation for proteomics analysis

Blood samples were drawn into tubes with serum separator gel (BD Vacutainer, Franklin Lakes, NJ, USA) and processed following the manufacturer’s guidelines. Serum aliquots were immediately stored at −80°C until analysis.

### 5.3 Depletion of six highly abundant serum proteins

Serum albumin, immunoglobulin gamma, immunoglobulin alpha, serotransferrin, haptoglobin, and alpha-1-antitrypsin were removed using multi-affinity chromatography (MARS6-human) from Agilent Technologies, Waldbronn, Germany, following the manufacturer’s instructions. Post-depletion, samples underwent concentration via trichloroacetic acid precipitation, reaching a final concentration of 15% for the remaining protein fraction. The resulting pellet was then resuspended in a urea/thiourea buffer (8/2 M VWR, Radnor, PA, USA), in accordance with previously established procedures (Ilies et al., 2018; Lucaciu et al., 2023). Subsequently, samples were stored at −80°C until use.

### 5.4 Proteolytic digestion by trypsin

Proteolytic digestion by trypsin followed standard protocol of our proteomics team (Ilies et al., 2018; Lucaciu et al., 2023). Briefly, the protein concentration in the samples was assessed using the microplate Bradford Assay (Invitrogen, Waltham, MA, USA) with bovine serum albumin as the standard protein. For each protein sample, a total of 11 μg was subjected to a series of steps: reduction with dithiothreitol (2.5 mM, 30 min at 37°C), alkylation with iodoacetamide (10 mM, 15 min at 37°C), and proteolytic digestion by trypsin (Merck KGaA, Darmstadt, Germany) at a 1:25 protease-to-protein ratio (overnight at 37°C). The digestion process was halted using 1% acetic acid, and peptide desalting was carried out using an Oasis HLB 96-well μElution plate (Waters Corporation, Milford, MA, USA) in accordance with the manufacturer’s guidelines. The lyophilized peptides were reconstituted in 0.1% formic acid to achieve a final concentration of 0.1 μg/μL before injection.

### 5.5 Proteome profiling by mass spectrometry

Nano-LC-HDMS^E^ analysis also followed standard protocol of our mass spectrometry lab (Lucaciu et al., 2023). In short, peptides (300 ng) were chromatographically separated on an ACQUITY UPLC^®^ M-Class HSS T3 column (Waters Corporation, Milford, MA, USA) over a 120-min period, employing a non-linear gradient ranging from 5% to 85% acetonitrile and 0.1% formic acid at a flow rate of 300 nL/min. Detection of eluted peptides was accomplished using an online-coupled traveling wave ion-mobility-enabled hybrid quadrupole orthogonal acceleration time-of-flight mass spectrometer (SYNAPT G2-Si HDMS, Waters Corporation, Milford, MA, USA). The data acquisition utilized the independent acquisition mode, a feature programmed for parent and product ion measurement by switching between low energy (MS) and elevated energy (MS^E^), with collision voltage ramping set as a default. Two technical replicates were performed for each sample, and raw data were acquired through MassLynx™ Software Version 1.74.2662 (Waters Corporation, Milford, MA, USA). Detailed settings can be found in the Supplementary Methods.

## 5.6 Human proteome database search

LC–HDMS^E^ data underwent processing following established protocols (Lucaciu et al., 2023). In essence, Progenesis QI (v2.0, Waters Corporation, Milford, MA, USA) was employed for automated peak picking and chromatogram alignment. The built-in search engine of the software conducted a spectra search using a Uniprot/Swissprot database (2022) limited to human entries (20,361). The specified parameters included enzyme specificity (trypsin with a maximum of one missed cleavage allowed), fixed modification for carbamidomethylation of cysteine, and variable modification for oxidation of methionine. The search tolerance parameters encompassed a false discovery rate of <4%. Further analysis considered proteins only if they met ion matching requirements of fragments/peptide ≥2, fragments/protein ≥5, and peptides/protein ≥1. Peptide identifications adhered to restrictions of absolute mass error <10 ppm, sequence length >5, and a score >5. Protein relative quantification was conducted based on the summed peptide abundance, utilizing only peptides with no conflicting protein identification.

## 5.7 Statistical analysis

For the dataset capturing study participants’ characteristics at study entry (Table 1): The normality of the dataset was evaluated through the Shapiro–Wilk Test, revealing non-normality (*p* > 0.05). Consequently, non-parametric tests, without normalization, were employed. To assess distinctions between the two groups (iCCA and HCC), the Mann–Whitney test was utilized. Fisher’s exact test was applied to examine differences in qualitative data.

For the dataset encompassing global proteome profiling: Initial proteome data, inherently conforming to a normal distribution, were extracted from ProgenesisQI for proteomics following the software’s default normalization process at the protein level. Subsequently, a minimum of 70% valid values filter was applied to each patient group, and an abundance average was computed between the two technical replicates. The resulting matrix was then imported into MetaboAnalyst 6.0 (https://www.metaboanalyst.ca). To address identified missing values, estimation was performed using the k-nearest neighbors (KNN) algorithm on a feature-wise basis. Finally, a log10 transformation was implemented before embarking on the statistical analysis. Clustering of sampling groups was assessed using partial least squares discriminant analysis (PLS-DA), and proteins with discriminatory potential were identified through PLS-DA variable importance projection (VIP) scores.

Given the normal distribution of the proteomic data, parametric tests were employed. Statistical significance was assessed using the t-test and ANOVA with Tukey’s Honest Significant Difference (HSD) test, with the significance cut-off set at *p* < 0.05. The fold change, calculated as the ratio of two group means, was considered significant at a cut-off level of fold change = 1.5 (log2FC = 0.58).

Biomarker performance was evaluated by Receiver Operating Characteristic (ROC) analysis along with the corresponding Area Under the Curve (AUC) analysis and AUC PLS-DA algorithm. Thecut-off value was set to AUC = 0.7. All statistical analyses were performed using default settings of the MetaboAnalyst 6.0 online omics data analysis platform.

## 6 ELISA

The levels of serum Amyloid A1 (SAA1), Serum Amyloid A4 (SAA4), Vascular cell adhesion protein 1 (VCAM1) and Leucine-rich alpha-2-glycoprotein 1 (LRG1) were determined using sandwich enzyme-linked immunosorbent assays (ELISA) kits. These measurements were made from individual samples of the validation cohort and were performed in duplicate, following the instructions provided with the kits. (SAA1: ABclonal, WoburnMA, USA, catalogue number RK04228, detection range = 0.156–10 ng/mL sensitivity = 0.071 ng/mL, intra-assay precision coefficient of variation (CV) < 10%, inter-assay precision CV < 15%; SAA4: Elabscience Wuhan, China, catalogue number E-EL-H5638, detection range = 78.13–5,000 pg/mL sensitivity = 46.88 pg/mL, intra-assay precision coefficient of variation (CV) < 5%, inter-assay precision CV < 5%; VCAM1: ABclonal, WoburnMA, USA, catalogue number RK00026, detection range = 15.6–1,000 pg/mL sensitivity = 3.85 pg/mL, intra-assay precision coefficient of variation (CV) ≤10%, inter-assay precision CV ≤ 15%; LRG1: ABclonal, WoburnMA, USA, catalogue number RK01800, detection range = 31.2–2000 pg/mL, sensitivity = 15.6 pg/mL, intra-assay precision coefficient of variation (CV) ≤10%, inter-assay precision CV ≤ 15%). For each parameter, a calibration curve was constructed using the protein standard supplied. Absorbance readings were taken with a microplate reader (ClarioStar, BMGLabtech, Ortenberg, Germany), and data acquisition and analysis were performed using the built-in MARS software. A 4-parameter logistic regression model was employed to generate the calibration curve for quantification, and the final concentration was determined by averaging the two measurements. Outliers were tested using Grubb’s test, when significance level was set to alpha = 0.05. Significant outliers were excluded from the dataset and data are presented as mean ± standard deviation (SD).

## Data Availability

The original contributions presented in the study are included in the article/Supplementary Material, further inquiries can be directed to the corresponding author.

## References

[B1] Best J. Bechmann L. P. Sowa J.-P. Sydor S. Dechêne A. Pflanz K. (2020). GALAD score detects early hepatocellular carcinoma in an international cohort of patients with nonalcoholic steatohepatitis. Clin. Gastroenterology Hepatology Official Clin. Pract. J. Am. Gastroenterological Assoc. 18 (3), 728–735. 10.1016/j.cgh.2019.11.012 31712073

[B2] Choi G. H. Jang E. S. Kim J.-W. Jeong S.-H. (2021). Prognostic role of plasma level of angiopoietin-1, angiopoietin-2, and vascular endothelial growth factor in hepatocellular carcinoma. World J. Gastroenterology 27 (27), 4453–4467. 10.3748/wjg.v27.i27.4453 PMC8316901 34366616

[B3] Christensen T. D. Jensen C. Larsen O. Leerhøy B. Hansen C. P. Madsen K. (2023). Blood-based tumor fibrosis markers as diagnostic and prognostic biomarkers in patients with biliary tract cancer. Int. J. Cancer 152 (5), 1036–1049. 10.1002/ijc.34356 36455598

[B4] Claessen M. M. H. Frank P. V. Tytgat K. M. A. J. Siersema P. D. Buuren H. R. van (2009). High lifetime risk of cancer in primary sclerosing cholangitis. J. Hepatology 50 (1), 158–164. 10.1016/j.jhep.2008.08.013 19012991

[B5] Dong L. Lu D. Chen R. Lin Y. Zhu H. Zhang Z. (2022). Proteogenomic characterization identifies clinically relevant subgroups of intrahepatic cholangiocarcinoma. Cancer Cell 40 (1), 70–87.e15. 10.1016/j.ccell.2021.12.006 34971568

[B6] Fox S. B. Turner G. D. Gatter K. C. Harris A. L. (1995). The increased expression of adhesion molecules ICAM-3, E- and P-selectins on breast cancer endothelium. J. Pathology 177 (4), 369–376. 10.1002/path.1711770407 8568591

[B7] Geramizadeh B. Sehat M. Mehrmozayan A. Reza A. R. A. (2021). Annexin expression in cholangiocarcinoma, and metastatic pancreatic ductal adenocarcinoma ‘is it Be helpful for differential diagnosis of these tumors in the liver? Iran. J. Pathology 16 (4), 433–438. 10.30699/IJP.20201.138489.2512 PMC8463754 34567193

[B8] Ho J.-W. Poon R.-T. Tong C.-S. Fan S.-T. (2004). Clinical significance of serum vascular cell adhesion molecule-1 levels in patients with hepatocellular carcinoma. World J. Gastroenterology 10 (14), 2014–2018. 10.3748/wjg.v10.i14.2014 PMC4572324 15237425

[B9] Ignacio R. M. C. Gibbs C. R. Kim S. Lee E.-S. Adunyah S. E. Son D.-S. (2019). Serum amyloid A predisposes inflammatory tumor microenvironment in triple negative breast cancer. Oncotarget 10 (4), 511–526. 10.18632/oncotarget.26566 30728901 PMC6355188

[B10] Ilies M. Kumar Sappa P. Iuga C. A. Loghin F. Salazar M. G. Weiss F. U. (2018). Plasma protein profiling of patients with intraductal papillary mucinous neoplasm of the pancreas as potential precursor lesions of pancreatic cancer. Clin. Chim. Acta 477, 127–134. 10.1016/j.cca.2017.12.008 29221926

[B11] Izquierdo-Sanchez L. Lamarca A. Casta A.La Buettner S. Utpatel K. Klümpen H.-J. (2022). Cholangiocarcinoma landscape in europe: diagnostic, prognostic and therapeutic insights from the ENSCCA registry. J. Hepatology 76 (5), 1109–1121. 10.1016/j.jhep.2021.12.010 35167909

[B12] Jin Z. Kobayashi S. Gotoh K. Takahashi T. Eguchi H. Naka T. 2020. The prognostic impact of leucine-rich α-2-glycoprotein-1 in cholangiocarcinoma and its association with the IL-6/TGF-β1 Axis. J. Surg. Res. 252 147–155. 10.1016/j.jss.2020.03.018 32278969

[B13] Job S. Rapoud D. Dos Santos A. Gonzalez P. Desterke C. Pascal G. (2020). Identification of four immune subtypes characterized by distinct composition and functions of tumor microenvironment in intrahepatic cholangiocarcinoma. Hepatol. Baltim. Md 72 (3), 965–981. 10.1002/hep.31092 PMC7589418 31875970

[B14] Kaplanski G. Farnarier C. Payan M. J. Bongrand P. Durand J. M. (1997). Increased levels of soluble adhesion molecules in the serum of patients with hepatitis C. Correlation with cytokine concentrations and liver inflammation and fibrosis. Dig. Dis. Sci. 42 (11), 2277–2284. 10.1023/a:1018818801824 9398806

[B15] Kim Ju Y. Kim J. Lim Y.-S. Gwak G.-Y. Yeo I. Kim Y. (2022). Proteome multimarker panel for the early detection of hepatocellular carcinoma: multicenter derivation, validation, and comparison. ACS Omega 7 (34), 29934–29943. 10.1021/acsomega.2c02926 36061641 PMC9434733

[B16] Lapitz A. Azkargorta M. Milkiewicz P. Olaizola P. Zhuravleva E. Grimsrud M. M. (2023). Liquid biopsy-based protein biomarkers for risk prediction, early diagnosis, and prognostication of cholangiocarcinoma. J. Hepatology 79 (1), 93–108. 10.1016/j.jhep.2023.02.027 PMC10292605 36868481

[B17] Liang B. Li C. Zhao J. (2016). Identification of key pathways and genes in colorectal cancer using bioinformatics analysis. Med. Oncol. N. Lond. Engl. 33 (10), 111. 10.1007/s12032-016-0829-6 27581154

[B18] Lin M. Liu J. Zhang F. Qi G. Tao S. Fan W. (2022). The role of leucine-rich alpha-2-glycoprotein-1 in proliferation, migration, and invasion of tumors. J. Cancer Res. Clin. Oncol. 148 (2), 283–291. 10.1007/s00432-021-03876-0 35037101 PMC11801043

[B19] Lucaciu L. A. Seicean R. Uifalean A. Iacobescu M. Iuga C. A. Seicean A. (2023). Unveiling distinct proteomic signatures in complicated crohn’s disease that could predict the disease course. Int. J. Mol. Sci. 24 (23), 16966. 10.3390/ijms242316966 38069288 PMC10707401

[B20] Martin-Serrano M. A. Kepecs B. Torres-Martin M. Bramel E. R. Haber P. K. Merritt E. (2023). Novel microenvironment-based classification of intrahepatic cholangiocarcinoma with therapeutic implications. Gut 72 (4), 736–748. 10.1136/gutjnl-2021-326514 35584893 PMC10388405

[B21] Mocan L. P. Ilieș M. Stanca Melincovici C. Spârchez M. Crăciun R. Nenu I. (2022). Novel approaches in search for biomarkers of cholangiocarcinoma. World J. Gastroenterol. 28 (15), 1508–1525. 10.3748/WJG.V28.I15.1508 35582128 PMC9048460

[B47] Mocan L. P. Rusu I. Melincovici C. S. Boşca B. A. Mocan T. Crăciun R. Mihu C. M. (2023). The role of immunohistochemistry in the differential diagnosis between intrahepatic cholangiocarcinoma, hepatocellular carcinoma and liver metastasis, as well as its prognostic value. Diagnostics, 13 (9), 1542. 10.3390/diagnostics13091542 PMC10177238 37174934

[B22] Naryzny S. N. Legina O. K. (2021). Haptoglobin as a biomarker. Biochem. Mosc. Suppl. Ser. B Biomed. Chem. 15 (3), 184–198. 10.1134/S1990750821030069 34422226 PMC8365284

[B23] Patel A. H. Harnois D. M. Klee G. G. LaRusso N. F. Gores G. J. (2000). The utility of CA 19-9 in the diagnoses of cholangiocarcinoma in patients without primary sclerosing cholangitis. Am. J. Gastroenterology 95 (1), 204–207. 10.1111/j.1572-0241.2000.01685.x 10638584

[B24] Pirisi M. Fabris C. Falleti E. Soardo G. Toniutto P. Vitulli D. (1996). Serum soluble vascular-cell adhesion molecule-1 (VCAM-1) in patients with acute and chronic liver diseases. Dis. Markers 13 (1), 11–17. 10.1155/1996/129325 8875114

[B25] Rumgay H. Arnold M. Ferlay J. Lesi O. Cabasag C. J. Vignat J. (2022). Global burden of primary liver cancer in 2020 and predictions to 2040. J. Hepatology 77 (6), 1598–1606. 10.1016/j.jhep.2022.08.021 PMC9670241 36208844

[B26] Sack G. H. Jr (2018). Serum amyloid A–a review. Mol. Med. Camb. Mass.) 24 (1), 46. 10.1186/s10020-018-0047-0 30165816 PMC6117975

[B27] Santamaria M. Pardo-Saganta A. Alvarez-Asiain L. Di Scala M. Qian C. Prieto J. (2013). Nuclear α1-antichymotrypsin promotes Chromatin condensation and inhibits proliferation of human hepatocellular carcinoma cells. Gastroenterology 144 (4), 818–828.e4. 10.1053/j.gastro.2012.12.029 23295442

[B28] Serag W. M. Elsayed B. E. (2021). Annexin A5 as a marker for hepatocellular carcinoma in cirrhotic hepatitis C virus patients. Egypt. Liver J. 11 (1), 32. 10.1186/s43066-021-00101-y

[B29] Shaib Y. H. El-Serag H. B. Davila J. A. Morgan R. McGlynn K. A. (2005). Risk factors of intrahepatic cholangiocarcinoma in the United States: a case-control study. Gastroenterology 128 (3): 620–626. 10.1053/j.gastro.2004.12.048 15765398

[B30] Shaib Hashem B. Yasser E.-S. (2004). The epidemiology of cholangiocarcinoma. Semin. Liver Dis. 24 (02), 115–125. 10.1055/s-2004-828889 15192785

[B31] Shi Y. Deng X. Zhan Q. Shen B. Jin X. Zhu Z. (2013). A prospective proteomic-based study for identifying potential biomarkers for the diagnosis of cholangiocarcinoma. J. Gastrointest. Surg. 17 (9), 1584–1591. 10.1007/s11605-013-2182-9 23868055 PMC3753471

[B32] Shirota T. Ojima H. Hiraoka N. Shimada K. Rokutan H. Arai Y. (2015). Heat shock protein 90 is a potential therapeutic target in cholangiocarcinoma. Mol. Cancer Ther. 14 (9), 1985–1993. 10.1158/1535-7163.MCT-15-0069 26141945

[B33] Soman A. Nair S. A. (2022). Unfolding the cascade of SERPINA3: inflammation to cancer. Biochimica Biophysica Acta (BBA) - Rev. Cancer 1877 (5), 188760. 10.1016/j.bbcan.2022.188760 35843512

[B34] Sun B. B. Suhre K. Gibson B. W. (2024). Promises and challenges of populational proteomics in health and disease. Mol. and Cell. Proteomics 100786, 100786. 10.1016/j.mcpro.2024.100786 PMC11193116 38761890

[B35] Sun X. Ma Q. Cheng Y. Huang H. Qin J. Zhang M. (2023). Overexpression of CHAF1A is associated with poor prognosis, tumor immunosuppressive microenvironment and treatment resistance. Front. Genet. 14, 1108004. 10.3389/fgene.2023.1108004 36968583 PMC10033519

[B36] Torimura T. Ueno T. Kin M. Harada R. Taniguchi E. Nakamura T. (2004). Overexpression of angiopoietin-1 and angiopoietin-2 in hepatocellular carcinoma. J. Hepatology 40 (5), 799–807. 10.1016/j.jhep.2004.01.027 15094228

[B37] Voigtländer T. Metzger J. Husi H. Kirstein M. M. Pejchinovski M. Latosinska A. (2020). Bile and urine peptide marker profiles: access keys to molecular pathways and biological processes in cholangiocarcinoma. J. Biomed. Sci. 27 (1), 13. 10.1186/s12929-019-0599-5 31900160 PMC6941325

[B38] Whitesell L. Lindquist S. L. (2005). HSP90 and the chaperoning of cancer. Nat. Rev. Cancer 5 (10), 761–772. 10.1038/nrc1716 16175177

[B39] Wu D.-C. Wang K.-Yi Wang S. S. W. Huang C.-M. Lee Y.-W. Chen M. I. (2017). Exploring the expression bar code of SAA variants for gastric cancer detection. Proteomics 17 (11). 10.1002/pmic.201600356 28493537

[B40] Yamaguchi N. Tokushige K. Haruta I. Yamauchi K. Hayashi N. (1999). Analysis of adhesion molecules in patients with idiopathic portal hypertension. J. Gastroenterology Hepatology 14 (4), 364–369. 10.1046/j.1440-1746.1999.01857.x 10207787

[B41] Zhang H. Kong Q. Wang J. Jiang Y. Hua H. (2020a). Complex roles of CAMP-PKA-CREB signaling in cancer. Exp. Hematol. and Oncol. 9 (1), 32. 10.1186/s40164-020-00191-1 PMC7684908 33292604

[B42] Zhang M. Yang H. Wan L. Wang Z. Wang H. Ge C. (2020b). Single-cell transcriptomic architecture and intercellular crosstalk of human intrahepatic cholangiocarcinoma. J. Hepatology 73 (5), 1118–1130. 10.1016/j.jhep.2020.05.039 32505533

[B43] Zhang W. Kong H.-F. Gao X.-D. Dong Z. Lu Y. Huang J.-G. (2020c). Immune infiltration-associated serum amyloid A1 predicts favorable prognosis for hepatocellular carcinoma. World J. Gastroenterology 26 (35), 5287–5301. 10.3748/wjg.v26.i35.5287 PMC7504249 32994688

[B44] Zhou J. Lei Yu Gao X. Hu J. Wang J. Dai Z. (2011). Plasma MicroRNA panel to diagnose hepatitis B virus-related hepatocellular carcinoma. J. Clin. Oncol. Official J. Am. Soc. Clin. Oncol. 29 (36), 4781–4788. 10.1200/JCO.2011.38.2697 22105822

[B45] Zhuang C. Wang P. Sun T. Zheng L. Ming L. (2019). Expression levels and prognostic values of annexins in liver cancer. Oncol. Lett. 18 (6), 6657–6669. 10.3892/ol.2019.11025 31807177 PMC6876331

[B46] Zou Y. Xu Yi Chen X. Wu Y. Fu L. Lv Y. (2022). Research progress on leucine-rich alpha-2 glycoprotein 1: a review. Front. Pharmacol. 12, 809225. 10.3389/fphar.2021.809225 35095520 PMC8797156

