# Supplementary material for: Unveiling novel serum biomarkers in intrahepatic cholangiocarcinoma: a pilot proteomic exploration
Source: Front Pharmacol. 2024 Sep 2;15:1440985. doi: 10.3389/fphar.2024.1440985 (PMC11403330; doi:10.3389/fphar.2024.1440985)
Supplement: Supplementary file 1 [file Table1.pdf]

Supplementary Table S1. Baseline characteristics of the study participants

	Discovery cohort	Validation cohort
	iCCA (n=15)	
Age (mean ± SD)	67 ± 8.2	65.1 ± 8.7
Age [range]	52-78	51-84
**Gender, n (%)**		
Males	8 (53.3)	8 (53.33)
Females	7 (46.6)	7 (46.67)
**Environment, n (%)**		
Urban	6 (40)	10 (66.67)
Rural	9 (60)	5 (33.33)
**Underlying disease, n (%)**		
Hepatitis B virus	1 (6.6)	2 (13.33)
Hepatitis C virus		
Alcohol abuse	2 (13.3)	3 (20)
Diabetes	3 (20)	2 (13.33)
Autoimmune		1 (6.6)
No	9 (60)	7 (46.6)
**Nodules, n (%)**		
Single	0 (0)	1 (6.67)
Two or more	3 (20)	5 (33.33)
Not available	12 (80)	9 (60)
**Nodule size, n (%)**		
≤3 cm	1 (6.6)	0 (0)
>3 cm	8 (53.3)	1 (6.67)
Not available	6 (40)	14 (93.33)
**Child-Pugh score, n (%)**		
A		1 (6.67)
B		2 (13.33)
C		
Not available	15 (100)	12 (80)
**METAVIR score, n(%)**		
F0		
F1		
F2		
F3		
F4		1 (6.67)
Not available	15 (100)	14 (93.33)
**Laboratory [normal values] (units), mean ± SD**		
Albumin [3.5 - 5.2] (g/dL)	3.5 ± 0.4	3.63 ± 0.69

Supplementary Table S1. Baseline characteristics of the study participants

	**Discovery cohort**	**Validation cohort**
	**iCCA (n=15)**	
**Laboratory [normal values] (units), median [Q1; Q3]**		
AFP [0.9-7.0] (ng/mL)	3.9 [2.5; 35.85]	6.11 [2.82; 101]
CA 19-9 [2-34] (U/mL)	117.9 [13.5; 158.15]	30 [5; 300]
CEA [0.3-4.7] (ng/mL)	5.1 [2.4; 14.3]	2.63 [1.51; 8.86]
AST [10-50] (U/I)	60.5 [27.75; 80.5]	83 [43.5 ; 152.5]
ALT [10-50] (U/I)	29 [23.75; 108.25]	54 [22; 135]
ɣGT [8-61] (U/I)	128.5 [77.5; 505.25]	195 [162; 369]
AP [40-129] (U/I)	478 [228.25; 937.5]	280 [189.5; 499]
TB [0.1-1.2] (mg/dL)	1.25 [0.8; 4.4]	2.8 [0.85; 8]
PT [11.8-14.9] (sec)	19.2 [17.93; 24.18]	16.1 [14.85; 18.4]
INR [0.8-1.2]	1.15 [1.08; 1.47]	1.19 [1.07; 1.3]
Glucose [82-115] (mg/dL)	112 [94,3; 121.1]	90 [80; 100.5]
Triglycerides [50-165] (mg/dL)	90.5 [81.75; 99.25]	124 [78.5; 170]
CRP [0-0.5] (mg/dL)	2.91 [0.6; 6.38]	2.4 [1.17; 5.52]
Cholesterol [110-220] (mg/dL)	166 [165.5; 190.25]	175.5 [119.75; 198.25]
	**HCC (n=15)**	
Age (mean ± SD)	64.4 ± 8.6	65.33 ± 7.29
Age [range]	50-81	55-82
**Gender, n (%)**		
Males	12 (80)	7 (46.67)
Females	3 (20)	8 (53.33)
**Environment, n (%)**		
Urban	7 (46.6)	11 (73.33)
Rural	8 (53.3)	4 (26.67)
**Underlying disease, n (%)**		
Hepatitis B virus	2 (13.3)	5 (33.33)
Hepatitis C virus	7 (46.6)	4 (26.67)
Alcohol abuse	3 (20)	4 (26.67)
Diabetes	2 (13.3)	0 (0)
No	3 (20)	1 (6.6)
**Nodules, n (%)**		
Single	4 (26.6)	4 (26.67)
Two or more	8 (53.3)	11 (73.33)
Not available	3 (20)	0 (0)
**Nodule size, n (%)**		
≤3 cm	6 (40)	7 (46.67)
>3 cm	7 (46.6)	3 (20)
Not available	2 (13.3)	5 (33.33)

Supplementary Table S1. Baseline characteristics of the study participants

	Discovery cohort	Validation cohort
	**HCC (n=15)**	
**Child-Pugh score, n (%)**		
A	9 (60)	11(73.33)
B	2 (13.3)	2 (13.33)
C	1 (6.6)	2 (13.33)
Not available	3 (20)	
**METAVIR score, n (%)**		
F0		
F1		
F2		1 (6.67)
F3		
F4	15 (100)	14 (93.33)
Not available		
**Laboratory [normal values] (units), mean ± SD**		
Albumin [3.5 - 5.2] (g/dL)	3.4 ± 0.8	3.67 ± 0.62
**Laboratory [normal values] (units), median [Q1; Q3]**		
AFP [0.9-7.0] (ng/mL)	15 [3.85; 34.85]	19 [5.64; 571]
CA 19-9 [2-34] (U/mL)	36.5 [24.7; 125.25]	32.5 [15.95; 51.75]
CEA [0.3-4.7] (ng/mL)	-	1.79 [1.05; 4.13]
AST [10-50] (U/L)	48 [29.5; 70]	63 [42; 112.5]
ALT [10-50] (U/L)	29 [23; 37]	51 [25.5; 67]
ɣGT [8-61] (U/L)	67 [43; 80]	60 [34; 101.5]
AP [40-129] (U/L)	286 [262; 443]	154 [95; 191.5]
TB [0.1-1.2] (mg/dL)	1.3 [0.9; 1.75]	1.2 [0.9; 2.2]
PT [11.8-14.9] (sec)	20.95 [18.5; 22.47]	16.4 [15.75; 18.35]
INR [0.8-1.2]	1.35 [1.14; 1.42]	1.26 [1.17; 1.43]
Glucose [82-115] (mg/dL)	97.7 [88.3; 104]	102 [93.25; 116]
Triglycerides [50-165] (mg/dL)	159 [156; 162]	70.5 [59.25; 78]
CRP [0-0.5] (mg/dL)	0.43 [0.37; 0.68]	0.6 [0.35; 1.27]
Cholesterol [110-220] (mg/dL)	162 [149; 181]	110.5 [92.5; 123]
	**PSC (n=15)**	
Age (mean ± SD)	52.6 ± 14.1	50.93 ± 14.41
Age [range]	24-76	26-78
**Gender, n (%)**		
Males	5 (33.3)	8 (53.33)
Females	10 (66.6)	7 (46.67)
**Environment, n (%)**		
Urban	9 (60)	11 (73.33)
Rural	6 (40)	4 (26.67)

Supplementary Table S1. Baseline characteristics of the study participants

	Discovery cohort	Validation cohort
	PSC (n=15)	
**Underlying disease, n (%)**		
Hepatitis B virus	0 (0)	
Hepatitis C virus	0 (0)	
Alcohol abuse	0 (0)	1 (6.66)
Diabetes	3 (20)	2 (13.33)
No	12 (80)	12 (80)
**Child-Pugh score, n (%)**		
A		2 (13.3)
B		2 (13.3)
C		0 (0)
Not available	15 (100)	11 (73.33)
**METAVIR score, n (%)**		
F0	1 (6.6)	1 (12.5)
F1	0 (0)	0 (0)
F2	4 (26.6)	3 (20)
F3	0 (0)	1 (6.6)
F4	3 (20)	4 (26.6)
No	0 (0)	2 (13.3)
Not available	7 (46.6)	4 (26.6)
**Laboratory [normal values] (units), mean ± SD**		
Albumin [3.5 - 5.2] (g/dL)	4 ± 0.3	3.78 ± 0.54
**Laboratory [normal values] (units), median [Q1; Q3]**		
AFP [0.9-7.0] (ng/mL)	2.15 [1.72; 4.67]	2.5 [1.47; 5.99]
CA 19-9 [2-34] (U/mL)	4.5 [2.75; 6]	7.52 [4.5; 25]
CEA [0.3-4.7] (ng/mL)	-	2.71 [1.06; 7.63]
AST [10-50] (U/L)	31 [23.5; 66.5]	31 [20.5; 64.5]
ALT [10-50] (U/L)	43 [22.5; 59]	42 [22.5; 53]
γGT [8-61] (U/L)	112 [51; 322]	175 [70; 367.5]
AP [40-129] (U/L)	514 [392.5; 853.5]	345 [270; 738.5]
TB [0.1-1.2] (mg/dL)	0.7 [0.6; 1.1]	0.6 [0.4; 1.02]
PT [11.8-14.9] (sec)	16.3 [15.15; 19.65]	15.15 [14.67; 18.77]
INR [0.8-1.2]	1.04 [0.96; 1.18]	1.02 [0.96; 1.44]
Glucose [82-115] (mg/dL)	89.5 [82; 101.75]	85.85 [82; 96.5]
Triglycerides [50-165] (mg/dL)	112 [90.5; 176]	92 [64; 175]
CRP [0-0.5] (mg/dL)	0.41 [0.39; 0.73]	0.6 [0.41; 1.14]
Cholesterol [110-220] (mg/dL)	204 [188.5; 255]	227 [168; 255]

Supplementary Table S1. Baseline characteristics of the study participants

	Discovery cohort	Validation cohort
	CIR (n=15)	
Age (mean ± SD)	64.1 ± 9.1	
Age [range]	51-78	
**Gender, n (%)**		
Males	8 (53.33)	
Females	7 (46.67)	
**Environment, n (%)**		
Urban	10 (66.6)	
Rural	5 (33.3)	
**Underlying disease, n (%)**		
Hepatitis B virus	0 (0)	
Hepatitis C virus	1 (6.6)	
Alcohol abuse	9 (60)	
Diabetes	5 (33.3)	
**Child-Pugh score, n (%)**		
A	8 (53.3)	
B	2 (13.3)	
C	2 (13.3)	
Not available	3 (20)	
**Laboratory [normal values] (units), mean ± SD**		
Albumin [3.5 - 5.2] (g/dL)	3.7 ± 0.3	
**Laboratory [normal values] (units), median [Q1; Q3]**		
AFP [0.9-7.0] (ng/mL)	2.9 [2.9; 3.3]	
CA 19-9 [2-34] (U/mL)	19.25 [10.13; 28.37]	
CEA [0.3-4.7] (ng/mL)	4.29 [3.79; 4.79]	
AST [10-50] (U/L)	36.5 [30.5; 40]	
ALT [10-50] (U/L)	23.5 [19.25; 32.25]	
ɣGT [8-61] (U/L)	74 [25; 115]	
AP [40-129] (U/L)	195 [156; 220.5]	
TB [0.1-1.2] (mg/dL)	0.98 [0.71; 1.65]	
PT [11.8-14.9] (sec)	16.6 [15; 18.1]	
INR [0.8-1.2]	1.24 [1.12; 1.36]	
Glucose [82-115] (mg/dL)	119.5 ± 43.5	
Triglycerides [50-165] (mg/dL)	105 [66; 109]	
CRP [0-0.5] (mg/dL)	1.02 [0.37; 2.18]	
Cholesterol [110-220] (mg/dL)	168 [151; 181]	

Supplementary Table S1. Baseline characteristics of the study participants

	Discovery cohort	Validation cohort
	HC (n=15)	
Age (mean ± SD)	32.1 ± 8.9	35.4 ± 10.46
Age [range]	23-55	23-54
**Gender, n (%)**		
Males	9 (60)	6 (40)
Females	6 (40)	9 (60)
**Laboratory [normal values] (units), mean ± SD**		
Albumin [3.5 - 5.2] (g/dL)	3.8 ± 0.2	4.44 ± 0.39
**Laboratory [normal values] (units), median [Q1; Q3]**		
CRP [0-0.5] (mg/dL)	0.48 [0.35; 0.99]	0.18 [0.11; 0.27]

iCCA = intrahepatic cholangiocarcinoma; HCC = hepatocellular carcinoma; PSC = primary sclerosing cholangitis; CIR = liver cirrhosis; HC = healthy controls; n= number; F0-F4 = fibrosis degree; AFP = alpha-fetoprotein; CA 19-9 = carbohydrate antigen 19-9; CEA = carcinoembryonic antigen; AST = aspartate aminotransferase; ALT = alanine aminotransferase; ɣGT = gamma-glutamyl transferase; AP = alkaline phosphatase; TB = total bilirubin; PT = prothrombin time; INR = international normalized ratio; CRP = C-reactive protein; normal values are in square brackets; SD = standard deviation, Q = quartile
