# Supplementary material for: Unveiling novel serum biomarkers in intrahepatic cholangiocarcinoma: a pilot proteomic exploration
Source: Front Pharmacol. 2024 Sep 2;15:1440985. doi: 10.3389/fphar.2024.1440985 (PMC11403330; doi:10.3389/fphar.2024.1440985)
Supplement: Supplementary file 2 [file Table2.pdf]

Supplementary Table S2. Overview on the abundance of all 646 relatively quantified proteins

No.	Protein name	Gene	[illegible]
[illegible]	[illegible]	[illegible]	[illegible]

Supplementary Table S2. Overview on the abundance of all 646 relatively quantified proteins

		Group→	HC															HCC															iCCA															CIR															PSC														
		Sample→	HC01	HC02	HC03	HC04	HC05	HC06	HC07	HC08	HC09	HC10	HC11	HC12	HC13	HC14	HC15	HCC01	HCC02	HCC03	HCC04	HCC05	HCC06	HCC07	HCC08	HCC09	HCC10	HCC11	HCC12	HCC13	HCC14	HCC15	iCCA01	iCCA02	iCCA03	iCCA04	iCCA05	iCCA06	iCCA07	iCCA08	iCCA09	iCCA10	iCCA11	iCCA12	iCCA13	iCCA14	iCCA15	CIR01	CIR02	CIR03	CIR04	CIR05	CIR06	CIR07	CIR08	CIR09	CIR10	CIR11	CIR12	CIR13	CIR14	CIR15	PSC01	PSC02	PSC03	PSC04	PSC05	PSC06	PSC07	PSC08	PSC09	PSC10	PSC11	PSC12	PSC13	PSC14	PSC15
No.	Protein name	Gene ↓																																																																										
177	Cullin-4B	CUL4B	11860	17737	14650	14250	18705	20759	8184	14042	24955	17755	26160	17200	30445	21054	21184	14044	8831	13109	18421	21172	17688	14427	20252	19424	22360	29504	22740	37441	18699	5325	9713	10130	12463	13262	16930	8088	19635	9710	19967	20309	23145	38513	31956	14200	21341	13183	13984	13358	15389	12228	16165	16316	15772	20026	25766	17709	24652	27681	34587	5668	10638	14429	14910	14953	21547	12879	16943	22204	26535	29019	25213	30520	22553	30534	5405
178	Cystatin-C	CST3																330	12	336	7	12	32	14	8		19	95		130	99	50																42	204		3	11	9	5	58	20	63		23	47		40															
179	Cytochrome b-c1 complex subunit 2_ mitochondrial	UQCRC2	587	338	282	637	952	682	421	564	887	296	1169	715	578	77	1716	185	115	394	368	83	280	492	260	705	521	271	614	483	2037	205	425	296	234	372	206	571	1258	406	967	476	642	1088	1337	106	682	150	136	348	287	393	257	121	50	645	386	273	1200	526	650	302	336	180	100	586	946	371	761	664	545	338	621	56	767	507	326
180	Cytoplasmic dynein 1 heavy chain 1	DYNC1H1	103363	136784	94527	99011	130941	152202	125134	170096	165674	179989	182381	222011	212635	209583	194607	73681	65453	105986	167289	142460	124264	101178	109419	177724	147811	198774	159664	165052	140411	57783	64661	59324	73918	115618	109649	79786	78065	118628	136953	127362	119931	186176	108289	109783	211663	74049	91664	143516	127760	100725	112951	132715	100214	130863	135261	131016	151163	125189	184090	62689	55650	77679	93375	92764	102614	109544	94931	106956	138142	125597	112745	190895	190256	173044	42273
181	D-amino-acid oxidase	DAO	25	57	74	100	52	20	15	53	115	5	12	46	41	29		115	63	40	10	71	65	108	216	8	73	22	18	18	19	56	15	151	62	22	35	73	170	8	97	28	48	51	197		26	51	18	22	24	31	143	95	17	38	48	90	16	98	10	56	34	52	82	64	83	33	113	49	22	46	82	13	30	46	38
182	Dapper homolog 1	DACT1	130	167	90	138	63	134	58	54	20	61	45	31	51	35	33	25	35	134	351	40	52	51	63	85	64	84	158	56	103	94	107	67	80	121	43	48	32	34	138	10	156	66	93		63	9	66	46	162	34	52	9	97	148	96		131	22	54	53	15	56	86	35	51	46	73	82	59	6	50	116	52	25	50
183	DDB1- and CUL4-associated factor 4-like protein 1	DCAF4L1	272	304	145	99	248	272	228	179	168	371	342	322	2346	324	478	170	89	185	11906	476	62	7	3523	146	54	594	164	326	4567	201	153	153	92	76	33	19	138	46464	193	228	91	1329	5136	76279	313	100	1741	182	166	162	51	71	217	85	225	28	342	201	344	247	72	93	91	36	387	25	24	68	100	19	17	45243	227	115	172
184	Death-inducer obliterator 1	DIDO1	106	31	120	67	105	71	123	271	171	27	8	338	100	925	87	18542	89	134	106	84	2037	88	80	24	24	32	149	89	81	200	148	157	217	55	54	127	95	83	66	54	225	28	3158	45	20	1656	11192	95	167	78	3577	65	6799	117	89	38	285	4265	56	196	114	87	255	177	97	45	172	74	172	70	205	59	97	180	220
185	Dehydrodolichyl diphosphate synthase complex subunit DHDDS	DHDDS	481	442	751	674	317	950	470	386	1014	977	706	848	1647	805	360	177	575	696	426	1022	579	401	321	825	874	947	652	1728	1081	432	1105	2759	2671	642	1323	1168	147	228	2081	1039	1157	205	757	2712	1135	688	536	1037	724	1481	1174	651	12774	260	1191	1267	4989	2188	2188	512	759	2101	924	616	155	513	652	1636	4181	1180	1248	878	1804	1658	334
186	Deoxyribonuclease gamma	DNASE1L3	3558	6912	2746	3556	5808	5850	5201	7625	2916	9676	8284	6716	7761	10744	6035	4319	2169	7734	18866	10220	8922	5551	6251	9054	10125	16179	13009	13773	9309	5446	2011	2663	4241	6539	5582	3564	5843	7764	10432	6647	6999	21120	6622	8143	12823	3031	5991	8243	7270	2959	5414	7751	5325	9360	8572	6386	14532	8284	9153	4826	1399	2273	2997	3935	12183	5789	3998	3426	8404	5319	2999	10033	8703	7581	1870
187	Differentially expressed in FDCP 8 homolog	DEF8	5925	10052	6421	5200	8038	11570	7379	8184	7115	10702	7268	10001	11418	10377	8672	2168	2004	4387	5336	5189	3979	2920	2096	6758	2840	6288	2898	3964	2415	5930	2443	1612	3198	4035	3468	3079	1955	1937	3624	3113	3434	4302	859	1932	3851	2538	3015	7333	4589	4566	3398	4070	2459	2598	4007	2037	3048	2571	6809	8009	2191	3865	5028	3054	4538	3078	2605	2731	3748	2978	2476	4489	5996	3411	4610
188	Dihydropyrimidine dehydrogenase [NADP(+)]	DPYD	1856	2757	2575	2649	4048	1901	3330	2135	927	2156	2667	1714	1880	826	949	4541	2257	1582	1379	1473	2501	3623	2836	2540	1949	1926	2136	2150	1731	498	1434	3800	1510	1674	1985	2456	3892	2016	3943	2549	4430	2028	6376	2867	1188	2436	3279	1401	1522	1592	3784	2511	4408	2862	4557	2130	3809	6636	1058	377	2231	1923	1139	1868	1787	2850	1664	2027	1878	2954	2058	939	1289	1247	388
189	Divergent paired-related homeobox	DPRX	785	[illegible]
190	DNA ligase 4	LIG4	3255	[illegible]
191	DNA polymerase epsilon catalytic subunit A	POLE	53483	[illegible]
192	DNA primase small subunit	PRIM1	1668	[illegible]
193	DNA repair protein RAD51 homolog 4	RAD51D		[illegible]
194	DNA repair protein XRCC2	XRCC2	16815	[illegible]
195	DNA/RNA-binding protein KIN17	KIN	2930	[illegible]
196	DNA-directed DNA/RNA polymerase mu	POLM	880	[illegible]
197	DnaJ homolog subfamily B member 3	DNAJB3	1094	[illegible]
198	Dopamine beta-hydroxylase	DBH	4078	[illegible]
199	DPY30 domain-containing protein 2	DYDC2	84	[illegible]
200	Dual oxidase 1	DUOX1	4	[illegible]
201	Dual specificity protein kinase TTK	TTK	268	[illegible]
202	Dynein axonemal assembly factor 11	DNAAF11	142	[illegible]
203	Dynein axonemal heavy chain 10	DNAH10	45377	[illegible]
204	Dynein axonemal heavy chain 14	DNAH14	27954	[illegible]
205	Dynein axonemal heavy chain 17	DNAH17	7574	[illegible]
206	Dynein axonemal heavy chain 2	DNAH2	4035	[illegible]
207	Dynein axonemal heavy chain 3	DNAH3	84931	[illegible]
208	Dynein axonemal heavy chain 7	DNAH7	45874	[illegible]
209	Dystonin	DST	21071	[illegible]
210	E3 ubiquitin-protein ligase HECW1	HECW1	1139	[illegible]
211	EF-hand calcium-binding domain-containing protein 3	EFCAB3	7712	[illegible]
212	EGF-containing fibulin-like extracellular matrix protein 1	EFEMP1	23926	[illegible]
213	EH domain-containing protein 4	EHD4	4106	[illegible]
214	Electron transfer flavoprotein beta subunit lysine methyltransferase	ETFBKMT	825	[illegible]
215	ELKS/Rab6-interacting/CAST family member 1	ERC1	3711	[illegible]
216	Endonuclease domain-containing 1 protein	ENDOD1	37	[illegible]
217	Endoplasmic reticulum chaperone BiP	HSPA5	596	[illegible]
218	EP300-interacting inhibitor of differentiation 1	EID1	38788	[illegible]
219	Ephrin type-A receptor 5	EPHA5	2408	[illegible]
220	Epidermal retinol dehydrogenase 2	SDR16C5	72	[illegible]
221	Epididymal secretory glutathione peroxidase	GPX5	802	[illegible]
222	ER membrane protein complex subunit 2	EMC2	1401	[illegible]
223	Eukaryotic initiation factor 4A-III	EIF4A3	35	[illegible]
224	Eukaryotic peptide chain release factor GTP-binding subunit ERF3B	GSPT2	2118	[illegible]
225	Eukaryotic translation initiation factor 2 subunit 2	EIF2S2	4367	[illegible]
226	Eukaryotic translation initiation factor 4B	EIF4B	18	[illegible]
227	Exocyst complex component 4	EXOC4	22449	[illegible]
228	Exosome complex exonuclease RRP44	DIS3	25483	[illegible]
229	Extracellular matrix protein 1	ECM1	2653	[illegible]
230	Extracellular superoxide dismutase [Cu-Zn]	SOD3		[illegible]
231	Fanconi-associated nuclease 1	FAN1		[illegible]
232	Far upstream element-binding protein 1	FUBP1	480	[illegible]
233	FAS-associated factor 2	FAF2	441	[illegible]
234	Fatty-acid amide hydrolase 2	FAAH2	11593	[illegible]
235	F-box/LRR-repeat protein 6	FBXL6		[illegible]
236	Ferritin heavy chain	FTH1	877	[illegible]
237	Ferritin light chain	FTL	339	[illegible]
238	Fetuin-B	FETUB	2012	[illegible]
239	Fibrinogen alpha chain	FGA	45300	[illegible]
240	Fibrinogen beta chain	FGB	35296	[illegible]
241	Fibrinogen gamma chain	FGG	22369	[illegible]
242	Fibronectin	FN1	176741	[illegible]
243	Fibrous sheath-interacting protein 1	FSIP1	3122	[illegible]
244	Fibulin-1	FBLN1	13826	[illegible]
245	Ficolin-3	FCN3	7475	[illegible]
246	Fructose-1_6-bisphosphatase 1	FBP1	7896	[illegible]
247	Fumarate hydratase_ mitochondrial	FH	10417	[illegible]
248	Fumarylacetoacetase	FAH		[illegible]
249	G patch domain-containing protein 8	GPATCH8	58	[illegible]
250	Galectin-3-binding protein	LGALS3BP	2987	[illegible]
251	Gasdermin-C	GSDMC	683	[illegible]
252	Gelsolin	GSN	160058	[illegible]
253	Girdin	CCDC88A	9851	[illegible]
254	Glial fibrillary acidic protein	GFAP	152	[illegible]
255	Glucose-6-phosphate 1-dehydrogenase	G6PD	1372	[illegible]
256	Glucosylceramide transporter ABCA12	ABCA12	899	[illegible]
257	Glutathione peroxidase 3	GPX3	14274	[illegible]
258	Glutathione S-transferase Mu 2	GSTM2	178	[illegible]
259	Glyceraldehyde-3-phosphate dehydrogenase	GAPDH	673	[illegible]
260	Glycine N-acyltransferase-like protein 1	GLYATL1	2319	[illegible]
261	Glycine N-acyltransferase-like protein 2	GLYATL2	382	[illegible]
262	Glycosyl-phosphatidylinositol-anchored molecule-like protein	GML	1192	[illegible]
263	Golgin subfamily A member 8C	GOLGA8CP	201	[illegible]
264	Golgin subfamily A member 8J	GOLGA8J	3197	[illegible]
265	Golgin subfamily A member 8O	GOLGA8O	10576	[illegible]
266	Golgin subfamily B member 1	GOLGB1	17916	[illegible]
267	Grainyhead-like protein 2 homolog	GRHL2	618	[illegible]
268	GRIP and coiled-coil domain-containing protein 2	GCC2	1666	[illegible]
269	Growth arrest-specific protein 7	GAS7	67	[illegible]
270	Guanine nucleotide-binding protein G(s) subunit alpha isoforms short	GNAS	388	[illegible]
271	Haptoglobin	HP	7915	[illegible]
272	Haptoglobin-related protein	HPR	2796	[illegible]
273	Heat shock protein HSP 90-alpha	HSP90AA1	11743	[illegible]
274	Heat shock protein HSP 90-alpha A2	HSP90AA2P	210	[illegible]
275	Helicase SRCAP	SRCAP	6046	[illegible]
276	Hemoglobin subunit alpha	HBA1	6343	[illegible]
277	Hemoglobin subunit beta	HBB	15120	[illegible]
278	Hemoglobin subunit delta	HBD	23	[illegible]
279	Hemoglobin subunit gamma-1	HBG1	1099	[illegible]
280	Hemopexin	HPX	1205318	[illegible]
281	Heparin cofactor 2	SERPIND1	134826	[illegible]
282	Hepatocyte growth factor activator	HGFAC	1467	[illegible]
283	Hepatocyte growth factor-like protein	MST1	958	[illegible]
284	Histidine protein methyltransferase 1 homolog	METTL18	2214	[illegible]
285	Histidine-rich glycoprotein	HRG	136821	[illegible]
286	Histidine--tRNA ligase_ cytoplasmic	HARS1	11724	[illegible]
287	Histone-lysine N-methyltransferase 2A	KMT2A	12777	[illegible]
288	Histone-lysine N-methyltransferase SMYD3	SMYD3	9435	[illegible]
289	HIV Tat-specific factor 1	HTATSF1		[illegible]
290	HLA class II histocompatibility antigen_ DQ beta 2 chain	HLA-DQB2	32354	[illegible]
291	Homeobox protein Hox-C10	HOXC10	204	[illegible]
292	Homeobox protein Hox-C5	HOXC5	5822	[illegible]
293	Homeobox protein Hox-C6	HOXC6	93	[illegible]
294	Homeobox protein Nkx-3.2	NKX3-2	35657	[illegible]
295	Homeobox protein TGIF1	TGIF1	763	[illegible]
296	Huntingtin-interacting protein 1	HIP1	1101	[illegible]
297	Hyaluronan-binding protein 2	HABP2	11522	[illegible]
298	Immunoglobulin heavy constant alpha 1	IGHA1	1179	[illegible]
299	Immunoglobulin heavy constant alpha 2	IGHA2	3435	[illegible]
300	Immunoglobulin heavy constant delta	IGHD	12393	[illegible]
301	Immunoglobulin heavy constant gamma 1	IGHG1	13455	[illegible]
302	Immunoglobulin heavy constant gamma 2	IGHG2	764	[illegible]
303	Immunoglobulin heavy constant gamma 3	IGHG3	8817	[illegible]
304	Immunoglobulin heavy constant gamma 4	IGHG4	316	[illegible]
305	Immunoglobulin heavy constant mu	IGHM	26307	[illegible]
306	Immunoglobulin heavy variable 1-69D	IGHV1-69D		[illegible]
307	Immunoglobulin heavy variable 3-9	IGHV3-9	1226	[illegible]
308	Immunoglobulin J chain	JCHAIN	1913	[illegible]
309	Immunoglobulin kappa constant	IGKC	76051	[illegible]
310	Immunoglobulin kappa variable 1-12	IGKV1-12		[illegible]
311	Immunoglobulin kappa variable 1D-13	IGKV1D-13	55	[illegible]
312	Immunoglobulin kappa variable 2D-29	IGKV2D-29	70	[illegible]
313	Immunoglobulin kappa variable 3-20	IGKV3-20		[illegible]
314	Immunoglobulin lambda constant 2	IGLC2	44	[illegible]
315	Immunoglobulin lambda constant 7	IGLC7	1059	[illegible]
316	Immunoglobulin lambda variable 1-47	IGLV1-47	11	[illegible]
317	Immunoglobulin lambda variable 1-51	IGLV1-51	7	[illegible]
318	Immunoglobulin lambda variable 3-10	IGLV3-10		[illegible]
319	Immunoglobulin lambda variable 3-19	IGLV3-19		[illegible]
320	Immunoglobulin lambda variable 5-45	IGLV5-45	206	[illegible]
321	Immunoglobulin lambda-like polypeptide 5	IGLL5		[illegible]
322	Inactive N-acetyllactosaminide alpha-1_3-galactosyltransferase	GGTA1	5383	[illegible]
323	Indoleamine 2_3-dioxygenase 1	IDO1	237	[illegible]
324	Inhibitor of Bruton tyrosine kinase	IBTK	11590	[illegible]
325	Insulin-like growth factor-binding protein 3	IGFBP3	772	[illegible]
326	Insulin-like growth factor-binding protein complex acid labile subunit	IGFALS	32255	[illegible]
327	Integrator complex subunit 11	INTS11	13424	[illegible]
328	Integrator complex subunit 6-like	INTS6L	1950	[illegible]
329	Integrin beta-1-binding protein 1	ITGB1BP1	704	[illegible]
330	Inter-alpha-trypsin inhibitor heavy chain H1	ITIH1	173107	[illegible]
331	Inter-alpha-trypsin inhibitor heavy chain H2	ITIH2	371868	[illegible]
332	Inter-alpha-trypsin inhibitor heavy chain H3	ITIH3	70795	[illegible]
333	Inter-alpha-trypsin inhibitor heavy chain H4	ITIH4	319804	[illegible]
334	Intercellular adhesion molecule 2	ICAM2	155	[illegible]
335	Interleukin-1 receptor accessory protein-like 1	IL1RAPL1	1426	[illegible]
336	Intermediate conductance calcium-activated potassium channel protein 4	KCNN4	2431	[illegible]
337	IQCJ-SCHIP1 readthrough transcript protein	IQCJ-SCHIP1	39462	[illegible]
338	Iron-responsive element-binding protein 2	IREB2	16957	[illegible]
339	Kallistatin	SERPINA4	41813	[illegible]
340	KAT8 regulatory NSL complex subunit 3	KANSL3	2060	[illegible]
341	Kelch-like protein 25	KLHL25	1315	[illegible]
342	Keratin_ type I cytoskeletal 14	KRT14	315	[illegible]
343	Keratin_ type I cytoskeletal 16	KRT16	34	[illegible]
344	Keratin_ type I cytoskeletal 20	KRT20	322	[illegible]
345	Keratin_ type II cytoskeletal 1b	KRT77	5277	[illegible]
346	Keratin_ type II cytoskeletal 4	KRT4	456	[illegible]
347	Keratin_ type II cytoskeletal 5	KRT5	534	[illegible]
348	Keratin_ type II cytoskeletal 6A	KRT6A	211	[illegible]
349	Keratin_ type II cytoskeletal 6C	KRT6C		[illegible]
350	Keratin_ type II cytoskeletal 80	KRT80	35867	[illegible]
351	KICSTOR complex protein ITFG2	ITFG2	23	[illegible]
352	Kinesin heavy chain isoform 5C	KIF5C	27233	[illegible]
353	Kinesin-like protein KIF16B	KIF16B	29952	[illegible]
354	Kinesin-like protein KIF20B	KIF20B	31221	[illegible]

Supplementary Table S2. Overview on the abundance of all 646 relatively quantified proteins

No.	Protein name	Group→ / Sample→ / Gene↓	HC01	HC02	HC03	HC04	HC05
355	Kinesin-like protein KIF21B	KIF21B	6316	6391	4507	5027	7050
356	Kinesin-like protein KIF27	KIF27	1900	1071	855	1133	1483
357	Kininogen-1	KNG1	131157	184606	122494	172953	191933
358	Klotho	KL	18425	27410	23426	16017	23942
359	Lactotransferrin	LTF	2029	2549	1483	2114	1881
360	Legumain	LGMN	13843	22479	11622	16739	18856
361	Leucine-rich alpha-2-glycoprotein	LRG1	36489	45275	58004	42382	61656
362	LIM and calponin homology domains-containing protein 1	LIMCH1	384	556	874	511	313
363	Lipopolysaccharide-binding protein	LBP	12575	11227	12406	14110	13951
364	Lipoyl synthase_ mitochondrial	LIAS	2281	4236	1857	2499	2710
365	Liprin-beta-1	PPFIBP1	6517	11153	2803	4589	8689
366	Liver carboxylesterase 1	CES1	28	495	184	160	24
367	Long-chain-fatty-acid--CoA ligase 5	ACSL5	326	339	294	316	277
368	Low affinity immunoglobulin gamma Fc region receptor III-A	FCGR3A	901	2227	1215	971	713
369	Lumican	LUM	62625	21401	31674	45203	87651
370	Lysophospholipid acyltransferase 2	MBOAT2	1458	1605	992	943	523
371	Mannan-binding lectin serine protease 1	MASP1	56	8	56	71	46
372	MAP7 domain-containing protein 1	MAP7D1	20842	26647	19571	21589	30472
373	Matrix extracellular phosphoglycoprotein	MEPE	26969	45069	34204	41381	41569
374	Mediator of RNA polymerase II transcription subunit 11	MED11	7163	5707	8378	6364	9265
375	Meiosis-specific kinetochore protein	MEIKIN	1	6	4	3	
376	Melanoma-associated antigen 12	MAGEA12					
377	Melanoma-associated antigen 2	MAGEA2	1041	1158	662	641	906
378	Melanophilin	MLPH	15745	23233	21329	17607	28702
379	Metalloproteinase inhibitor 1	TIMP1					
380	Metalloreductase STEAP4	STEAP4	1851	1755	1683	2235	2433
381	Methylmalonate-semialdehyde dehydrogenase [acylating]_ mitochondrial	ALDH6A1	6496	11283	7073	9037	8212
382	Microfibril-associated glycoprotein 4	MFAP4	783	770	1037	611	3307
383	Microspherule protein 1	MCRS1	10369	11793	14970	10729	10536
384	Microtubule-associated protein 1A	MAP1A	3554	16460	5057	6959	9592
385	Microtubule-associated protein 2	MAP2	946	1247	530	1098	874
386	Microtubule-associated protein 9	MAP9	3287	3243	5128	4944	3836
387	Mitochondrial ribonuclease P catalytic subunit	PRORP	59	5133	618	42	753
388	Mitogen-activated protein kinase kinase kinase 1	MAP3K1	5169	5706	3863	7223	8798
389	Mitogen-activated protein kinase kinase kinase 15	MAP3K15	13202	20502	16191	17899	19993
390	Mitoguardin 2	MIGA2	142	113	184	229	156
391	Monocyte differentiation antigen CD14	CD14	84702	177904	82121	115452	121390
392	MORN repeat-containing protein 1	MORN1					
393	M-phase inducer phosphatase 1	CDC25A	5079	6323	3926	4475	5446
394	mRNA (2'-O-methyladenosine-N(6)-)-methyltransferase	PCIF1	2781	1917	3077	2437	3421
395	mRNA export factor GLE1	GLE1	9394	12606	12157	10972	14873
396	MYCBP-associated protein	MYCBPAP	524	327	274	601	938
397	Myosin light chain 5	MYL5	3963	4047	3670	4974	6700
398	Myosin regulatory light chain 12B	MYL12B	1119	911	807	394	1498
399	Myosin regulatory light chain 2_ skeletal muscle isoform	MYLPF	729	1565	758	806	1173
400	Myosin-15	MYH15	349	277	466	434	198
401	Myosin-7B	MYH7B	21699	23119	32918	29754	33504
402	Myozenin-2	MYOZ2					
403	N-acetylgalactosamine kinase	GALK2					
404	N-acetylmuramoyl-L-alanine amidase	PGLYRP2	60979	72837	65866	54567	68138
405	NACHT_ LRR and PYD domains-containing protein 14	NLRP14	8454	6173	7160	7928	8264
406	NAD kinase 2_ mitochondrial	NADK2	119	51	138	84	69
407	NADPH:adrenodoxin oxidoreductase_ mitochondrial	FDXR	53	175	16	40	8
408	Nebulin	NEB	11146	19986	10770	11636	14580
409	Nesprin-2	SYNE2	34069	40881	46357	43965	50201
410	Neurocalcin-delta	NCALD	350	130	285	141	108
411	Neurofibromin	NF1	803	1007	777	751	798
412	Neuronal calcium sensor 1	NCS1	7179	10870	12613	9600	11626
413	Neuropeptide S	NPS	66	68	208	177	24
414	NFX1-type zinc finger-containing protein 1	ZNFX1	1257	1843	1425	1620	1435
415	Nicastrin	NCSTN	184	16	144	114	192
416	Nicotinamide N-methyltransferase	NNMT	4375	7102	4490	4664	5564
417	Noelin	OLFM1	972	931	807	1020	890
418	Nucleoredoxin	NXN	4234	7017	4215	12616	4078
419	Nucleoside diphosphate kinase 7	NME7	1325	2055	1096	1392	1637
420	NXPE family member 2	NXPE2	3034	5922	2314	2834	4496
421	Obscurin	OBSCN	55223	42849	59125	62636	79508
422	ORC ubiquitin ligase 1	OBI1	115	47	57	192	295
423	Paired box protein Pax-9	PAX9	1789	5538	1833	2972	3613
424	Pantetheinase	VNN1	11792	14169	10979	11221	10431
425	PAS domain-containing serine/threonine-protein kinase	PASK	8514	1276	5004	5829	10241
426	Peptidase inhibitor 16	PI16	30	5	8	5	4
427	Peptidyl-prolyl cis-trans isomerase FKBP4	FKBP4	1844	3354	1454	2005	1241
428	Peroxiredoxin-2	PRDX2	44	633	256	84	1517
429	Peroxisomal 2_4-dienoyl-CoA reductase [(3E)-enoyl-CoA-producing]	DECR2	1452	1119	1450	1511	1458
430	Peroxisomal trans-2-enoyl-CoA reductase	PECR	127	362	173	195	106
431	Phosphatidylcholine-sterol acyltransferase	LCAT	1293	1241	1053	1254	1187
432	Phosphatidylinositol 4_5-bisphosphate 3-kinase catalytic subunit alpha isoform	PIK3CA	264	657	454	8	13
433	Phosphatidylinositol 4-phosphate 3-kinase C2 domain-containing subunit beta	PIK3C2B	12480	13881	14050	14619	15498
434	Phosphatidylinositol 5-phosphate 4-kinase type-2 alpha	PIP4K2A	235	289	435	252	223
435	Phosphatidylinositol 5-phosphate 4-kinase type-2 gamma	PIP4K2C	810	165	685	585	1069
436	Phosphatidylinositol-glycan-specific phospholipase D	GPLD1	7969	11527	10551	8341	8800
437	Phospholipid transfer protein	PLTP	34077	37721	33036	39164	45475
438	Phospholipid-transporting ATPase IH	ATP11A	14462	24938	6449	9603	51812
439	Phospholipid-transporting ATPase VB	ATP10B	7569	7662	8192	7981	9792
440	Pigment epithelium-derived factor	SERPINF1	47150	61987	40499	55787	55060
441	Plasma kallikrein	KLKB1	76970	87016	80137	85848	74870
442	Plasma protease C1 inhibitor	SERPING1	168640	240202	159407	209260	234170
443	Plasma serine protease inhibitor	SERPINA5	629	743	253	749	517
444	Plasminogen	PLG	338388	424059	338424	344500	365060
445	Plasminogen-like protein A	PLGLA	1158	905	786	589	1133
446	Plastin-2	LCP1	6582	7922	7979	6236	6637
447	Platelet basic protein	PPBP	10764	18930	9192	12648	19219
448	Platelet factor 4	PF4	10822	18194	7797	11988	16691
449	Pleckstrin homology domain-containing family S member 1	PLEKHS1	120	113	169	487	203
450	Plectin	PLEC	84940	123647	84814	92864	120415
451	Plexin-B2	PLXNB2	763	14	435	1075	1705
452	Polypeptide N-acetylgalactosaminyltransferase 15	GALNT15	399	188	256	281	302
453	Polypeptide N-acetylgalactosaminyltransferase 17	GALNT17	1035	587	1099	1959	1377
454	POM121 and ZP3 fusion protein	POMZP3	111	133	216	228	139
455	POTE ankyrin domain family member E	POTEE	13894	13907	13029	14849	18515
456	PRAME family member 8	PRAMEF8	2204	4064	3236	2471	2926
457	Prefoldin subunit 1	PFDN1	2312	2241	1967	3875	4983
458	Pregnancy zone protein	PZP	56341	72451	62746	51391	77371
459	Prelamin-A/C	LMNA	2226	3975	1483	2511	2698
460	PRELI domain-containing protein 2	PRELID2	7086	18247	6573	11034	18679
461	Probable asparagine--tRNA ligase_ mitochondrial	NARS2	4440	3461	3496	2790	4207
462	Probable ATP-dependent RNA helicase DDX17	DDX17	3806	4724	5296	6384	4578
463	Probable ATP-dependent RNA helicase DDX20	DDX20	243	214	1209	614	349
464	Probable non-functional immunoglobulin kappa variable 2D-24	IGKV2D-24	103	40	30	58	84
465	Probable non-functional T cell receptor beta variable 7-1	TRBV7-1	522	14	151	350	488
466	Probable rRNA-processing protein EBP2	EBNA1BP2	525	596	545	612	384
467	Probable serine carboxypeptidase CPVL	CPVL	7227	15806	7041	8610	11051
468	Probable ubiquitin carboxyl-terminal hydrolase FAF-X	USP9X	6109	5532	11195	9035	8793
469	Probable ubiquitin carboxyl-terminal hydrolase FAF-Y	USP9Y	3644	6236	4801	3752	3833
470	Profilin-1	PFN1	1422	2502	922	1029	1646
471	Progesterone-induced-blocking factor 1	PIBF1	13747	24770	12060	15579	20460
472	Proline/serine-rich coiled-coil protein 1	PSRC1	50436	64401	48551	57186	70995
473	Proline-rich protein 15-like protein	PRR15L	129	143	289	156	171
474	Properdin	CFP	9762	12514	10285	10443	12445
475	Prostaglandin-H2 D-isomerase	PTGDS	152	131	164	184	112
476	Proteasomal ATPase-associated factor 1	PAAF1	5989	10615	5400	7254	7741
477	Protein AMBP	AMBP	97383	129905	115838	82759	123545
478	Protein C3orf33	C3orf33	322	325	301	421	296
479	Protein CIP2A	CIP2A	7329	9709	8030	7165	9817
480	Protein FAM153B	FAM153B	795	1178	2312	1321	918
481	Protein FAM210A	FAM210A	156	241	104	186	124
482	Protein FAM3D	FAM3D	21509	26739	22459	27735	33256
483	Protein hinderin	KIAA1328	2021	1540	2988	2973	3275
484	Protein Hook homolog 1	HOOK1	1264	2113	1129	1419	1895
485	Protein kinase C delta type	PRKCD	4771	6921	6550	7156	9042
486	Protein kinase C theta type	PRKCQ	8839	577	4583	6497	12627
487	Protein LTO1 homolog	LTO1	641	677	852	533	799
488	Protein MMS22-like	MMS22L	28676	35007	26353	32519	32862
489	Protein phosphatase 1 regulatory subunit 12C	PPP1R12C	202	230	238	48	33
490	Protein phosphatase 1D	PPM1D	5149	6286	3962	5146	5626
491	Protein piccolo	PCLO	4550	4677	3075	3679	4601
492	Protein S100-A8	S100A8	1916	1789	1344	2884	3650
493	Protein S100-A9	S100A9	111	247	387	127	54
494	Protein S100-B	S100B	2659	1650	1346	1079	2154
495	Protein shortage in chiasmata 1 ortholog	SHOC1	11835	12812	11625	11889	17657
496	Protein SOX-15	SOX15	5998	11448	4371	6884	8763
497	Protein Z-dependent protease inhibitor	SERPINA10	36511	42147	32947	36916	48097
498	Protein ZGRF1	ZGRF1	21327	21899	14884	18844	31949
499	Protein-arginine deiminase type-6	PADI6	119	119	255	147	78
500	Protein-tyrosine kinase 2-beta	PTK2B	1047	2581	2225	1656	2620
501	Protein-tyrosine kinase 6	PTK6					
502	Prothrombin	F2	189357	212447	184112	183058	209763
503	Protocadherin beta-6	PCDHB6	2250	3287	1973	1687	1871
504	PTB domain-containing engulfment adapter protein 1	GULP1	11999	18687	21246	16571	23234
505	PTB-containing_ cubilin and LRP1-interacting protein	PID1	2011	912	1141	1402	1753
506	Putative ciliary rootlet coiled-coil protein-like 1 protein	CROCCP2	21	23	24	16	27
507	Putative DENN domain-containing protein 10 B	DENND10P1	1751	1805	2256	4371	2716
508	Putative heat shock protein HSP 90-beta 2	HSP90AB2P	848	1023	1178	921	821
509	Putative inactive phosphatidylinositol 4-kinase alpha-like protein P1	PI4KAP1	3223	1151	1344	1530	3080
510	Putative uncharacterized protein FRMD6-AS1	FRMD6-AS1	239	280	248	246	247
511	Pyruvate kinase PKM	PKM	73278	85229	105034	96354	98639
512	Queuosine salvage protein	C9orf64	661	584	1620	1329	989
513	Rab GTPase-binding effector protein 1	RABEP1	7345	20605	10208	9994	12463
514	Ras association domain-containing protein 2	RASSF2	86	142	164	385	212
515	RB1-inducible coiled-coil protein 1	RB1CC1	1918	3336	1256	1352	1808
516	Regulator of G-protein signaling 10	RGS10	914	1428	924	1009	863
517	Required for excision 1-B domain-containing protein	REX1BD	529	654	314	697	934
518	Retinoblastoma-like protein 1	RBL1	4744	4952	5659	4641	6273
519	Retinol-binding protein 4	RBP4	64965	47481	26828	62568	63846
520	Rho GTPase-activating protein 10	ARHGAP10	232	410	239	181	64
521	Rho guanine nucleotide exchange factor 17	ARHGEF17	439	361	360	319	344
522	Rhodopsin kinase GRK7	GRK7	4986	4844	4852	4400	4221
523	Ribosomal protein S6 kinase delta-1	RPS6KC1	10467	13008	12525	11203	15391
524	RNA-binding motif protein_ X chromosome	RBMX	1853	2170	1724	2770	3028
525	Rootletin	CROCC	28745	43838	31644	36513	50636
526	Rotatin	RTTN	19356	21354	24701	23983	28031
527	Ryanodine receptor 3	RYR3	15379	24553	18565	17340	20284
528	Sacsin	SACS	1654	1544	2701	2259	1641
529	S-adenosylmethionine decarboxylase proenzyme	AMD1	1099	1873	2408	1483	1813
530	Schlafen-like protein 1	SLFNL1	58	99	22	33	18
531	Sciellin	SCEL	1758	2446	2795	3032	2619
532	Sclerostin	SOST	3660	5429	4233	2946	4638

Supplementary Table S2. Overview on the abundance of all 646 relatively quantified proteins

		Group→	HC															HCC														
		Sample→	HC01	HC02	HC03	HC04	HC05	HC06	HC07	HC08	HC09	HC10	HC11	HC12	HC13	HC14	HC15	HCC01	HCC02	HCC03	HCC04	HCC05	HCC06	HCC07	HCC08	HCC09	HCC10	HCC11	HCC12	HCC13	HCC14	HCC15
No.	Protein name	Gene ↓																														
533	Secernin-2	SCRN2	31	28	44	16	17	22	9	5	21			72		32	14	633	125	578	79	119	623	71	353	22	44	125		33	9	82
534	Selenoprotein P	SELENOP	2963	2342	3052	3446	2569	2837	4318	4858	3061	5257	5100	4412	2728	5022	4047	5342	3805	3857	2417	1838	3516	3316	4773	4704	4321	5673	2131	3114	2675	1342
535	Septin-6	SEPTIN6	2426	3771	4495	2685	4092	2561	3940	3874	2701	3009	3570	2470	2854	2643	4744	1518	4143	2345	1451	3562	3929	4604	4892	2511	4084	2158	4831	4602	4387	2128
536	Serine/threonine-protein kinase MRCK beta	CDC42BPB	711	2078	2198	978	1202	2906	2545	1565	2740	1065	2811	3297	2092	2517	4828	1458	1306	1649	1991	3829	4622	3463	2829	4415	5409	7145	1878	4134	5845	1194
537	Serine/threonine-protein kinase TAO1	TAOK1	720	979	732	1108	969	476	707	870	872	704	1879	789	833	49	1626	2066	1023	730	348	495	1722	454	1743	2072	1139	1432	576	1258	934	488
538	Serine/threonine-protein kinase TAO3	TAOK3	32	10	61	118	75	10	89	9	32		20	10			28	609	160	1166	346	75	296	66	278	74	68	66	29	593	183	317
539	Serine/threonine-protein kinase/endoribonuclease IRE1	ERN1	503	290	165	242	272	233	83	266	122	358	592	432	672	431	393	148	191	1038	3669	1147	564	400	1073	1848	1125	4147	296	2230	1702	710
540	Serine-threonine kinase receptor-associated protein	STRAP	3135	7756	1825	2948	5322	5401	5123	6947	2997	10385	6238	6303	6549	8940	8021	4029	1921	6728	20034	10974	6605	4076	4828	8323	8074	12604	8076	9589	3620	4094
541	Serotransferrin	TF	54074	109362	54992	70999	66707	79512	65163	108628	795509	156233	111543	110671	93273	108079	71627	61952	90037	133729	224276	810169	101179	76077	115418	286383	166315	219022	151648	328049	108596	52924
542	Serpin B3	SERPINB3	2136	2658	3662	2302	2446	4621	2435	1894	4721	4059	2945	3034	3158	5279	4781	1354	1717	1292	1789	3025	1324	3595	2009	3206	2708	2346	5046	2751	2285	1209
543	Serpin B9	SERPINB9	4001	7072	2899	3665	5988	5340	5232	9513	4662	10478	7043	8676	7212	9141	5385	816	2711	6784	17922	12388	6049	5969	5821	12437	11597	14827	11030	12486	7603	5635
544	Serum amyloid A-1 protein	SAA1	4105	4994	5943	4217	5516	7008	4619	6434	7563	7338	6866	8392	5268	6484	8726	4229	5060	6213	8434	4787	6112	8292	5194	4479	4313	8259	7877	4324	9555	4075
545	Serum amyloid A-2 protein	SAA2	120	215	128	59	77	60	111	60	55	107	14	184	56	51	77	235	144	327	295	284	276	95	169	112	128	206	113	121	49	233
546	Serum amyloid A-4 protein	SAA4	19366	23779	20357	22746	20715	25405	23333	25402	33556	23414	26640	31086	17971	19485	25331	13166	16124	10872	5928	17561	30156	31606	19421	22524	16287	13666	12994	10900	12737	10994
547	Serum amyloid P-component	APCS	49859	48618	64143	49449	75691	77295	60603	50920	64872	54548	68938	65259	87899	84013	135257	24472	37466	24043	24286	36851	30927	61680	43336	51302	44569	44378	97108	52664	41820	32091
548	Serum paraoxonase/arylesterase 1	PON1	42574	111570	124091	131981	118534	174707	93739	138838	123686	212039	132175	198215	228039	211678	188767	34832	70383	54888	20295	49713	88221	76075	54463	64955	159013	61470	84403	177632	43713	25922
549	Sex hormone-binding globulin	SHBG	11081	13543	10498	6557	11828	16725	20390	21441	62136	24398	24652	14046	12333	10204	3939	22509	8324	14606	15129	26604	23363	10536	28862	19324	28024	38929	11836	36109	18189	7486
550	SH2 domain-containing protein 6	SH2D6	322	1362	257	549	1077	874	573	732	112	1016	605	506	682	809	479	751	460	1453	3632	1657	1013	498	538	1124	1379	1894	1517	1334	784	696
551	Sialic acid-binding Ig-like lectin 9	SIGLEC9	28073	36937	30038	29411	34733	46739	40936	54959	74283	45093	61390	80746	68127	61160	56794	13639	16041	15254	15512	31513	28272	25942	32648	47101	37426	37744	28161	35851	25088	10302
552	Signal-induced proliferation-associated 1-like protein 1	SIPA1L1	26526	47200	35212	33594	42957	40619	40135	34175	45709	46444	57589	35448	49222	28454	44263	36229	29727	28909	34357	34828	49056	40819	63432	49800	56605	50652	47819	55468	47035	18459
553	Small G protein signaling modulator 1	SGSM1	328	119	486	275	143	89	188	105	628	55	267	117	132	23	183	3	304	95		250	38	279	250	12	241	166	393	242	57	141
554	Small muscular protein	SMPX	2536	2668	2492	3520	2905	3120	3755	3459	1594	2968	4447	3388	4102	3195	3868	731	2898	5007	4636	3094	3496	3821	4777	3170	3527	6063	3794	6109	7214	1277
555	Sorting nexin-4	SNX4	5246	2335	3416	4059	5123	4665	5549	8899	3096	4387	3521	9719	7770	16242	4586	2136	3652	1625	1586	3373	2657	4506	3136	2855	4015	1387	2731	2678	2677	1852
556	Spectrin beta chain_ non-erythrocytic 2	SPTBN2	1502	2137	1070	1426	1928	1552	1388	2266	954	2688	1914	1702	1916	2612	1085	2812	1482	2919	5039	3798	3228	1882	2264	2826	3382	4277	3307	3522	2625	2509
557	Spectrin beta chain_ non-erythrocytic 5	SPTBN5	4157	2121	2168	3705	1837	3255	3543	3672	2468	3265	5149	5034	6127	3851	6750	1556	1617	938	321	1224	998	3886	1721	1707	3373	935	2420	1602	454	1454
558	Sperm flagellar protein 2	SPEF2	18268	37599	27881	29854	27383	29295	27806	38840	46291	65435	48172	42526	45215	43565	43670	13831	26405	35276	59693	41238	25674	34448	36935	55353	46918	54031	65144	54837	45390	16060
559	Sperm-associated antigen 5	SPAG5	4184	6526	4548	3037	7530	9130	6225	4832	5524	8589	7023	4332	9660	9472	10024	5740	3305	5166	9103	5467	6237	4975	6912	5529	5954	9560	10268	9084	10124	3105
560	Spermidine synthase	SRM	9399	14442	15680	13277	8136	16565	14115	9936	15352	12196	14211	9408	11659	11807	11137	1449	7276	8188	12215	14532	11240	14361	7914	17459	16813	22187	6810	10277	14085	3141
561	Spindle assembly abnormal protein 6 homolog	SASS6	376	1289	1552	332	255	215	4216	897	799	397	915	238	956	64	1329	219	165	106	22	481	226	4224	1764	1487	417	209	995	138	3443	276
562	Squalene synthase	FDFT1	4541	4376	3335	4858	8499	4210	5999	4514	3618	2941	5854	4641	4416	1570	4899	9018	3808	2793	1847	2371	4834	5216	5669	2876	4305	2452	2667	3840	1599	3053
563	Structural maintenance of chromosomes protein 1B	SMC1B	1076	868	828	1459	2007	1634	1135	1424	1870	1579	2078	2088	1534	1622	2925	762	696	1094	914	1811	1206	1576	1244	1886	1802	1211	1785	1064	3665	520
564	Succinate dehydrogenase [ubiquinone] iron-sulfur subunit_ mitochondrial	SDHB	1012	2059	749	889	1350	1079	860	1785	689	833	858	1417	707	816	542	2031	1686	2512	5433	4040	2891	2362	3922	2427	3400	5110	4049	4334	2692	1586
565	Superoxide dismutase [Cu-Zn]	SOD1																262	7	116	133	70	47			55	22	67	33		65	61
566	Superoxide dismutase [Mn]_ mitochondrial	SOD2	53	73	115	29	204	85	166	162	184	28	233	65	30	29	102	626	258	16104	11255	2824	3324	173	4857	459	318	23908	72	13448	6882	761
567	Symplekin	SYMPK	47349	102224	33168	48099	80141	79340	75709	114222	62921	105707	85969	105498	109271	110592	89818	76577	45136	109883	223240	156492	137835	93183	127763	157159	168937	250112	175671	213772	143117	56178
568	T cell receptor beta variable 24-1	TRBV24-1	8888	8013	3332	4949	10709	13439	19704	16706	4039	17025	14787	9857	7035	10322	6120	8234	5101	13213	27169	12588	18563	9881	11495	25675	17535	24318	15673	19116	12705	3149
569	T cell receptor beta variable 7-6	TRBV7-6	170	233	250	226	225	78	220	92	31	58	56	58	107	12	44	2393	423	572	109	76	447	527	244	227	133	93	8	137	79	116
570	T-complex protein 11 X-linked protein 1	TCP11X1	2463	10181	3595	2786	8356	5422	4931	6470	4561	6898	7833	7086	7093	4671	12029	668	1048	2305	4095	3861	2655	3038	1783	4078	3892	3734	5596	7450	2736	2778
571	T-complex protein 11 X-linked protein 2	TCP11X2	120	138	193	69	58	69	89	58	374	199	430	59	34	73	170	87	313	37	11	98	37	215	209	83	114	46	156	332	143	165
572	Tenascin-X	TNXB	329	444	400	431	619	375	534	361	254	116	596	87	226	37	123	2470	623	1522	1741	544	2223	387	1437	720	747	1984	209	1880	1653	630
573	Testis-expressed protein 52	TEX52																		740	2466	372		6		592	77	2089	15	255	156	152
574	Testis-expressed protein 54	TEX54	306		112	136	221	254	188	681		133		528	504	1095	50															
575	Tether containing UBX domain for GLUT4	ASPSCR1	12848	29482	19116	19029	19938	21425	32309	41674	29338	31355	27571	23554	31861	35888	25505	43287	21999	39464	55109	28729	56416	32675	40351	33331	33723	45066	40238	47284	43616	9341
576	Tetranectin	CLEC3B	14956	14375	10675	14992	18737	16860	16329	22739	7595	17465	24789	22916	20684	24466	21375	18856	8647	22014	30123	13131	20386	10792	17413	25670	23508	27991	10954	20556	19385	9020
577	Tetratricopeptide repeat protein 21B	TTC21B																222	245	281	221	50	84	66	278	19	52	100	53	28	21	20
578	Thioredoxin domain-containing protein 15	TXNDC15	36	57	147	36	63	9	29	51	23		36	12	41			423	127	204	127	101	278	195	169	62	127	103	146	135	140	74
579	Thioredoxin-like protein 1	TXNL1	13224	11944	13595	13400	16648	19350	14385	18730	16545	13361	17891	22137	18762	19699	20284	3602	6921	5190	667	5275	7536	14115	11479	11156	15654	2717	14947	6234	8591	4346
580	Thrombospondin type-1 domain-containing protein 4	THSD4	140	56	66	81	39	92	44	97	153	50	27	45	30	132	19	1038	124	159	79	46	35	44	146	15	8	13	3	13	104	162
581	Thrombospondin-1	THBS1	50155	63789	59638	53282	64876	70171	71106	57522	77337	83035	67726	69543	78039	84363	89270	23891	27597	28022	35751	41422	32783	44277	42378	66660	41570	39145	63832	50789	43597	23379
582	Thymidylate synthase	TYMS																305	127	4		51	99		13	22	248	17	101			1
583	Thyroxine-binding globulin	SERPINA7	29663	50382	40533	40979	47481	54390	52763	46271	96965	53195	72512	50277	56698	67795	59164	23183	28385	64323	35385	69953	85592	41936	49297	53576	73938	123556	48636	103703	109334	19937
584	Tigger transposable element-derived protein 4	TIGD4	5530	10200	3158	6061	7383	6853	5923	9516	5951	15719	10697	9729	10973	7722	5798	6265	4638	10462	21673	11365	10832	6068	9760	15505	12328	19907	14225	17041	10528	6281
585	Titin	TTN	83661	89036	76115	80107	93145	115172	125673	130368	123601	128268	160571	132701	143937	141193	150378	58966	54121	90653	120542	105184	120081	85789	99685	154145	125282	157439	128296	153269	126802	64005
586	Torsin-1A	TOR1A	1636	1087	1103	2220	2421	1983	1286	2014	2174	1856	2165	2781	2027	1544	3344	428	783	1561	1565	2243	1181	2195	1752	2294	2474	1841	2265	1614	5848	679
587	Transcription cofactor vestigial-like protein 1	VGLL1	1855	798	533	1386	1813	2462	906	1799	1141	2161	2787	3259	3108	1182	2119	131	112	101	20	285	99	465	27	88	261	64	491	68	4	1001
588	Transcription factor 7-like 2	TCF7L2	15814	19388	11758	16067	18905	20697	16846	21112	15300	24440	28378	23308	30350	24173	29038	4843	9909	17794	63589	17063	11633	14536	20135	18811	18581	26698	27404	41046	36727	7741
589	Transcription factor ETV7	ETV7	2663	3049	3203	2773	2752	3225	3771	3968	5138	3353	4716	4462	3543	2789	4474	1003	1858	1355	1011	2584	3340	2282	2751	3942	2645	2340	1593	2075	1941	1315
590	Transcriptional adapter 2-alpha	TADA2A	3852	4389	2992	4113	4996	4971	5190	5896	2380	7140	6060	4791	4494	6481	7329	1301	2257	5480	8794	4209	4298	2686	3491	6359	4480	8592	2825	8026	5298	1666
591	Transforming growth factor-beta-induced protein ig-h3	TGFBI	839	624	190	371	258	1387	176	480	619	501	409	705	263	349	437	648	317	507	1819	767	1449	381	907	1136	1286	1956	972	1378	633	1174
592	Transient receptor potential cation channel subfamily M member 1	TRPM1	2315	3795	3856	3076	3436	4127	4374	3948	6486	2509	4723	3568	3165	1953	2694	8436	3010	5332	5090	4817	6896	3870	4839	5432	4992	7530	3438	7409	9559	2782
593	Transient receptor potential cation channel subfamily M member 6	TRPM6	173	120	76	128	66	119	140	247	23	137	689	333	85	73	32	395	105	1217	14981	487	289	55	958	332	320	2297	61	437	3016	274
594	Transmembrane and death domain protein 1	TMDD1	739	397	509	871	801	903	332	377	1359	930	1109	1649	691	794	1466	16	308	633	548	1069	263	1170	433	883	751	282	1170	477	2654	254
595	Transthyretin	TTR	624663	563607	405893	623290	704886	839344	669712	731023	532150	1000644	999576	1057604	1396212	1113099	1463989	130658	209390	184925	217989	330185	168630	475772	262012	508164	550245	333966	631682	442658	181621	297709
596	Trinucleotide repeat-containing gene 18 protein	TNRC18	197	185	372	78	183	624	201	105	428	231	163	96	270	219	114	166	104	77	241	449	95	265	35	31	313	43	387	67	47	257
597	Trinucleotide repeat-containing gene 6A protein	TNRC6A	266	182	354	305	130	25	132	54	83	17	60	126	132	31	175	3390	2259	4840	5413	4388	6103	2584	4818	2519	4342	7328	1669	4267	3856	2225
598	tRNA N(3)-methylcytidine methyltransferase METTL6	METTL6	579	539	1121	693	636	731	1249	883	700	699	815	478	841	651	819	218	594	251	204	475	229	1327	406	454	568	121	1595	204	991	650
599	Trypsin-2	PRSS2	2706	1102	2743	3047	3368	3373	3708	3647	4347	4323	4587	1850	3453	2269	3699	1920	2703	2248	2391	2261	2762	5494	3140	2624	3193	1584	6478	2892	5240	1680
600	TSC22 domain family protein 3	TSC22D3	1616	1526	2014	2385	2297	1541	2099	1682	2344	913	2102	2739	1488	1364	1962	5072	4294	3166	2457	4042	4255	4245	6505	2004	4518	2588	4117	4585	8983	1382
601	t-SNARE domain-containing protein 1	TSNARE1	95914	157291	114917	103115	183692	168473	167767	164376	180207	202769	250595	189265	241456	175674	284251	58440	76966	42899	24397	64626	100229	105961	114019	132560	135613	61012	125504	116198	102547	58413
602	Tumor protein 63	TP63	6622	6406	5662	6510	9748	8539	6770	7214	7829	7820	11622	9333	7276	6104	10321	3769	4456	4868	4830	6214	6103	8028	7023	7911	10065	4990	9042	7274	11246	2701
603	Twinkle mtDNA helicase	TWNK	21	16	14	68	16	15	10	31	382	9	21	12	4	11	22	73	128	3765	3906	493	247	47	1349	591	38	6020	138	4662	7514	244
604	Tyrosine-protein kinase Tec	TEC	149	68	153	98	116	355	396	407	1109	158	944	486	311	9	85	76	615	11661	19545	1865	3575	1147	7961	20696	1575	11759	1052	12769	20733	1292
605	Ubiquitin conjugation factor E4 B	UBE4B	30838	41069	36031	31166	37294	43331	43021	45628	49458	47980	57205	52126	52054	50380	49510	32018	37061	24347	28082	30972	41630	43614	54643	44556	52469	40882	46068	42901	46048	21782
606	Ubiquitin D	UBD	3561	3529	3786	3432	3538	4190	5115	4868	6566	4365	4449	2357	4323	3741	3930	1441	3086	2399	2704	2541	2791	5919	2992	3201	3397	1371	7237	3023	4666	2182
607	Ubiquitin domain-containing protein 1	UBTD1	1160	675	1075	815	210	1583	1521	1191	1737	1163	2372	2189	1659	2701	1887	1015	162	11	6	368	9	444	30	259	330	19	482	54	139	143
608	Ubl carboxyl-terminal hydrolase 18	USP18	1689	2254	2286	722	836	2304	2030	1854	1929	1947	874	1465	1653	2515	2498	1109	825	1551	2000	1347	2037	1090	905	1294	1555	1202	1737	959	714	1685
609	UL16-binding protein 1	ULBP1	2088	2020	2388	2331	3189	3041	3660	5376	2891	2626	1392	3524	2514	8710	1742	85995	31351	54510	1117	76593	149072	33207	55851	86619	80999	135158	81430	200098	127233	36621
610	Uncharacterized protein C3orf20	C3orf20	4112	5050	5075	4219	6136	6583	5364	3602	8301	7868	4147	3904	3745	4775	6171	1990	3314	1817	2660	3062	3112	3883	3472	4739	3557	1200	3841	2398	1481	2313
611	Uncharacterized protein C9orf153	C9orf153	76	74	54	66	111	38	45	40			20	16	16			215	177	226	74	83	178	119	243	42	49	71	33	17	74	172
612	Unconventional myosin-Vb	MYO5B	5927	14849	11929	10626	17991	15863	19021	13557	11056	18474	21483	14306	19043	6665	17655	8639	8090	8702	14168	12786	9674	12099	11303	17411	12771	18118	21322	14952	21367	5350
613	Unconventional myosin-XV	MYO15A	11564	14397	14421	13518	12748	16972	18872	20391	29945	20789	28130	27213	23726	18992	19333	7462	10064	8224	5768	11372	15536	12095	14209	22399	16345	13724	11562	15258	11646	5201
614	Uracil-DNA glycosylase	UNG	122	138	85	62	116	65	82	38	205	27	315	22	34		49	21	35	4		20	70	118	71	58	64		16			34
615	USP6 N-terminal-like protein	USP6NL	1794	3062	2820	1372	1789	3508	2780	2468	2393	2453	2071	2918	1958	2094	2448	1148	1380	2004	3640	3365	1472	2132	1532	1929	1814	2404	2238	2585	4044	2089
616	Vacuolar protein sorting-associated protein 13D	VPS13D	13272	15284	9421	13229	17677	18276	18020	17092	13920	17715	21296	16145	19769	19534	25513	9175	8219	8858	11372	11947	11840	10662	11645	11740	13241	15058	15140	16024	12617	5542
617	Vascular cell adhesion protein 1	VCAM1	4575	8882	4701	5433	5992	4893	7009	10735	4791	8870	7829	6560	5599	7171	4386	36046	15664	27801	30147	25932	44257	17441	37348	19677	31718	40751	20745	26732	23061	8519
618	Vasopressin V2 receptor	AVPR2	1165	1134	933	1501	1784	2165	1310	2130	2607	2831	3025	3326	2128	2944	4321	439	670	1450	1673	2276	925	2314	1124	1918	2247	1617	3055	1727	5842	576
619	Vasorin	VASN	208	499	679	912	389	180	628	291	1334	532	687	403		25	74	1057	632	2133	1144	696	1112	1083	1064	12937	798	1300	578	1219	1377	466
620	Villin-like protein	VILL	24836	33005	28392	27065	27329	36442	41588	44370	63541	43640	58989	55659	48243	39188	40109	25566	19479	15875	12322	23506	33155	24294	31136	48103	30716	29358	20894	29173	24692	9715
621	Vitamin D-binding protein	GC	684265	917224	730364	764941	915104	1114859	1083377	1131223	1621966	1473953	1443442	1007960	1334367	1299199	1465987	368872	463533	550258	343295	790460	992285	779565	851721	1308287	993300	1051027	902010	990501	854217	280926
622	Vitamin K-dependent protein C	PROC	1001	332	323	457	533	604	319	166	437	453	425	194	271	139	207	28	130	16		6	13	87	18	25	32		82		9	344
623	Vitamin K-dependent protein S	PROS1	18849	26830	15003	26771	18191	28857	23932	22391	26291	31471	35660	24294	15816	23312	42819	15004	21271	32578	33567	12546	19771	18399	24021	35186	20545	33549	24145	54252	43694	9997
624	Vitronectin	VTN	301908	403847	350826	303804	277472	450358	427456	424296	720055	509109	573385	559039	671796	544685	656194	88061	225084	123437	236211	266182	193666	346644	276609	460393	328218	283615	540060	508400	348344	162495
625	Voltage-dependent calcium channel subunit alpha-2/delta-3	CACNA2D3	675	693	1052	996	693	1229	1080	1065	1298	979	1114	815	751	914	769	1542	828	671	716	643	667	1131	957	584	419	725	559	628	912	407
626	von Willebrand factor	VWF	3632	6574	6923	3552	5302	3626	4081	4071	5036	4400	5181	3478	5267	1453	5474	10890	10095	43059	75357	11599	17625	8640	16944	17586	22536	21154	11441	32587	37759	7857
627	V-type proton ATPase subunit E 1	ATP6V1E1	7874	8445	8848	8972	8643	8316	9316	9178	12400	11287	8311	4196	9027	5102	8178	6090	8223	6184	4885	5686	8866	14242	9464	8785	9974	5329	18698	9361	10127	4182
628	WASH complex subunit 4	WASHC4	8	13	46	21	19	1	13	5	2		66	8	4				115	11	9	23	13	33	41	12	99	10	23	1	9	74
629	WD repeat-containing protein 31	WDR31	710	1667	466	735	1060	960	810	1124	594	1203	858	866	1037	856	644	251	270	1137	2547	1434	878	587	378	1348	923	1635	1083	1141	505	867
630	WD repeat-containing protein 44	WDR44	12436	25384	8163	12562	21447	23918	22212	33421	14939	36246	28790	34170	32621	38442	35368	9176	12113	31015	69920	42734	30219	20062	29611	39051	42557	69470	48992	57525	40429	20332
631	WD repeat-containing protein 48	WDR48	4950	6322	4191	5727	5917	8021	7409	6995	9283	7265	7455	7995	4664	8258	9953	6845	5458	8405	9311	6940	5684	5234	6930	8317	5012	9849	6308	14452	10791	3090
632	WD repeat-containing protein 53	WDR53	535	315	246	489	1086	241	449	338	172	124	446	424	309	10	207	614	242	142	14	102	150	333	206	98	142	35	24	89		191
633	WD repeat-containing protein 87	WDR87	2867	2951	2609	2843	4238	3401	4167	4388	3149	2534	5240	3953	3885	2205	4222	3255	1996	1752	1382	1989	4529	2788	3052	4089	3082	2820	2497	3017	2360	2128
634	WW domain-containing transcription regulator protein 1	WWTR1																199	80	22	22		439	32				22	16	1377	983	113
635	Xaa-Pro dipeptidase	PEPD	125	122	70	127	176	94	82	115	56	93	151	92	100	51	131	65	41	154	835	105	106	42	44	174	108	234	49	113	160	54
636	Zinc finger CCCH domain-containing protein 15	ZC3H15	2561	6369	2388	2584	3201	4153	4885	12478	1883	10488	3550	2917	3311	6540	618	1220	6203	8834	11197	11876	13780	10028	10096	15993	13028	19880	15430	12772	7558	5157
637	Zinc finger homeobox protein 3	ZFHX3	366	150	163	521	428	471	284	305	386	508	411	592	132	182	563	162	77	302	120	318	227	539	149	217	338	120	335	99	799	150
638	Zinc finger protein 211	ZNF211	11766	13713	13986	20997	31607	26612	14268	16954	18030	30230	35022	17863	44132	15275	38624	8631	6387	6433	717	7198	15248	14090	18530	27366	32944	5614	27530	16299	15394	12588
639	Zinc finger protein 300	ZNF300	5609	9723	4672	7165	8363	7390	8069	10721	5751	14691	9418	9839	9726	10736	7749	7449	6945	18297	26765	19540	17716	10910	16177	18351	17696	27258	16511	21695	19681	5666
640	Zinc finger protein 34	ZNF34	5577	8777	10184	6154	9768	7019	10353	9985	8051	9445	10040	7889	9354	8777	12873	2711	9137	5623	4374	8543	9536	10164	11767	6946	9840	6234	11792	12374	12248	4359
641	Zinc finger protein 484	ZNF484	4119	5191	3800	4990	5086	4840	4531	6453	7392	5840	7704	6100	4724	3538	5947	3440	2969	5696	5023	5579	5605	4523	6412	11407	6249	9238	5496	8946	10964	2898
642	Zinc finger protein 532	ZNF532	52	57	25	7	66	72	99	69	103	18	42	21	8	2	4	5	12	8	24	24	15	22	20	8	36	50	5	29	10	48
643	Zinc finger protein 844	ZNF844	243	230	368	353	227	222	300	146	158	107	118	86	164	15	277	161	168	145	108	132	110	140	169	111	183	284	253	146	56	198
644	Zinc finger protein neuro-d4	DPF1	2944	2450	2229	4161	2012	4073	2817	3914	3999	3544	2849	4598	2433	6976	3343	2196	1895	1793	2501	2492	1792	2877	1512	2911	1980	2029	2798	3525	2301	1520
645	Zinc-alpha-2-glycoprotein	AZGP1	61295	89708	36076	69124	84805	56863	51588	60264	49572	75712	157937	109249	90350	62310	107842	69196	48321	74281	61215	44328	82377	66893	89000	96174	89837	97021	63103	86803	99083	47685
646	Zygote arrest protein 1	ZAR1	7474	9840	6220	6871	10215	8422	9071	10314	9428	14391	11511	14005	8371	11150	6645	3552	4898	4058	4532	5675	5435	7627	4420	11600	6460	7177	4671	6411	2249	3837

	Group→	iCCA															CIR														
	Sample→	iCCA01	iCCA02	iCCA03	iCCA04	iCCA05	iCCA06	iCCA07	iCCA08	iCCA09	iCCA10	iCCA11	iCCA12	iCCA13	iCCA14	iCCA15	CIR01	CIR02	CIR03	CIR04	CIR05	CIR06	CIR07	CIR08	CIR09	CIR10	CIR11	CIR12	CIR13	CIR14	CIR15
No.	Gene ↓																														
533	SCRN2	63	193	113	123	156	33	43	130	7	57			22	93		1583	1027	508	33	21	703	160	461	76		210	26	12	13	67
534	SELENOP	3133	2895	1737	2599	2279	2350	3602	4549	1904	3610	2521	1000	4178	1591	3080	3604	3710	2853	1402	3135	4905	5168	2514	1773	3825	4677	2757	5897	3195	1243
535	SEPTIN6	2995	4941	4577	3666	3972	4864	7124	4732	6952	4557	6158	6449	7501	3574	3512	2723	2517	1937	3159	4974	4071	3653	6925	4978	6317	4832	5366	6800	3710	1541
536	CDC42BPB	1716	704	266	1247	2675	1772	2106	4255	1451	1653	2292	1305	2928	300	2781	2705	2032	1878	1569	2713	3431	3015	403	2909	1485	5220	1650	1595	4699	824
537	TAOK1	585	623	473	1427	587	1004	307	701	1029	1090	800	859	1328	3335	1490	1862	2124	1791	677	937	2264	776	1893	1103	1735	1930	1558	3199	1472	789
538	TAOK3	46	293	88	233	87	318	267	81	117	68	51	60	957	137	283	207	315	978	26	138	415	96	617	197	106	209	158	426	2619	496
539	ERN1	177	524	128	299	452	384	519	10	471	535	203	1319	1408	18	1449	102	706	399	217	436	1681	357	918	1325	1189	535	1443	1376	495	1009
540	STRAP	1404	1400	3057	4844	6129	1232	2448	2717	4701	4005	2288	10463	2067	1282	9004	3327	5089	11349	7729	3054	5293	9244	2665	5072	4173	5990	6750	2957	7848	3818
541	TF	57712	56755	92559	125839	107676	51352	48218	116580	113589	127849	71816	132790	60724	63076	175998	153943	159744	192586	142998	76436	120356	131946	93341	119793	110472	174149	130546	83318	152165	88645
542	SERPINB3	1546	1753	3853	2172	2901	2801	3072	2879	3529	4268	6355	5055	1850	2472	3045	1065	740	1510	3797	2537	1636	2101	2216	4184	4101	2086	3303	1775	3901	1095
543	SERPINB9	2382	1612	4633	7446	6986	2002	3325	1059	6652	6242	3940	9964	1375	3982	12674	2765	2819	11369	8933	3757	3913	10879	1847	7173	6497	5831	7481	2398	10384	4345
544	SAA1	7749	44510	15492	6646	6587	15014	5653	27037	26027	14626	16711	9886	8177	91682	6974	2829	3667	2615	4259	9932	20316	5447	131427	5803	10161	10473	82549	10047	6143	4275
545	SAA2	110	1142	1787	221	235	88	44	16	716	56	139	172	69	2008	62	221	243	509	213	189	1787	231	37702	49	65	104	1185	161	92	231
546	SAA4	18538	22700	29103	21830	20991	32847	24701	31768	33478	30648	38151	24100	16732	18108	24055	17230	18646	14131	18305	40409	28776	27201	30063	17148	31488	31478	43497	29431	26609	15559
547	APCS	43216	36616	39369	41265	51995	56791	54434	80214	71736	64070	109885	81873	69416	44469	77719	20779	25418	20401	59821	65806	38064	35101	58534	58631	70735	52833	55931	60519	81868	21687
548	PON1	61381	33944	70242	79319	61119	39463	48869	130090	60556	56187	97613	36649	34765	19299	157997	39873	103750	72941	46099	93205	64632	73289	38667	33930	29773	121101	64448	27936	180342	34573
549	SHBG	9379	12507	10469	21147	25977	20943	18803	19618	20761	18168	18836	30636	15455	17288	43222	18615	12917	22641	7803	7772	12127	17259	21654	17010	20197	18508	19833	22115	19234	15947
550	SH2D6	281	532	690	801	1043	227	655	313	951	544	371	1734	540	330	1216	779	874	1295	1336	309	723	1031	524	1129	1287	944	1405	665	723	496
551	SIGLEC9	15001	10446	16835	27651	31160	17968	4180	30759	24164	33184	21585	13483	5422	14370	55580	17045	17316	43829	31847	33147	30265	41062	21521	19579	31793	40589	26858	27603	63179	14567
552	SIPA1L1	27142	35162	27086	38857	38265	41963	35140	48726	58534	52990	57401	46182	63332	65668	50306	38929	39858	28680	34632	33845	51529	41827	54490	57855	66468	62705	45789	75527	48256	17975
553	SGSM1	257	299	330	174	376	217	49	231	132	455	421	36	90		51	27	11	85	319	392	45	317	93	255	38	399	27		269	55
554	SMPX	2163	3884	3435	3002	3846	5316	7720	4889	5576	4485	4331	8172	5152	9675	4349	2839	2455	2685	2024	4616	3312	3692	5938	4138	5800	3588	6175	6299	4695	1164
555	SNX4	3677	2755	4189	3212	2758	3152	3824	3139	2372	3540	3776	2368	3027	1662	2522	2446	2819	2615	4344	4527	2765	5925	4188	2769	2790	2381	2634	2661	3645	1626
556	SPTBN2	1118	1391	1942	2516	2695	1230	1938	2246	2338	2099	1249	3647	2043	3134	3832	2069	2748	3986	2883	1174	2374	3192	1955	2636	1489	2417	2399	1862	3241	2359
557	SPTBN5	3007	613	1371	790	2003	828	1600	4894	359	2803	2306	257	475	25	1640	1304	1453	769	2348	5693	1572	2507	876	1577	1267	3600	132	1304	3384	1193
558	SPEF2	19484	22849	43740	35877	37132	25167	23235	52133	52097	55722	50703	59788	30547	19674	55107	19864	20850	46352	46989	28126	23825	33305	39179	48884	44618	47520	43883	29518	47796	12752
559	SPAG5	3013	5138	4219	4083	4429	5459	12791	2801	9499	6027	10583	18821	13644	8869	6134	5062	4895	3477	3632	3189	6318	5707	5713	7933	11093	5599	8422	7218	6976	3036
560	SRM	8769	4178	6445	6218	9579	7086	11632	15746	7010	7600	9516	6309	1301	3404	5955	8053	3776	11285	5614	10765	6812	12065	879	8262	5456	11234	7711	1925	11844	2351
561	SASS6	2336	974	1448	1966	3281	2319	2537	3786	1424	80	9469	1319	439	121	928	666	287	386	221	790	776	1278	462	1358	3240	282	5212	756	54	387
562	FDFT1	5531	2515	2168	2286	3494	2613	3374	6809	1409	3231	2338	2596	4116	1113	2991	6088	6230	2515	2660	9105	6007	4500	4541	3495	2396	7275	1238	3993	4955	2549
563	SMC1B	1036	835	895	1151	1062	1254	2350	1094	2228	1761	1550	1901	2145	158	1925	765	953	1260	1038	1075	970	1239	658	1921	835	1300	2198	844	2071	730
564	SDHB	1166	1413	1775	2682	3760	1429	1298	2505	1960	2398	1068	2566	2202	646	5641	1783	2457	3921	2871	1242	2626	3644	2094	2826	1241	5062	2285	1978	3056	1400
565	SOD1	25	19	34	20	34	4		1706		16		29	94	10056	12	10	175	149	68	4	34	42	1723	10	10	13	29	368	12	29
566	SOD2	601	2067	20	1880	365	489	482	610	509	1004	37	3149	1636	23656	3454	3973	10922	3175	124	181	2339	1885	783	263	2641	1539	9212	7630	1720	1169
567	SYMPK	33581	34696	64282	96062	125029	47806	63985	111295	101356	104744	59495	155960	89684	100343	235118	66853	98776	163200	114221	52509	111862	158629	84988	117387	90564	143233	131044	109361	185581	50858
568	TRBV24-1	3115	3583	7793	10569	11055	4626	7234	11570	10501	12552	5694	4909	7081	2697	15212	7484	9055	18356	7580	5458	17001	19077	6026	15012	9095	15532	4874	8256	12302	3623
569	TRBV7-6	164	1313	208	91	28	594	1684	74	431	155	188	41	3424	456	29	1027	698	162	27	16	960	188	441	149	350	51	624	773	21	105
570	TCP11X1	1263	1328	2504	2294	1468	2201	3945	17	5367	2694	6583	8752	4782	2221	5135	719	1681	2174	2840	2109	3536	1960	1769	3665	7327	1685	5871	6903	5202	1910
571	TCP11X2	232	172	225	275	246	266	160	263	265	587	848	272	176	1	194	67	15	26	160	364	99	71	197	475	321	348	227	100	187	99
572	TNXB	400	1184	316	774	327	1608	1676	402	1042	486	664	1129	5093	2553	663	2035	2319	729	267	164	2315	542	1846	843	1205	600	2230	3086	672	588
573	TEX52	37	3	17	44	94	137	5	40	33	135		269	16		116	4	2	128	4	12	54	22		330	395		239	113	39	311
574	TEX54																														
575	ASPSCR1	9988	23836	27366	32787	25472	20549	35215	36147	34536	32425	20359	33613	30268	30770	42383	28386	42519	48364	19517	18366	49224	47287	35010	30983	25121	44421	40959	29142	43994	9357
576	CLEC3B	9006	8422	6466	14285	8975	10939	14601	12355	12507	10404	10262	13958	16708	19414	22626	13835	17821	17178	10868	9147	17065	17472	12700	11136	13852	14523	30103	17678	21238	9647
577	TTC21B	225	309	176	183	93	88	58	8	52	59	104		166			216	249	24	134	66	121	20	146	152	180	107	9	22		15
578	TXNDC15	109	246	357	170	178	282	402	67	367	259	78	363	231	266	44	261	252	202	80	158	233	226	709	226	268	124	464	315	78	82
579	TXNL1	9279	5955	5179	8541	11687	10632	9024	14870	13140	14819	19511	16636	9936	4808	13568	5310	4006	6923	11665	15065	7107	12989	8420	12038	9685	12588	9118	10485	10957	2968
580	THSD4	114	202	96	65	86	54	44	42	52	64	20	156	418	23	9	227	271	98	26	100	53	56	99	52	55	100	62	132	6	192
581	THBS1	33771	34230	42937	31737	43685	47131	50301	55013	56955	53197	81276	70501	46007	53610	52500	24735	26425	37180	48602	40065	40855	38493	49712	48025	68353	41672	58972	58853	68676	24799
582	TYMS																														
583	SERPINA7	25794	33200	31565	42748	47426	48998	45507	61293	44687	48292	55701	77235	63316	64641	66483	39434	40816	38976	34793	33061	44449	61214	52997	52225	47832	77095	75544	54913	59136	30663
584	TIGD4	3998	4082	5284	9567	9594	4587	5008	7761	11413	8959	5802	15510	7893	6896	14907	6831	8390	13097	10237	4837	9518	10930	8985	11850	10629	10732	12094	11578	13037	6067
585	TTN	54611	50218	62895	84525	89084	82348	77433	97595	110302	100889	118866	153136	100001	91108	163336	69545	80342	114235	87756	79480	104334	108999	88330	101738	155788	93181	139548	134953	170980	65935
586	TOR1A	1665	1233	1073	1632	1483	1687	2595	1746	3779	2237	2293	2506	1959	1001	2520	774	607	1781	1382	1375	805	1545	591	2614	933	2163	3066	856	2433	892
587	VGLL1	505	42	111	32	357	14	66	383	14	171	720	281	30		17	111	109	74	377	881	37	404	101	42	55	65	49	25	632	702
588	TCF7L2	9492	9758	15311	15346	16605	18044	22506	170086	25792	19906	27937	52909	20432	177766	28027	7867	8301	14413	13182	17725	8763	13572	14360	22213	31825	13885	33200	22778	25378	7678
589	ETV7	1795	1020	1623	2371	2781	2064	434	2543	1657	2579	1316	691	481	224	3166	2563	2562	3749	2527	3344	3292	3493	2028	1486	2518	2694	1580	2421	4195	1730
590	TADA2A	2444	1357	1451	3084	2339	2242	2808	3308	1607	2641	2511	4476	2933	2956	5470	3507	4106	4512	2486	1951	3971	3742	2920	2547	2673	3103	2130	3353	5524	1741
591	TGFBI	206	505	1010	649	1367	466	1248	451	456	368	303	1099	993	2328	1926	476	699	1691	793	722	496	2276	314	181	247	834	655	169	1732	576
592	TRPM1	2390	4923	4054	4076	2806	5496	8143	3397	6427	4538	6335	4559	12571	27393	4484	5586	7674	3758	2815	2647	7765	4178	8473	4062	6778	2853	10962	10035	5138	2250
593	TRPM6	320	150	211	107	488	160	348	17382	224	293	29	1831	932	56798	80	225	767	319	41	105	134	263	663	139	244	47	509	110	91	244
594	TMDD1	722	429	806	653	715	718	1232	44	1153	1103	1257	1438	301	337	432	173	81	616	627	631	122	585	121	1160	291	560	1084	120	694	538
595	TTR	333981	92707	246665	195523	336634	133799	255677	589470	192174	495277	492605	356651	154628	204922	461020	135241	156137	241703	445957	635260	193332	433251	150300	294831	389904	420368	211927	252109	709244	246720
596	TNRC18	283	301	344	139	375	225	551	91	265	336	387	696	119	597	114	130	67	54	290	61	73	255	38	174	383	60	434	13	82	273
597	TNRC6A	1024	1613	1492	2989	3614	1197	985	1753	1119	2578	444	123	2702	10	3998	3893	3696	4965	2315	1625	4405	4649	4041	3092	467	4252	187	912	1825	2564
598	METTL6	720	566	1718	764	760	1093	593	1937	1118	929	3167	669	619	714	342	215	117	197	1148	1332	322	257	4254	914	1510	444	931	748	540	479
599	PRSS2	2994	2954	5777	2523	3169	4506	3984	6932	4919	4825	9363	6068	5962	254	3202	1776	1307	1358	4214	4987	2771	374	6597	5328	5707	3129	3521	5994	3273	1407
600	TSC22D3	2955	5344	3571	4280	5753	5401	3409	16621	3750	5852	3564	3694	11910	11256	4947	3654	4542	2420	2878	7008	4957	3574	7528	5478	4362	7379	2736	5249	4070	1290
601	TSNARE1	65566	76160	67662	83987	74583	96443	41963	112187	134726	127784	144096	72086	59745	65848	132929	58188	78058	54782	90787	84271	109494	106718	91613	115272	147260	104758	130503	120939	152232	49614
602	TP63	5312	5297	5134	6133	5641	6643	8041	8021	9471	8293	8376	6691	10476	6240	9058	4254	4064	4793	6650	6048	5340	6585	3909	8789	6378	6472	8524	4785	9642	3171
603	TWNK	217	336	128	1876	221	60	13	24	533	123	109	223	112	17	4008	462	259	2155	26	309	28	10	3135	257	5424	276	1453	6709	674	266
604	TEC	143	2074	341	6971	1513	706	591	2270	2468	1668	1118	5227	1697	19183	19585	1197	2287	8793	483	336	1463	228	1148	2395	23850	1574	9296	31681	5045	1876
605	UBE4B	31655	36168	33161	34742	39483	41102	33544	52402	41129	52843	44570	38951	52814	30605	49672	38090	38599	33207	36208	42587	48934	42015	47027	45502	45055	52105	46031	50330	55823	20615
606	UBD	3353	2646	7035	2937	3401	4708	4429	10423	5020	5748	8166	4635	2729	4211	3892	1926	1309	1871	5548	6349	2786	868	5680	4643	6125	3105	3642	4093	4074	2037
607	UBTD1	557	44	635	275	529	482	26	488	329	499	728	66	2533	102	447	52	103	280	364	1248	0	932	352	214	67	184	213	43	742	144
608	USP18	989	863	1198	973	1206	733	1096	629	1204	1703	1239	849	633	173	758	1092	1437	2061	1382	561	1500	1867	549	870	529	1039	862	442	1614	1495
609	ULBP1	22237	23036	29782	63336	59251	52178	35431	18300	52531	47590	34690	121328	47519	88026	183835	52398	84483	111546	44476	26178	91814	92702	86772	100288	60100	67081	138106	131334	202167	57168
610	C3orf20	1956	3168	3697	3885	3354	3872	5101	2058	3736	3664	6492	3831	3683	180	2622	2075	2024	2492	3184	2850	4433	2652	3889	4305	4967	3032	2796	3286	3077	2131
611	C9orf153	137	168	112	188	153	269	123	108	27	53	41	209	315	76	122	218	243	92	48	150	204	62	282	120	90	8	44	171	14	171
612	MYO5B	5837	8553	9529	14367	13961	12245	13170	12239	17897	14605	27182	21271	11119	23996	13034	7191	10849	9840	9887	12849	10460	12589	13551	16729	24545	12464	31296	23807	14142	4494
613	MYO15A	10156	5263	9006	13272	16392	10769	3401	14586	10981	15690	10865	5223	4753	4670	23223	13947	13573	19615	12417	17771	18712	15839	14301	9193	16340	19409	11238	16504	27910	7037
614	UNG	105	63	17	64	23	35		102	77	164	43				7	17	26	12	99	115	62	30	1	39	47	44	16	8	71	71
615	USP6NL	1836	1721	2657	2153	1713	1352	3289	5318	1459	2741	2852	2686	569	8258	2655	1522	1179	2313	2090	1623	1360	1985	1304	2056	2450	911	3252	1142	2533	2275
616	VPS13D	8353	7183	8604	7493	9897	11383	15671	13555	12272	13738	16532	15732	25289	8314	11530	8447	9017	6805	10278	12541	9615	9997	8918	13146	14027	10459	11389	18494	15539	5155
617	VCAM1	8508	20568	13641	16781	24539	10960	12961	15418	15478	16927	6299	10753	18555	12853	25861	31947	33152	31020	13105	10133	37150	39542	32824	21823	11060	45961	21134	13847	21061	8777
618	AVPR2	1343	912	1282	1460	1547	1344	2199	2599	2903	2365	3051	2672	1309	2103	2669	690	390	1738	1265	1567	666	1653	549	2445	1479	1849	2592	874	2538	757
619	VASN	420	591	542	876	663	666	322	711	426	883	132	63	308	304	1436	779	794	744	580	713	624	728	535	1439	1636	334		2049	1213	341
620	VILL	18269	9848	15590	26961	34544	22485	6740	27463	21673	31451	20169	11892	9106	12539	46159	30050	27696	41773	25265	36031	42023	38920	28964	18288	35290	37330	23739	33989	55013	13442
621	GC	424919	563323	533204	586457	679580	777665	749946	1009466	974104	1064632	1208142	1015928	851977	825364	926877	483803	532859	463292	495432	608477	785003	867656	846106	886603	888606	861040	1040865	871580	1022451	276846
622	PROC	499	75	24	35	137	349	98	133	87	60	318	365	219		12	76	50	12	351	586	29	27	15	7	69	26	15	10	29	234
623	PROS1	14506	14395	10646	17376	22836	17165	12715	24193	25949	28010	23889	21022	26455	50952	18977	14124	17326	16266	22771	18903	27832	19992	29660	23374	39075	18470	36592	37644	33054	10786
624	VTN	198137	165462	244861	218691	282259	270884	282646	577105	439117	412742	498790	511579	352761	324050	420086	149114	128075	197062	338884	305060	211611	279062	305296	441108	450761	361665	373920	317886	484385	169014
625	CACNA2D3	680	759	816	751	815	850	1043	1276	928	1084	896	943	994	2848	602	660	799	532	833	650	544	642	711	199	721	758	725	1298	759	454
626	VWF	4695	14206	7488	19211	13763	12460	20362	10841	10637	9711	5587	13063	15833	63543	32437	11664	17183	13710	6967	11212	17612	7680	31605	14016	35026	6597	89735	40757	8711	12890
627	ATP6V1E1	6985	7606	16918	6723	8510	12191	8457	15458	14998	15598	21638	13836	16991	401	9010	5446	4386	4118	13680	12367	9614	1108	21458	14527	14360	9473	9645	18154	9313	3251
628	WASHC4	7	20	39	74	270	59	3	21	8	46	30	12	11	467	17	119	28	32	56	37	12	31	6	54	17	94	3	15	48	60
629	WDR31	372	188	496	987	695	292	365	104	679	475	382	1098	155	96	992	218	370	1687	1213	647	638	927	404	705	1057	387	894	510	874	679
630	WDR44	7959	10380	15344	24147	31242	10535	15789	27740	29580	25000	16348	45884	15282	25490	56486	13591	15995	37821	28178	12594	20595	37695	16072	34519	27455	38191	36127	21191	43573	17697
631	WDR48	4075	4569	4598	4615	6054	5752	6855	5287	6855	5706	5682	5725	6970	6779	5129	4449	3960	5600	6972	4524	8629	6494	7422	6500	9227	6599	10149	8682	9256	3736
632	WDR53	351	70	59	71	146	22	109	25		104	15	50	36		51	402	411	107	137	667	319	268	80	74	50	374		42	131	164
633	WDR87	2383	2085	2500	2509	2751	3209	2022	4642	2891	2865	3290	2729	2466	1674	4141	2730	3104	3109	2533	4170	5047	2706	4058	2723	3997	3785	3398	6426	4659	3139
634	WWTR1																60	127	31			32	34	937	136			238	821	670	213
635	PEPD	45	22	48	68	51	81	65	93	57	40	45	226	40	1904	163	65	97	91	66	72	139	55	59	45	44	37	386	100	80	91
636	ZC3H15	1506	4508	8829	12086	11622	3869	5849	9170	11090	9485	2558	9571	1682	2269	15214	6742	6201	16830	6193	2657	10397	17933	5048	11720	3123	16872	9065	2899	14513	4767
637	ZFHX3	183	229	104	157	180	241	1002	96	703	352	484	267	432	509	201	195	112	232	173	112	146	234	37	408	42	168	586	33	263	267
638	ZNF211	9152	3466	6426	19891	8766	8426	4233	17845	8185	19488	11326	410	4526	4271	35938	11616	17740	11262	11447	5491	24399	11055	15989	18557	14348	8427	11468	12566	19629	11306
639	ZNF300	5233	7612	9456	11673	13236	6968	12415	11982	15226	12656	6930	17857	13041	7415	21322	9770	15683	18269	12228	6663	14442	16870	11071	14702	10615	15863	13100	11332	17657	4970
640	ZNF34	6480	10497	10200	8254	9691	11050	16540	11130	16404	10914	14993	15843	17667	12953	9547	6198	5905	5181	7179	12005	10886	8765	17158	11557	16609	12126	13746	18522	10496	3295
641	ZNF484	3244	3342	4059	5798	4974	4017	4250	7193	6216	6190	6396	4346	4485	3178	10323	3417	4181	7436	4491	4331	5423	5391	4134	6276	8986	6859	7441	10367	7097	3440
642	ZNF532	22	1	3	6	28	9	5	26	7	4	7	1	16	12	12	3	9	32	17	20	5	58	4	4	18	13	54	11	7	57
643	ZNF844	124	208	190	252	153	226	211	335	177	112	427	247	595		80	112	79	81	191	125	136	88	67	265	308	264	260	280	136	172
644	DPF1	1681	1283	2385	1737	2589	1614	1787	2753	1561	2263	2847	5751	3707	1726	2148	1313	2160	2134	3313	1640	2500	2659	1512	2391	2579	1743	2160	2222	2844	1611
645	AZGP1	50507	55835	46995	82146	50373	82960	124718	159082	86779	69001	87031	98384	156438	192165	98888	53756	88394	53074	42667	102165	68571	59112	116821	62081	108024	66205	103925	136064	72659	43318
646	ZAR1	3193	3222	3334	4256	5391	3586	2178	4057	3233	6436	3700	2569	1482	4612	7408	4744	3697	11126	5790	4459	5556	8967	2728	5307	7724	6526	3219	5615	8613	3610

	Group→	PSC														
	Sample→	PSC01	PSC02	PSC03	PSC04	PSC05	PSC06	PSC07	PSC08	PSC09	PSC10	PSC11	PSC12	PSC13	PSC14	PSC15
No.	Gene ↓															
533	SCRN2	109	269	43	41	19	37	25	25	49	15	15	38	28	12	59
534	SELENOP	3026	3009	4097	2581	2761	5729	2886	3455	1925	2851	3966	4184	3486	1571	2272
535	SEPTIN6	4164	3938	3932	4365	4512	3948	4237	3426	7448	5416	5824	4420	2532	6036	2084
536	CDC42BPB	2033	2496	3186	3370	2275	4153	3805	2967	2994	3979	6436	5539	6164	7544	757
537	TAOK1	492	1544	1947	578	1584	772	860	1868	1033	1848	1038	1468	2137	1501	404
538	TAOK3	97	156	86	74	255	88	86	86	92	87	55	56	711	42	581
539	ERN1	73	92	164	206	1733	289	162	208	548	128	247	1347	2670	524	284
540	STRAP	1307	2373	3193	3153	6822	5403	3467	2505	4747	3542	1987	4788	8305	6056	1078
541	TF	51375	96585	103878	94929	87551	91903	163505	102494	120466	122956	78684	203792	170533	157823	39920
542	SERPINB3	1498	1779	1555	2906	2165	2142	3618	3852	6496	4009	5640	4560	2782	4754	1268
543	SERPINB9	2223	3224	4985	4983	7571	6935	5169	3608	7308	5961	3447	5405	10454	7542	1511
544	SAA1	5166	21954	4693	5267	7829	5053	6074	10113	47333	7022	8003	22277	5218	8228	5197
545	SAA2	103	552	117	86	148	107	46	259	542	34	27	29	109	44	85
546	SAA4	21801	31981	26602	23026	13070	24232	20480	24366	21422	33838	25083	25665	15642	40146	10610
547	APCS	34879	33772	28964	38636	39208	56015	50308	45963	87043	54589	83745	54042	46425	64505	43367
548	PON1	69779	84030	102705	74781	43216	100026	67171	99488	69761	110113	132603	111316	161502	158190	39769
549	SHBG	6651	8070	21162	11871	20933	6663	7179	11100	14858	20655	9906	24488	28679	23521	8805
550	SH2D6	373	369	395	598	1229	824	698	188	825	318	176	477	1086	840	356
551	SIGLEC9	16554	26355	38241	24507	14513	25057	30043	41533	31003	43049	35982	69319	70155	58540	6896
552	SIPA1L1	29663	32165	35958	38648	36981	38407	45560	54505	63387	70498	57036	64981	40714	61209	17796
553	SGSM1	369	296	95	352	223	235	386	416	345	439	469	60	34	358	116
554	SMPX	3023	3438	2494	3071	10623	3535	2807	4103	5851	5218	5009	4665	4824	4723	1330
555	SNX4	6558	5202	14356	6948	1950	7348	5045	5584	2720	3413	3776	1905	2027	2146	2914
556	SPTBN2	1208	1290	1815	1778	3275	2215	1568	1257	2101	1577	1030	2865	3176	2304	1001
557	SPTBN5	1344	1794	1792	1947	624	4021	3149	4043	1577	2760	3165	1441	1171	2178	1276
558	SPEF2	19463	22206	36950	33432	30861	30382	32543	45403	53708	46578	48526	52875	60810	50867	8711
559	SPAG5	4450	4476	4938	4260	11476	4918	5726	4962	8435	7875	6268	5371	4640	8444	3320
560	SRM	13180	11092	13932	12585	14017	14286	12338	8533	9931	10780	12195	16114	15443	13518	3489
561	SASS6	1587	2485	355	1466	409	1032	1180	536	2384	1887	1267	334	37	898	542
562	FDFT1	4507	6500	6646	4583	3621	8555	5197	7283	2265	4015	4930	4412	3901	4062	2406
563	SMC1B	1230	752	918	1840	1587	1360	1881	1559	1506	1514	1879	571	2470	1651	661
564	SDHB	1249	1165	1272	1628	2377	1985	2068	1784	1978	2429	1955	3958	3884	4605	393
565	SOD1															
566	SOD2	303	2199	599	455	2805	79	1394	156	979	4046	125	1572	8008	3237	430
567	SYMPK	28071	46241	60438	61598	127708	96631	75776	75482	94292	98537	57529	197077	205289	170264	15046
568	TRBV24-1	2267	4029	6989	5305	5591	12720	6438	4594	9347	8892	3740	18580	15601	8949	1076
569	TRBV7-6	542	335	193	134	218	186	179	71	96	242	42		8	27	129
570	TCP11X1	520	828	839	1347	5869	2427	2204	908	6720	2992	2329	1087	5214	4471	1818
571	TCP11X2	267	302	102	344	53	164	381	301	662	414	904	1033	44	566	255
572	TNXB	355	697	411	345	1610	630	414	393	662	675	322	470	675	656	586
573	TEX52															
574	TEX54															
575	ASPSCR1	17729	19881	25444	19204	38856	32364	22639	19563	28131	26421	17399	41909	39175	36111	6555
576	CLEC3B	9344	10927	18938	12308	22107	15613	14090	12946	14408	19818	15489	21380	23748	16134	6996
577	TTC21B	186	249	141	133	31	61	79	120	40	22	160	15	24	8	23
578	TXNDC15	111	187	142	177	309	58	93	78	192	258	118	17	43	91	93
579	TXNL1	6714	8922	9658	10896	7979	11501	9304	19599	14103	14755	22480	6568	7317	19624	6683
580	THSD4	148	154	173	90	20	41	40	91	61	110	76	52	32	80	162
581	THBS1	30771	27806	38653	38403	44204	42259	46527	54393	63049	53109	72726	69706	63070	64364	27822
582	TYMS															
583	SERPINA7	26174	30180	33586	37285	61335	39668	36316	34362	68416	60921	42828	66010	44119	73755	17448
584	TIGD4	2892	4392	5532	5702	12421	7674	5883	6800	10152	8334	4704	12415	16387	10964	1905
585	TTN	43857	62528	66231	68757	107718	94534	85598	88628	117692	112852	79165	126032	161365	138251	49191
586	TOR1A	1819	910	1362	2791	2350	1579	2871	2265	1842	1910	2588	420	3352	2023	912
587	VGLL1	56	81	542	373	248	452	645	497	150	32	450	70	100	51	876
588	TCF7L2	8554	10997	12175	13083	42446	12465	12121	18308	32269	23662	24366	246179	31746	22757	8308
589	ETV7	1868	2368	4042	2335	1623	2441	2598	2989	2209	2947	2304	3945	4507	3564	1023
590	TADA2A	1986	3050	3507	3103	6650	3411	3033	3856	2673	5726	4872	6442	6368	4036	1385
591	TGFBI	368	510	309	589	785	682	425	425	707	567	239	724	1262	837	454
592	TRPM1	3131	3427	3046	3364	8299	3405	3311	2413	6264	4826	2826	8152	4111	5453	2619
593	TRPM6	86	309	208	56	3584	42	131	317	298	417	111	22277	131	236	210
594	TMDD1	1048	379	610	1838	663	547	1382	1010	978	1220	1799	16	1462	673	603
595	TTR	165627	241300	387733	340774	270029	560008	416901	515206	377591	416153	577744	445586	481357	423660	266323
596	TNRC18	321	124	250	394	814	36	381	131	415	245	374	332	36	351	289
597	TNRC6A	1204	1608	982	1038	959	2487	1383	1944	1011	2604	929	2726	4242	3220	437
598	METTL6	623	511	848	824	858	609	912	775	1334	919	1315	379	318	671	762
599	PRSS2	2672	1928	3903	3409	3438	3591	4282	5060	4836	3350	5527	2951	3816	3651	1966
600	TSC22D3	4774	4627	5221	4856	4445	3813	4580	5184	6483	8441	7504	10283	4276	8115	1674
601	TSNARE1	90975	76925	101587	99234	58961	115914	101430	102024	129490	157913	124836	140865	93829	130147	55051
602	TP63	4683	4626	6029	7368	6533	7669	7574	6478	8507	8106	7047	7181	9039	6941	3252
603	TWNK	222	275	191	561	246	7	88	172	353	797	29	392	1960	319	476
604	TEC	263	2302	1553	1597	7083	453	1195	660	2605	3907	379	2696	11156	2871	2310
605	UBE4B	37532	40096	44389	38929	34398	43114	44889	49867	46375	63333	55582	57843	48782	57465	16979
606	UBD	3315	2640	4469	4003	4070	4473	4847	5679	5683	3168	5108	6547	4623	4456	2418
607	UBTD1	976	708	1071	608	157	254	739	596	1145	694	1000	447	169	1113	211
608	USP18	914	940	1126	832	1206	1357	1352	1219	1097	854	1069	1549	785	1119	1417
609	ULBP1	13374	28181	20424	23142	149567	46483	34358	42289	55139	63007	26570	44222	143341	141504	13167
610	C3orf20	2177	2025	1653	2614	2737	4240	3520	2981	4490	2730	3495	1269	3101	3200	2429
611	C9orf153	106	97	38	69	105	45	62	62	42		51	9	26	35	152
612	MYO5B	6158	8845	7083	10235	18372	11161	9674	10313	15727	17414	13169	17098	12243	13482	3515
613	MYO15A	10467	14492	20584	11467	7676	12218	14159	19586	13550	19975	16202	22499	29056	26156	3996
614	UNG	45	84	27	114		117	108	143	59	122	90	16	16	17	69
615	USP6NL	1958	1737	1823	2258	3212	1436	2037	1729	3202	2254	2101	4883	3225	2297	1704
616	VPS13D	9630	9500	8918	10697	13555	14186	13640	13023	11777	14834	14430	10320	12266	14842	5945
617	VCAM1	11455	11515	9381	9998	16862	21215	9891	10841	13179	17093	6105	14461	21796	23764	3847
618	AVPR2	1785	830	1357	2878	2050	1529	2674	2442	2453	2562	3461	988	4249	2340	860
619	VASN	478	709	473	655	1052	775	897	935	632	734	747	691	1086	854	388
620	VILL	20038	30296	42359	22817	18072	24341	30424	41864	27322	41969	30451	46679	60854	52320	6943
621	GC	376567	555269	610201	538499	685862	723138	638161	754550	1080521	987627	944894	875345	744824	949011	369003
622	PROC	97	218	24	211	163	185	75	572	167	151	607	34		48	333
623	PROS1	15478	20620	17124	17780	42566	21983	19323	24068	24434	36725	33991	48130	29470	33211	10101
624	VTN	210463	234288	242074	272744	262852	293141	365296	321238	445678	372026	506889	671567	390710	613201	209697
625	CACNA2D3	876	792	697	841	994	340	909	687	679	1033	940	1017	338	510	697
626	VWF	3756	8135	4992	3855	24862	5649	5048	5418	14605	9454	6484	17231	24252	8215	6712
627	ATP6V1E1	4497	5008	7113	8175	9080	10321	11773	14042	15237	9551	13755	8141	10035	10253	4257
628	WASHC4	54	103	87	95	1	8	198	153	9	160	80	22	77	43	120
629	WDR31	266	256	486	550	895	821	623	344	725	398	293	244	980	572	302
630	WDR44	7033	10404	14672	15122	32462	26617	16366	13989	26163	21561	13369	41929	43048	36396	6446
631	WDR48	4472	5206	7025	5639	8989	6447	6972	7271	6698	8214	10181	11899	7200	9681	2735
632	WDR53	165	272	255	204	125	622	274	387	35	132	167		304	92	118
633	WDR87	1867	2414	3243	2500	2263	3193	3031	3423	2953	3016	3205	2509	4554	3909	1767
634	WWTR1	37	22		22	374	3		5		21		0	44	58	211
635	PEPD	34	57	30	82	379	63	47	41	44	118	84	290	133	32	52
636	ZC3H15	3447	4049	6135	3042	6660	8703	2532	2612	4630	5660	1250	12188	9533	11522	909
637	ZFHX3	167	72	20	384	343	272	527	139	189	225	234		453	235	253
638	ZNF211	4608	9359	11534	7191	4711	17206	15230	10662	20931	23376	22772	30281	11205	14538	7455
639	ZNF300	6102	6573	8089	8812	15943	12139	9203	8334	11924	10217	6931	13443	19558	14703	2288
640	ZNF34	9268	9232	9435	9722	10681	9231	10259	8994	17514	12901	14224	11508	8206	15914	4373
641	ZNF484	3442	3500	4444	4598	5090	4618	5016	5269	6515	7872	6168	8007	11993	6978	2670
642	ZNF532	23	17	52	29	18	12	32	35	21	19	4	14	21	8	42
643	ZNF844	139	113	96	156	180	110	224	123	241	196	362	208	101	175	165
644	DPF1	2271	2098	3611	2919	2509	2469	2906	2280	2906	2611	3178	4600	2177	2808	1397
645	AZGP1	43805	51841	52467	48116	117932	75379	56118	65826	62019	67297	88359	149410	97175	69632	41475
646	ZAR1	5045	4724	8097	4454	2255	5581	5612	6151	3041	7244	5341	4810	11895	9147	2598
