# Supplementary material for: Unveiling novel serum biomarkers in intrahepatic cholangiocarcinoma: a pilot proteomic exploration
Source: Front Pharmacol. 2024 Sep 2;15:1440985. doi: 10.3389/fphar.2024.1440985 (PMC11403330; doi:10.3389/fphar.2024.1440985)
Supplement: Supplementary file 3 [file Table3.pdf]

Supplementary Table S3. Overview on the statistical analysis
* = p < 0.05

			Tukey's HSD				iCCA-HC		HCC-HC		CIR-HC		PSC-HC		iCCA-HCC		HCC-iCCA		iCCA-CIR		iCCA-PSC		HCC-CIR		HCC-PSC		CIR-PSC	
No.	Protein name	Gene	f.value	p.value	minus L	FDR	p < 0.05	log2FC	p < 0.05	log2FC	p < 0.05	log2FC	p < 0.05	log2FC	p < 0.05	log2FC	p < 0.05	log2FC	p < 0.05	log2FC	p < 0.05	log2FC	p < 0.05	log2FC	p < 0.05	log2FC	p < 0.05	log2FC
1	39S ribosomal protein L22_ mitochondrial	MRPL22	4.3566	0.0033	2.4773	0.01	*	5.52		2.88		1.03		4.43		2.65		-2.65		4.49		1.10		1.84		-1.55		-3.40
2	3-hydroxyanthranilate 3_4-dioxygenase	HAAO	5.6590	0.0005	3.2788	0.00	*	-0.80	*	-0.97	*	-0.83		-0.57		0.18		-0.18		0.04		-0.23		-0.14		-0.41		-0.27
3	3-ketoacyl-CoA thiolase_ mitochondrial	ACAA2	4.1327	0.0046	2.3368	0.01		2.81	*	-2.38	*	-1.98		-1.54		5.18		-5.18		4.79		4.34		-0.40		-0.84		-0.44
4	Actin-related protein 3B	ACTR3B	5.7233	0.0005	3.3176	0.00	*	1.36		0.37		0.99		0.57	*	0.98	*	-0.98		0.36		0.79		-0.62		-0.20		0.43
5	Adaptin ear-binding coat-associated protein 2	NECAP2	10.0260	0.0000	5.7463	0.00		1.39	*	2.00	*	2.49	*	2.18		-0.61		0.61	*	-1.10		-0.79		-0.49		-0.18		0.31
6	Adenylate cyclase type 9	ADCY9	4.5856	0.0024	2.6201	0.01	*	0.57		0.10		0.23		0.32	*	0.47	*	-0.47		0.34		0.25		-0.13		-0.22		-0.10
7	Adhesion G-protein coupled receptor F2	ADGRF2	8.3716	0.0000	4.8517	0.00	*	-1.16		-0.32	*	-0.85	*	-0.85	*	-0.84	*	0.84		-0.31		-0.32		0.53		0.52		-0.01
8	A-kinase anchor protein 13	AKAP13	2.9998	0.0241	1.6180	0.05		0.25		-0.31		-0.01		0.05	*	0.56	*	-0.56		0.25		0.20		-0.31		-0.36		-0.06
9	A-kinase anchor protein 9	AKAP9	5.6724	0.0005	3.2869	0.00	*	1.10	*	1.11	*	1.03	*	1.34		-0.01		0.01		0.07		-0.24		0.09		-0.23		-0.32
10	Albumin	ALB	9.1791	0.0000	5.2943	0.00	*	-3.13	*	-3.01	*	-2.54	*	-1.49		-0.11		0.11		-0.59		-1.63		-0.48		-1.52		-1.04
11	Aldehyde dehydrogenase 1A1	ALDH1A1	14.9590	0.0000	8.1566	0.00	*	-0.93	*	-1.32	*	-1.30	*	-1.33		0.39		-0.39		0.37		0.40		-0.02		0.01		0.03
12	Alkaline phosphatase_ tissue-nonspecific isozyme	ALPL	9.0291	0.0000	5.2130	0.00		0.43	*	1.88	*	2.06	*	1.08	*	-1.45	*	1.45	*	-1.64		-0.66		-0.19		0.79		0.98
13	Alpha-1-antichymotrypsin	SERPINA3	3.3911	0.0136	1.8675	0.03		0.80		-0.01		0.48		-0.04	*	0.81	*	-0.81		0.32	*	0.84		-0.49		0.03		0.52
14	Alpha-2-antiplasmin	SERPINF2	6.8847	0.0001	4.0061	0.00	*	-0.53	*	-0.63	*	-0.59	*	-0.56		0.09		-0.09		0.05		0.03		-0.04		-0.07		-0.03
15	Alpha-2-HS-glycoprotein	AHSG	3.3203	0.0151	1.8224	0.03	*	-0.67		-0.03		-0.52		-0.04		-0.64		0.64		-0.15		-0.63		0.49		0.01		-0.48
16	Alpha-mannosidase 2	MAN2A1	4.6989	0.0020	2.6905	0.01		-0.48	*	-0.59	*	-0.67	*	-0.58		0.11		-0.11		0.19		0.10		0.07		-0.02		-0.09
17	Aminopeptidase Q	LVRN	9.0403	0.0000	5.2190	0.00	*	3.97	*	3.67	*	4.13		0.17		0.31		-0.31		-0.15	*	3.81		-0.46	*	3.50	*	3.96
18	Amphoterin-induced protein 2	AMIGO2	3.2027	0.0179	1.7475	0.04	*	1.54		0.48		0.68		0.85		1.05		-1.05		0.86		0.68		-0.19		-0.37		-0.18
19	Anamorsin	CIAPIN1	4.1087	0.0048	2.3217	0.01		0.81		0.09		0.13		0.80		0.73		-0.73		0.68		0.01		-0.04	*	-0.71		-0.67
20	Anaphase-promoting complex subunit 13	ANAPC13	10.8030	0.0000	6.1506	0.00	*	1.32	*	1.82	*	1.59	*	2.14		-0.50		0.50		-0.27		-0.82		0.23		-0.32		-0.55
21	Angiopoietin-1 receptor	TEK	13.1420	0.0000	7.3104	0.00	*	1.12	*	2.33	*	1.87	*	1.07	*	-1.20	*	1.20		-0.75		0.05		0.45	*	1.26		0.80
22	Angiotensinogen	AGT	3.4033	0.0133	1.8752	0.03		-0.46		-0.68		-0.49		-0.88		0.22		-0.22		0.03		0.42		-0.19		0.21		0.39
23	Annexin A9	ANXA9	9.7872	0.0000	5.6202	0.00	*	-1.04	*	-0.58		-0.09		-0.44		-0.46		0.46	*	-0.96	*	-0.60		-0.50		-0.15		0.35
24	Anoctamin-1	ANO1	13.6750	0.0000	7.5635	0.00	*	-2.71	*	-2.13	*	-1.81	*	-1.55		-0.58		0.58		-0.90		-1.16		-0.32		-0.58		-0.26
25	Anterior gradient protein 2 homolog	AGR2	14.6980	0.0000	8.0379	0.00	*	2.48	*	1.96	*	2.19	*	2.22		0.52		-0.52		0.29		0.26		-0.23		-0.26		-0.02
26	Antithrombin-III	SERPINC1	9.9539	0.0000	5.7084	0.00	*	-0.77	*	-0.83	*	-0.84	*	-0.58		0.07		-0.07		0.07		-0.18		0.01		-0.25		-0.26
27	Apolipoprotein A-I	APOA1	6.9139	0.0001	4.0231	0.00	*	-1.05	*	-0.75		-0.50		-0.49		-0.29		0.29		-0.54		-0.56		-0.25		-0.26		-0.01
28	Apolipoprotein A-II	APOA2	7.3142	0.0001	4.2545	0.00	*	-1.29	*	-0.84	*	-0.96		-0.65		-0.45		0.45		-0.33		-0.64		0.12		-0.19		-0.32
29	Apolipoprotein A-IV	APOA4	4.3298	0.0035	2.4605	0.01	*	-1.01		-0.09		-0.47		-0.32	*	-0.92	*	0.92		-0.54		-0.69		0.38		0.24		-0.14
30	Apolipoprotein C-I	APOC1	12.3770	0.0000	6.9402	0.00	*	-0.72	*	-1.37	*	-0.81		-0.52	*	0.64	*	-0.64		0.09		-0.21	*	-0.55	*	-0.85		-0.30
31	Apolipoprotein C-II	APOC2	3.3523	0.0144	1.8428	0.03		0.34		-0.46		-0.08		0.06	*	0.80	*	-0.80		0.41		0.27		-0.39		-0.53		-0.14
32	Apolipoprotein C-IV	APOC4	5.1112	0.0011	2.9451	0.00	*	1.86	*	1.28	*	1.41	*	1.37		0.58		-0.58		0.45		0.49		-0.13		-0.09		0.04
33	Apolipoprotein D	APOD	3.5999	0.0100	2.0001	0.02	*	-0.67		-0.17		-0.22		-0.33		-0.51		0.51		-0.46		-0.35		0.05		0.16		0.11
34	Apolipoprotein F	APOF	12.9640	0.0000	7.2248	0.00	*	-1.25	*	-1.19	*	-0.86	*	-0.97		-0.06		0.06		-0.39		-0.28		-0.33		-0.23		0.11
35	Apolipoprotein L3	APOL3	5.4106	0.0007	3.1281	0.00	*	-1.37		-0.36		-0.69		-0.46	*	-1.01	*	1.01		-0.68		-0.91		0.32		0.10		-0.23
36	Apolipoprotein M	APOM	4.9726	0.0014	2.8598	0.00		-0.32	*	-0.63	*	-0.59		-0.17		0.31		-0.31		0.27		-0.15		-0.03		-0.46		-0.42
37	Apolipoprotein(a)	LPA	3.4763	0.0120	1.9216	0.03	*	-0.63		-0.22		-0.27		-0.34		-0.41		0.41		-0.36		-0.29		0.06		0.12		0.07
38	Arf-GAP with Rho-GAP domain_ ANK repeat and PH domain-containing protein 2	ARAP2	7.7444	0.0000	4.4999	0.00		0.27	*	1.17		0.79		-0.06	*	-0.91	*	0.91		-0.52		0.33		0.38	*	1.24	*	0.85
39	Attractin	ATRN	8.3118	0.0000	4.8184	0.00	*	-1.27	*	-1.09	*	-0.92	*	-0.68		-0.18		0.18		-0.35		-0.59		-0.17		-0.41		-0.24
40	Baculoviral IAP repeat-containing protein 6	BIRC6	3.8878	0.0066	2.1825	0.02		0.28	*	0.94		0.57		0.28		-0.67		0.67		-0.30		0.00		0.37	*	0.66		0.30
41	Beta-2-microglobulin	B2M	12.8100	0.0000	7.1506	0.00	*	1.40	*	2.35	*	1.75		0.87		-0.96		0.96		-0.35		0.53		0.60	*	1.48		0.88
42	Beta-actin-like protein 2	ACTBL2	3.3906	0.0136	1.8671	0.03		0.43		0.40	*	0.64	*	0.57		0.03		-0.03		-0.21		-0.14		-0.24		-0.16		0.08
43	Beta-Ala-His dipeptidase	CNDP1	25.5840	0.0000	12.3560	0.00	*	-1.24	*	-1.78	*	-1.56	*	-0.93		0.54		-0.54		0.32		-0.31		-0.22	*	-0.85	*	-0.63
44	Biotinidase	BTD	5.2097	0.0010	3.0054	0.00		-0.32	*	-0.50	*	-0.53		-0.22		0.18		-0.18		0.21		-0.10		0.03		-0.28		-0.31
45	BTB/POZ domain-containing protein 10	BTBD10	7.2677	0.0001	4.2277	0.00		0.50	*	1.25	*	0.92		0.52	*	-0.74	*	0.74		-0.42		-0.01		0.33	*	0.73		0.40
46	C4b-binding protein alpha chain	C4BPA	3.2060	0.0178	1.7495	0.04	*	0.72		0.18		0.38		0.46		0.53		-0.53		0.33		0.25		-0.20		-0.28		-0.08
47	C4b-binding protein beta chain	C4BPB	4.5941	0.0024	2.6254	0.01		0.63	*	1.63		1.41		0.02		-1.01		1.01		-0.78		0.60		0.23	*	1.61		1.38
48	Calcium/calmodulin-dependent protein kinase kinase 1	CAMKK1	3.4160	0.0131	1.8833	0.03		0.79		0.10		0.12		0.54		0.69		-0.69		0.68		0.25		-0.02		-0.44		-0.43
49	Calcium-binding mitochondrial carrier protein SCaMC-2	SLC25A25	7.0487	0.0001	4.1013	0.00		0.43	*	1.20	*	1.59	*	1.21		-0.77		0.77	*	-1.15		-0.78		-0.39		-0.01		0.38
50	Calcium-binding protein 39	CAB39	5.6508	0.0005	3.2738	0.00	*	1.10	*	1.05	*	1.41		0.48		0.05		-0.05		-0.32		0.61		-0.37		0.57		0.93
51	Calcium-binding protein 5	CABP5	16.4760	0.0000	8.8302	0.00	*	2.00	*	2.62	*	2.36	*	1.67		-0.63		0.63		-0.36		0.32		0.27		0.95		0.68
52	Calmodulin-regulated spectrin-associated protein 2	CAMSAP2	20.0510	0.0000	10.3110	0.00	*	3.56	*	3.97	*	4.01	*	3.47		-0.41		0.41		-0.46		0.09		-0.05		0.50		0.54
53	Calreticulin	CALR	7.0860	0.0001	4.1229	0.00	*	0.03	*	-1.32	*	-1.12	*	-1.07		1.34		-1.34		1.15		1.10		-0.20		-0.24		-0.05
54	cAMP-dependent protein kinase catalytic subunit beta	PRKACB	3.6950	0.0087	2.0605	0.02		-0.10		-1.02	*	-1.43		-0.23		0.91		-0.91	*	1.33		0.13		0.41		-0.78	*	-1.20
55	Carboxypeptidase B2	CPB2	3.7233	0.0083	2.0784	0.02		0.02	*	-0.47		-0.28		-0.03	*	0.50	*	-0.50		0.30		0.06		-0.19	*	-0.44		-0.25
56	Carnitine O-palmitoyltransferase 1_ liver isoform	CPT1A	3.5294	0.0111	1.9554	0.03	*	1.03		1.00		0.44	*	1.05		0.03		-0.03		0.59		-0.03		0.56		-0.05		-0.61
57	Carnitine O-palmitoyltransferase 1_ muscle isoform	CPT1B	5.7855	0.0004	3.3551	0.00	*	-4.35	*	-4.28	*	-3.32		-2.05		-0.07		0.07		-1.03		-2.30		-0.96		-2.23		-1.27
58	Cartilage oligomeric matrix protein	COMP	3.0583	0.0221	1.6554	0.05		0.31	*	0.93		0.60		0.26		-0.63		0.63		-0.29		0.05		0.34		0.68		0.34
59	Cathepsin G	CTSG	9.9569	0.0000	5.7100	0.00	*	2.28	*	2.29	*	2.73	*	2.60		0.00		0.00		-0.45		-0.32		-0.44		-0.31		0.13
60	Caveolin-3	CAV3	12.1270	0.0000	6.8174	0.00	*	1.08	*	2.29	*	2.17	*	0.90	*	-1.20	*	1.20	*	-1.09		0.18		0.12	*	1.39	*	1.27
61	CD5 antigen-like	CD5L	8.0581	0.0000	4.6767	0.00	*	1.73	*	2.57	*	1.90		1.47		-0.83		0.83		-0.16		0.26		0.67	*	1.09		0.42
62	Centromere protein F	CENPF	3.2682	0.0162	1.7892	0.04	*	-0.55		-0.39		-0.38		-0.34		-0.16		0.16		-0.18		-0.22		-0.01		-0.05		-0.04
63	Centromere protein J	CENPJ	7.5922	0.0000	4.4135	0.00	*	-0.74	*	-0.72	*	-0.86		-0.48		-0.01		0.01		0.12		-0.26		0.14		-0.24		-0.38
64	Centrosomal protein of 89 kDa	CEP89	6.0023	0.0003	3.4852	0.00	*	-1.14		-0.70		-0.59		-0.47		-0.45		0.45		-0.55		-0.67		-0.10		-0.22		-0.12
65	Centrosome-associated protein 350	CEP350	9.3240	0.0000	5.3725	0.00	*	-0.92	*	-1.04	*	-1.00	*	-0.89		0.11		-0.11		0.08		-0.03		-0.04		-0.14		-0.11
66	Chromatin assembly factor 1 subunit A	CHAF1A	3.6429	0.0094	2.0274	0.02	*	1.57		0.74		0.54		0.67		0.83		-0.83	*	1.03		0.91		0.20		0.07		-0.13
67	Chymotrypsin-like elastase family member 3B	CELA3B	3.8612	0.0068	2.1657	0.02	*	-1.13		0.10		-0.48		-0.11	*	-1.24	*	1.24		-0.65		-1.02		0.58		0.21		-0.37
68	Cilia- and flagella-associated protein 36	CFAP36	5.8758	0.0004	3.4093	0.00	*	-1.58	*	-1.09		-0.90		-0.52		-0.49		0.49		-0.68	*	-1.06		-0.20		-0.57		-0.38
69	Cingulin	CGN	3.3719	0.0140	1.8552	0.03		-0.39		0.06		-0.28		-0.64		-0.45		0.45		-0.10		0.26		0.34	*	0.70		0.36
70	Clusterin	CLU	3.6567	0.0092	2.0362	0.02	*	-0.48		-0.36		-0.40	*	-0.42		-0.12		0.12		-0.08		-0.06		0.04		0.07		0.02
71	CMT1A duplicated region transcript 15 protein	CDRT15	7.3398	0.0001	4.2691	0.00	*	-1.99	*	-1.15	*	-2.01		-1.22		-0.84		0.84		0.03		-0.77		0.86		0.07		-0.80
72	Coagulation factor IX	F9	3.3356	0.0147	1.8321	0.03	*	0.41		0.07		0.29		0.16	*	0.34	*	-0.34		0.12		0.25		-0.23		-0.09		0.13
73	Coagulation factor X	F10	3.0490	0.0224	1.6494	0.05		-0.24	*	-0.35		-0.26		-0.31		0.11		-0.11		0.03		0.07		-0.08		-0.04		0.05
74	Cohesin subunit SA-3	STAG3	5.1505	0.0011	2.9692	0.00		-0.29		0.18		-0.04	*	-0.95		-0.47		0.47		-0.24		0.67		0.22	*	1.13	*	0.91
75	Coiled-coil domain-containing protein 121	CCDC121	4.6065	0.0023	2.6331	0.01		0.47		-0.28		0.11		0.57	*	0.76	*	-0.76		0.36		-0.10		-0.39	*	-0.85		-0.46
76	Coiled-coil domain-containing protein 150	CCDC150	3.3535	0.0143	1.8435	0.03		-0.54	*	-0.89		-0.58		-0.12		0.35		-0.35		0.04		-0.42		-0.31	*	-0.77		-0.45
77	Coiled-coil domain-containing protein 201	CCDC201	7.7458	0.0000	4.5007	0.00	*	1.92	*	1.25	*	1.38	*	1.66		0.68		-0.68		0.54		0.27		-0.13		-0.41		-0.27
78	Complement C1q subcomponent subunit A	C1QA	14.7390	0.0000	8.0564	0.00	*	-1.20	*	-0.63	*	-0.84	*	-0.93	*	-0.57	*	0.57		-0.36		-0.27		0.21		0.30		0.10
79	Complement C4-A	C4A	3.1287	0.0199	1.7003	0.04		0.09	*	-0.87		-0.10		-0.26	*	0.96	*	-0.96		0.19		0.35		-0.77		-0.60		0.16
80	Complement component 1 Q subcomponent-binding protein_ mitochondrial	C1QBP	10.6580	0.0000	6.0760	0.00		0.62	*	1.34	*	1.00		0.50	*	-0.73	*	0.73		-0.38		0.12		0.35	*	0.85		0.50
81	Complement component C8 alpha chain	C8A	6.5872	0.0001	3.8320	0.00		-0.28	*	-0.62	*	-0.49	*	-0.44		0.34		-0.34		0.21		0.16		-0.13		-0.18		-0.05
82	Complement component C8 gamma chain	C8G	3.8719	0.0067	2.1724	0.02		-0.38	*	-0.57	*	-0.47		-0.41		0.18		-0.18		0.09		0.03		-0.10		-0.16		-0.06
83	Complement factor H-related protein 1	CFHR1	5.8011	0.0004	3.3645	0.00		-0.16	*	-0.79		-0.24		-0.37	*	0.63	*	-0.63		0.07		0.21		-0.55		-0.42		0.13
84	Complement factor H-related protein 3	CFHR3	4.2009	0.0042	2.3797	0.01		0.81		-0.69	*	-0.94		-0.68		1.50		-1.50		1.75		1.49		0.25		0.00		-0.25
85	Complexin-4	CPLX4	8.3028	0.0000	4.8134	0.00	*	-0.95		-0.21		0.12		-0.18	*	-0.73	*	0.73	*	-1.07	*	-0.76		-0.33		-0.03		0.30
86	Coronin-2A	CORO2A	4.0410	0.0053	2.2791	0.01		-1.30	*	-1.87		-2.24		0.20		0.57		-0.57		0.93		-1.51		0.37	*	-2.07		-2.44
87	C-reactive protein	CRP	3.8296	0.0071	2.1457	0.02	*	1.41		0.46		1.30		0.38		0.94		-0.94		0.10		1.03		-0.84		0.09		0.93
88	Differentially expressed in FDCP 8 homolog	DEF8	19.8170	0.0000	10.2180	0.00	*	-1.58	*	-1.12	*	-1.07	*	-1.23		-0.47		0.47		-0.51		-0.36		-0.05		0.11		0.16
89	DNA ligase 4	LIG4	5.5962	0.0006	3.2408	0.00	*	-0.86	*	-0.70	*	-0.69		-0.49		-0.16		0.16		-0.17		-0.37		0.00		-0.21		-0.20
90	DNA repair protein XRCC2	XRCC2	6.8820	0.0001	4.0045	0.00	*	-3.38	*	-2.66	*	-2.45		-1.31		-0.72		0.72		-0.94	*	-2.07		-0.21		-1.35		-1.14
91	DnaJ homolog subfamily B member 3	DNAJB3	5.9403	0.0004	3.4480	0.00		1.31	*	-3.21	*	-2.53	*	-2.07		4.52		-4.52		3.85		3.38		-0.67		-1.14		-0.46
92	DPY30 domain-containing protein 2	DYDC2	5.2151	0.0010	3.0087	0.00	*	-1.58		-0.57	*	-1.26	*	-1.82		-1.01		1.01		-0.32		0.24		0.69		1.25		0.57
93	Dual specificity protein kinase TTK	TTK	84.1620	0.0000	25.2670	0.00	*	4.53	*	3.82	*	4.58	*	4.53		0.71		-0.71		-0.05		0.00		-0.76		-0.71		0.05
94	Dynein axonemal heavy chain 17	DNAH17	7.5777	0.0000	4.4052	0.00	*	-0.41		0.26		-0.03		-0.31	*	-0.67	*	0.67		-0.37		-0.10		0.30	*	0.57		0.27
95	Dynein axonemal heavy chain 2	DNAH2	3.2782	0.0160	1.7956	0.04		0.10	*	-0.52		-0.23		-0.13	*	0.62	*	-0.62		0.33		0.23		-0.29		-0.39		-0.10
96	Dynein axonemal heavy chain 7	DNAH7	7.8056	0.0000	4.5345	0.00	*	-2.98	*	-2.93	*	-2.47		-1.31		-0.05		0.05		-0.51		-1.67		-0.46		-1.62		-1.16
97	EF-hand calcium-binding domain-containing protein 3	EFCAB3	6.5614	0.0002	3.8169	0.00	*	-1.01	*	-0.82	*	-0.69		-0.38		-0.19		0.19		-0.32	*	-0.62		-0.13		-0.43		-0.31
98	EGF-containing fibulin-like extracellular matrix protein 1	EFEMP1	3.7155	0.0084	2.0735	0.02	*	-0.62		-0.12		-0.45		-0.40		-0.51		0.51		-0.18		-0.22		0.33		0.29		-0.04
99	Electron transfer flavoprotein beta subunit lysine methyltransferase	ETFBKMT	3.2518	0.0166	1.7787	0.04	*	4.53		1.37		0.38		3.14		3.16		-3.16		4.16		1.40		1.00		-1.76		-2.76
100	ELKS/Rab6-interacting/CAST family member 1	ERC1	7.1220	0.0001	4.1437	0.00	*	-1.22	*	-0.74		-0.47		-0.52		-0.48		0.48	*	-0.75	*	-0.70		-0.27		-0.22		0.05
101	Endoplasmic reticulum chaperone BiP	HSPA5	4.8201	0.0017	2.7656	0.01	*	-0.32		-0.55		-0.43	*	-1.45		0.23		-0.23		0.11		1.13		-0.12		0.90		1.01
102	EP300-interacting inhibitor of differentiation 1	EID1	12.1420	0.0000	6.8246	0.00	*	-1.24	*	-1.50	*	-1.21	*	-1.17		0.26		-0.26		-0.03		-0.08		-0.29		-0.33		-0.04
103	Ephrin type-A receptor 5	EPHA5	3.1710	0.0187	1.7272	0.04		-0.41		-0.37	*	-0.63		-0.40		-0.04		0.04		0.22		-0.01		0.26		0.03		-0.23
104	Eukaryotic peptide chain release factor GTP-binding subunit ERF3B	GSPT2	3.0164	0.0235	1.6286	0.05		0.78		0.39	*	1.20		-0.10		0.39		-0.39		-0.42		0.87		-0.81		0.49		1.30
105	Eukaryotic translation initiation factor 2 subunit 2	EIF2S2	3.1241	0.0201	1.6974	0.04	*	0.63		0.54		0.35		0.51		0.09		-0.09		0.29		0.13		0.20		0.03		-0.16
106	Exocyst complex component 4	EXOC4	6.3505	0.0002	3.6924	0.00		0.36	*	1.05		0.65		0.18	*	-0.69	*	0.69		-0.29		0.18		0.39	*	0.87		0.48
107	Exosome complex exonuclease RRP44	DIS3	4.3370	0.0034	2.4650	0.01	*	-3.34	*	-2.93		-2.24		-1.17		-0.41		0.41		-1.10		-2.16		-0.69		-1.76		-1.07
108	FAS-associated factor 2	FAF2	3.3390	0.0146	1.8343	0.03	*	0.45	*	0.50		0.30		0.20		-0.05		0.05		0.15		0.25		0.20		0.30		0.10
109	Fatty-acid amide hydrolase 2	FAAH2	3.1771	0.0186	1.7311	0.04		-0.08	*	-0.56		-0.46		-0.29		0.49		-0.49		0.39		0.22		-0.10		-0.27		-0.17
110	Ferritin heavy chain	FTH1	4.2222	0.0040	2.3931	0.01		-0.41	*	-0.68	*	-0.88		-0.45		0.27		-0.27		0.46		0.04		0.20		-0.22		-0.42
111	Ferritin light chain	FTL	4.1433	0.0045	2.3435	0.01	*	4.44		1.05		1.08		1.25		3.39		-3.39		3.36		3.19		-0.04		-0.20		-0.17
112	Fibrous sheath-interacting protein 1	FSIP1	3.5339	0.0110	1.9583	0.03	*	-0.68		-0.21	*	-0.64		-0.12		-0.47		0.47		-0.04		-0.56		0.43		-0.09		-0.52
113	Fumarate hydratase_ mitochondrial	FH	3.6153	0.0098	2.0099	0.02	*	-0.67		-0.48	*	-0.68		-0.44		-0.18		0.18		0.01		-0.23		0.20		-0.05		-0.24
114	G patch domain-containing protein 8	GPATCH8	9.0321	0.0000	5.2146	0.00	*	2.15	*	2.26	*	2.39	*	2.20		-0.11		0.11		-0.24		-0.05		-0.13		0.06		0.19
115	Galectin-3-binding protein	LGALS3BP	12.1060	0.0000	6.8067	0.00	*	1.09	*	2.24	*	1.40		0.74		-1.15		1.15		-0.31		0.35		0.84	*	1.51		0.66
116	Gasdermin-C	GSDMC	3.6583	0.0092	2.0372	0.02	*	1.19		0.64		0.60		0.61		0.55		-0.55		0.59		0.58		0.05		0.03		-0.01
117	Gelsolin	GSN	6.4733	0.0002	3.7650	0.00	*	-0.90		-0.13	*	-0.73		-0.51	*	-0.77	*	0.77		-0.18		-0.39		0.59		0.37		-0.22
118	Girdin	CCDC88A	20.1940	0.0000	10.3680	0.00	*	-0.97	*	-0.76	*	-0.78	*	-0.58		-0.21		0.21		-0.19	*	-0.39		0.02		-0.18		-0.20
119	Glial fibrillary acidic protein	GFAP	5.8734	0.0004	3.4079	0.00	*	1.39	*	1.03	*	1.45	*	1.05		0.36		-0.36		-0.05		0.34		-0.41		-0.02		0.40
120	Glucose-6-phosphate 1-dehydrogenase	G6PD	2.9886	0.0245	1.6109	0.05		-0.73		-0.49		-0.30		0.16		-0.24		0.24		-0.43	*	-0.89		-0.19		-0.65		-0.45
121	Glucosylceramide transporter ABCA12	ABCA12	12.6400	0.0000	7.0682	0.00	*	1.13	*	1.75	*	1.45	*	0.95		-0.62		0.62		-0.32		0.19		0.30	*	0.80		0.51
122	Glutathione S-transferase Mu 2	GSTM2	3.5022	0.0115	1.9381	0.03		0.15	*	0.40	*	0.55		0.30		-0.26		0.26		-0.41		-0.16		-0.15		0.10		0.25
123	Glycine N-acyltransferase-like protein 1	GLYATL1	4.1152	0.0047	2.3258	0.01		-0.26		0.29		-0.40		0.43		-0.55		0.55		0.13		-0.69		0.68		-0.14	*	-0.82
124	Golgin subfamily A member 8C	GOLGA8CP	3.3557	0.0143	1.8449	0.03	*	0.61	*	0.56		0.35		0.42		0.05		-0.05		0.26		0.19		0.21		0.14		-0.07
125	Grainyhead-like protein 2 homolog	GRHL2	4.0229	0.0054	2.2677	0.01	*	-0.69		-0.02	*	-0.76		-0.69		-0.67		0.67		0.07		-0.01		0.74		0.66		-0.08
126	Haptoglobin	HP	5.2150	0.0010	3.0087	0.00	*	3.69		1.08		2.02		1.25	*	2.61	*	-2.61	*	1.66		2.44		-0.95		-0.17		0.77
127	Heat shock protein HSP 90-alpha A2	HSP90AA2P	4.2831	0.0037	2.4312	0.01		0.70		-0.51		-0.51		0.50		1.21		-1.21	*	1.21		0.19		0.00		-1.01	*	-1.01
128	Hemopexin	HPX	4.7671	0.0019	2.7328	0.01	*	-0.76	*	-0.73		-0.62		-0.54		-0.03		0.03		-0.14		-0.22		-0.11		-0.19		-0.08
129	Hepatocyte growth factor activator	HGFAC	3.1493	0.0193	1.7134	0.04		-1.10	*	-1.40	*	-1.44		-0.96		0.30		-0.30		0.33		-0.14		0.03		-0.44		-0.48
130	Histidine protein methyltransferase 1 homolog	METTL18	3.0115	0.0237	1.6255	0.05		0.48		-0.18		0.38		0.09	*	0.66	*	-0.66		0.10		0.39		-0.56		-0.27		0.29
131	Histidine-rich glycoprotein	HRG	4.0473	0.0052	2.2831	0.01	*	-0.65	*	-0.68	*	-0.64		-0.45		0.03		-0.03		-0.01		-0.21		-0.04		-0.23		-0.19
132	Histone-lysine N-methyltransferase 2A	KMT2A	5.2966	0.0009	3.0586	0.00	*	-0.71		-0.19		-0.21		-0.36		-0.52		0.52	*	-0.50		-0.35		0.02		0.17		0.15
133	Homeobox protein Hox-C10	HOXC10	7.7983	0.0000	4.5304	0.00	*	1.22		0.37	*	0.79	*	0.94	*	0.85	*	-0.85		0.43		0.28		-0.42		-0.57		-0.15
134	Homeobox protein Hox-C5	HOXC5	8.6312	0.0000	4.9952	0.00	*	-0.70	*	-1.25	*	-0.90		-0.33		0.55		-0.55		0.20		-0.36		-0.35	*	-0.91		-0.57
135	Homeobox protein TGIF1	TGIF1	13.0580	0.0000	7.2701	0.00	*	1.04	*	1.63	*	1.44	*	0.94		-0.59		0.59		-0.39		0.10		0.20	*	0.69		0.50
136	Huntingtin-interacting protein 1	HIP1	9.9033	0.0000	5.6817	0.00	*	-0.92		-0.47		-0.34		-0.34	*	-0.46	*	0.46	*	-0.59	*	-0.58		-0.13		-0.13		0.00
137	Immunoglobulin heavy constant alpha 1	IGHA1	6.7060	0.0001	3.9017	0.00	*	5.74	*	6.79	*	7.22		4.44		-1.05		1.05		-1.48		1.30		-0.42		2.35		2.78
138	Immunoglobulin heavy constant alpha 2	IGHA2	3.4456	0.0125	1.9021	0.03		0.22		1.15		1.49		-0.04		-0.92		0.92		-1.26		0.27		-0.34		1.19	*	1.53
139	Immunoglobulin heavy constant gamma 2	IGHG2	14.7950	0.0000	8.0820	0.00	*	0.90	*	1.87	*	1.63	*	0.85	*	-0.97	*	0.97		-0.73		0.05		0.24	*	1.01		0.77
140	Immunoglobulin heavy constant gamma 3	IGHG3	9.8273	0.0000	5.6415	0.00	*	2.16	*	2.96	*	2.04	*	2.37		-0.81		0.81		0.12		-0.21		0.92		0.60		-0.33
141	Immunoglobulin heavy constant mu	IGHM	4.4385	0.0030	2.5285	0.01		1.80	*	2.66	*	1.79		1.67		-0.85		0.85		0.02		0.13		0.87		0.98		0.11
142	Immunoglobulin J chain	JCHAIN	5.8791	0.0004	3.4114	0.00	*	1.42	*	2.19	*	1.86	*	1.36		-0.77		0.77		-0.43		0.07		0.33		0.83		0.50
143	Immunoglobulin kappa variable 1-12	IGKV1-12	6.8484	0.0001	3.9850	0.00	*	3.30	*	4.17	*	4.80	*	2.53		-0.87		0.87		-1.49		0.77		-0.62		1.64		2.27
144	Immunoglobulin kappa variable 1D-13	IGKV1D-13	3.6621	0.0091	2.0396	0.02		2.76	*	2.56		1.03		0.11		0.21		-0.21		1.73		2.66		1.52	*	2.45		0.93
145	Immunoglobulin kappa variable 2D-29	IGKV2D-29	10.8630	0.0000	6.1817	0.00	*	3.54	*	4.34	*	4.41	*	3.43		-0.81		0.81		-0.88		0.11		-0.07		0.91		0.98
146	Immunoglobulin lambda constant 2	IGLC2	10.8710	0.0000	6.1857	0.00	*	3.95	*	4.89	*	4.61	*	3.25		-0.94		0.94		-0.66		0.70		0.28		1.65		1.36
147	Immunoglobulin lambda constant 7	IGLC7	4.1533	0.0045	2.3498	0.01	*	0.92		0.68		0.69		0.17		0.24		-0.24		0.23	*	0.75		-0.01		0.51		0.52
148	Immunoglobulin lambda variable 5-45	IGLV5-45	5.1624	0.0011	2.9764	0.00	*	2.49		0.37		0.58	*	0.79		2.12		-2.12		1.91		1.69		-0.21		-0.43		-0.21
149	Indoleamine 2_3-dioxygenase 1	IDO1	9.0441	0.0000	5.2211	0.00	*	2.30	*	1.96	*	2.28	*	2.31		0.34		-0.34		0.02		-0.01		-0.32		-0.35		-0.04
150	Inhibitor of Bruton tyrosine kinase	IBTK	13.8400	0.0000	7.6410	0.00	*	-2.00	*	-1.94	*	-1.77	*	-1.20		-0.06		0.06		-0.23		-0.80		-0.17		-0.74		-0.57
151	Insulin-like growth factor-binding protein 3	IGFBP3	10.8400	0.0000	6.1698	0.00	*	-1.56		-0.87	*	-1.66		-0.61	*	-0.69	*	0.69		0.10	*	-0.95		0.79		-0.26	*	-1.05
152	Insulin-like growth factor-binding protein complex acid labile subunit	IGFALS	11.0820	0.0000	6.2936	0.00	*	-0.80	*	-1.19	*	-1.17		-0.57		0.39		-0.39		0.37		-0.23		-0.03	*	-0.63		-0.60
153	Integrator complex subunit 6-like	INTS6L	11.5050	0.0000	6.5076	0.00	*	-1.49	*	-1.21	*	-1.11	*	-0.97		-0.28		0.28		-0.38		-0.51		-0.09		-0.23		-0.14
154	Integrin beta-1-binding protein 1	ITGB1BP1	8.1457	0.0000	4.7258	0.00	*	-0.92	*	-0.93	*	-0.89	*	-0.60		0.00		0.00		-0.03		-0.33		-0.04		-0.33		-0.29

Supplementary Table S3. Overview on the statistical analysis
* = p < 0.05

				Tukey's HSD			iCCA-HC		HCC-HC		CIR-HC		PSC-HC		iCCA-HCC		HCC-iCCA		iCCA-CIR		iCCA-PSC		HCC-CIR		HCC-PSC		CIR-PSC	
No.	Protein name	Gene	f.value	p.value	minus L	FDR	p < 0.05	log2FC	p < 0.05	log2FC	p < 0.05	log2FC	p < 0.05	log2FC	p < 0.05	log2FC	p < 0.05	log2FC	p < 0.05	log2FC	p < 0.05	log2FC	p < 0.05	log2FC	p < 0.05	log2FC	p < 0.05	log2FC
155	Inter-alpha-trypsin inhibitor heavy chain H1	ITIH1	3.3322	0.0148	1.8299	0.03		-0.31	*	-0.57		-0.29		-0.44		0.26		-0.26		-0.02		0.13		-0.28		-0.13		0.15
156	Inter-alpha-trypsin inhibitor heavy chain H2	ITIH2	4.4710	0.0028	2.5487	0.01		-0.37	*	-0.61	*	-0.52	*	-0.55		0.24		-0.24		0.14		0.17		-0.09		-0.06		0.03
157	Intercellular adhesion molecule 2	ICAM2	6.7050	0.0001	3.9012	0.00		5.60	*	6.49	*	6.81		4.15		-0.89		0.89		-1.21		1.45		-0.32		2.34		2.66
158	Interleukin-1 receptor accessory protein-like 1	IL1RAPL1	4.7197	0.0020	2.7034	0.01		-0.10	*	-0.69		-0.42		-0.30	*	0.58	*	-0.58		0.32		0.19		-0.27		-0.39		-0.12
159	IQCJ-SCHIP1 readthrough transcript protein	IQCJ-SCHIP1	10.1300	0.0000	5.8012	0.00	*	-0.95	*	-0.77	*	-0.63	*	-0.56		-0.19		0.19		-0.32		-0.39		-0.14		-0.21		-0.07
160	Kallistatin	SERPINA4	13.3190	0.0000	7.3946	0.00	*	-0.95	*	-0.60	*	-0.75	*	-0.65		-0.35		0.35		-0.20		-0.31		0.15		0.04		-0.11
161	KAT8 regulatory NSL complex subunit 3	KANSL3	9.8797	0.0000	5.6692	0.00		-0.56	*	-1.37	*	-1.01	*	-0.82	*	0.82	*	-0.82		0.45		0.27		-0.37	*	-0.55		-0.19
162	Kelch-like protein 25	KLHL25	10.3800	0.0000	5.9316	0.00	*	1.27	*	1.31	*	1.40	*	1.02		-0.03		0.03		-0.13		0.25		-0.10		0.29		0.38
163	Keratin_ type I cytoskeletal 20	KRT20	6.8431	0.0001	3.9818	0.00	*	1.06	*	1.35	*	0.74	*	1.23		-0.28		0.28		0.33		-0.17		0.61		0.12		-0.49
164	Keratin_ type II cytoskeletal 4	KRT4	7.7220	0.0000	4.4872	0.00	*	1.22	*	1.35	*	1.44	*	0.91		-0.13		0.13		-0.22		0.31		-0.08		0.44		0.53
165	Keratin_ type II cytoskeletal 5	KRT5	3.0479	0.0225	1.6487	0.05		-1.53	*	-1.77		-1.58		-1.14		0.24		-0.24		0.04		-0.40		-0.20		-0.64		-0.44
166	Keratin_ type II cytoskeletal 80	KRT80	5.0802	0.0012	2.9260	0.00	*	-2.71	*	-2.62	*	-2.03		-1.22		-0.09		0.09		-0.68		-1.49		-0.59		-1.40		-0.82
167	Kinesin heavy chain isoform 5C	KIF5C	6.9643	0.0001	4.0524	0.00	*	-0.72	*	-0.54	*	-0.47		-0.37		-0.18		0.18		-0.25		-0.35		-0.07		-0.17		-0.10
168	Kinesin-like protein KIF20B	KIF20B	4.3942	0.0032	2.5007	0.01	*	-0.64	*	-0.68		-0.36		-0.29		0.03		-0.03		-0.28		-0.35		-0.31		-0.38		-0.07
169	Kinesin-like protein KIF21B	KIF21B	9.8338	0.0000	5.6449	0.00	*	-1.10	*	-1.29	*	-1.63	*	-1.44		0.19		-0.19		0.53		0.34		0.34		0.16		-0.18
170	Leucine-rich alpha-2-glycoprotein	LRG1	3.9039	0.0064	2.1926	0.02	*	1.16		0.17		0.86		0.21	*	0.99	*	-0.99		0.30	*	0.95		-0.69		-0.04		0.65
171	Liprin-beta-1	PPFIBP1	3.5841	0.0102	1.9901	0.02		-0.26		0.48		0.05		-0.33	*	-0.75	*	0.75		-0.31		0.06		0.44	*	0.81		0.37
172	Long-chain-fatty-acid--CoA ligase 5	ACSL5	3.0296	0.0231	1.6371	0.05		-0.48		-0.10		-0.24		0.03		-0.38		0.38		-0.24	*	-0.51		0.14		-0.13		-0.28
173	Lumican	LUM	4.6859	0.0021	2.6825	0.01	*	-1.66		-0.95		-1.12		-0.78		-0.71		0.71		-0.54		-0.88		0.17		-0.17		-0.34
174	MAP7 domain-containing protein 1	MAP7D1	4.0303	0.0053	2.2724	0.01		-0.49		-0.56		-0.43		-1.04		0.07		-0.07		-0.06		0.55		-0.14		0.47		0.61
175	Melanoma-associated antigen 2	MAGEA2	4.9572	0.0014	2.8503	0.00	*	-1.22		-0.81		-0.56		0.13		-0.40		0.40		-0.66	*	-1.34		-0.26		-0.94		-0.68
176	Melanophilin	MLPH	3.5803	0.0103	1.9877	0.02		0.35		-0.44		0.01		-0.57		0.80		-0.80		0.34	*	0.93		-0.45		0.13		0.58
177	Methylmalonate-semialdehyde dehydrogenase [acylating]_ mitochondrial	ALDH6A1	4.0687	0.0051	2.2965	0.01		0.27	*	0.76		0.56		0.26		-0.49		0.49		-0.29		0.01		0.20		0.50		0.30
178	Microtubule-associated protein 1A	MAP1A	7.8208	0.0000	4.5431	0.00		0.09	*	1.17	*	1.03		0.16	*	-1.08	*	1.08	*	-0.94		-0.07		0.14	*	1.01	*	0.87
179	Mitogen-activated protein kinase kinase kinase 15	MAP3K15	4.2550	0.0039	2.4136	0.01		-0.24		-0.51	*	-0.61	*	-0.67		0.27		-0.27		0.37		0.43		0.10		0.16		0.06
180	Mitoguardin 2	MIGA2	4.2138	0.0041	2.3877	0.01		0.91	*	1.30		0.80	*	1.32		-0.39		0.39		0.11		-0.41		0.50		-0.02		-0.51
181	M-phase inducer phosphatase 1	CDC25A	7.4636	0.0000	4.3401	0.00	*	-0.99	*	-0.52		-0.45		-0.37		-0.48		0.48	*	-0.55	*	-0.62		-0.07		-0.15		-0.08
182	Myosin regulatory light chain 2_ skeletal muscle isoform	MYLPF	8.6157	0.0000	4.9867	0.00		0.59	*	1.36	*	1.10		0.47	*	-0.77	*	0.77		-0.51		0.12		0.26	*	0.89		0.63
183	Myosin-15	MYH15	7.1547	0.0001	4.1626	0.00	*	0.89	*	0.60	*	0.64	*	1.03		0.30		-0.30		0.25		-0.14		-0.04		-0.43		-0.39
184	NACHT_ LRR and PYD domains-containing protein 14	NLRP14	3.7922	0.0076	2.1221	0.02	*	0.69		0.06		0.22		0.04	*	0.63	*	-0.63		0.47	*	0.66		-0.16		0.03		0.19
185	Neurofibromin	NF1	5.3123	0.0009	3.0681	0.00		0.08	*	0.84	*	0.87		-0.06	*	-0.76	*	0.76	*	-0.79		0.13		-0.04	*	0.89	*	0.93
186	NFX1-type zinc finger-containing protein 1	ZNFX1	14.4300	0.0000	7.9149	0.00	*	0.99	*	1.94	*	1.38	*	1.14	*	-0.94	*	0.94		-0.39		-0.14		0.55	*	0.80		0.25
187	Nicotinamide N-methyltransferase	NNMT	7.2369	0.0001	4.2100	0.00	*	-1.15	*	-0.56	*	-0.69		-0.56		-0.59		0.59		-0.46	*	-0.59		0.13		0.00		-0.13
188	Noelin	OLFM1	7.2836	0.0001	4.2369	0.00	*	1.31	*	1.08	*	1.19	*	1.37		0.23		-0.23		0.12		-0.06		-0.11		-0.29		-0.18
189	Nucleoredoxin	NXN	7.5473	0.0000	4.3879	0.00	*	-1.43	*	-2.41	*	-1.71	*	-1.84		0.98		-0.98		0.28		0.40		-0.70		-0.58		0.12
190	Nucleoside diphosphate kinase 7	NME7	11.7760	0.0000	6.6431	0.00	*	0.76	*	1.51	*	1.13		0.72	*	-0.75	*	0.75		-0.38		0.03		0.37	*	0.78		0.41
191	NXPE family member 2	NXPE2	4.7770	0.0018	2.7389	0.01	*	-0.72		-0.35	*	-0.61		-0.26		-0.37		0.37		-0.11	*	-0.46		0.26		-0.09		-0.35
192	Obscurin	OBSCN	8.3791	0.0000	4.8558	0.00	*	-0.79	*	-1.14	*	-0.88		-0.61		0.35		-0.35		0.09		-0.18		-0.26	*	-0.53		-0.27
193	ORC ubiquitin ligase 1	OBI1	8.0819	0.0000	4.6901	0.00		-0.43	*	1.22	*	1.77		0.25	*	-1.64	*	1.64	*	-2.20		-0.68		-0.56		0.97	*	1.53
194	Paired box protein Pax-9	PAX9	8.4594	0.0000	4.9004	0.00		0.32	*	1.41	*	1.48		0.34	*	-1.09	*	1.09	*	-1.17		-0.02		-0.07	*	1.08	*	1.15
195	Pantetheinase	VNN1	4.2450	0.0039	2.4073	0.01	*	-0.59	*	-0.61	*	-0.73		-0.55		0.02		-0.02		0.14		-0.04		0.12		-0.06		-0.18
196	PAS domain-containing serine/threonine-protein kinase	PASK	6.2190	0.0002	3.6144	0.00	*	-1.38	*	-2.59	*	-2.12		-1.10		1.21		-1.21		0.74		-0.28		-0.47		-1.49		-1.02
197	Peptidyl-prolyl cis-trans isomerase FKBP4	FKBP4	7.1444	0.0001	4.1566	0.00		0.27	*	0.87	*	0.56		0.28	*	-0.60	*	0.60		-0.29		-0.01		0.32	*	0.59		0.28
198	Peroxisomal trans-2-enoyl-CoA reductase	PECR	4.6600	0.0022	2.6664	0.01	*	-1.37		-0.16		-0.19		-0.90	*	-1.21	*	1.21	*	-1.18		-0.47		0.04		0.74		0.70
199	Phosphatidylcholine-sterol acyltransferase	LCAT	5.0940	0.0012	2.9345	0.00	*	-0.98		-0.54		-0.58		-0.38		-0.45		0.45		-0.40	*	-0.61		0.04		-0.16		-0.20
200	Phosphatidylinositol 5-phosphate 4-kinase type-2 alpha	PIP4K2A	3.6321	0.0095	2.0206	0.02		3.21		4.17	*	4.32		0.36		-0.96		0.96		-1.10		2.86		-0.14		3.82		3.96
201	Phosphatidylinositol 5-phosphate 4-kinase type-2 gamma	PIP4K2C	3.0723	0.0217	1.6643	0.05	*	-2.11		-1.94		-1.74		-0.84		-0.17		0.17		-0.37		-1.28		-0.20		-1.11		-0.91
202	Phospholipid-transporting ATPase IH	ATP11A	5.0390	0.0013	2.9007	0.00	*	-1.03	*	-1.43	*	-1.16	*	-1.47		0.40		-0.40		0.13		0.44		-0.27		0.04		0.31
203	Plasma serine protease inhibitor	SERPINA5	4.5903	0.0024	2.6231	0.01	*	-1.24		-0.32		-0.62		-0.71		-0.91		0.91		-0.61		-0.53		0.30		0.38		0.08
204	Plasminogen	PLG	8.3130	0.0000	4.8191	0.00	*	-0.79	*	-0.66	*	-0.80	*	-0.68		-0.13		0.13		0.01		-0.11		0.14		0.02		-0.13
205	Plasminogen-like protein A	PLGLA	3.4189	0.0130	1.8851	0.03	*	-1.53		-1.00		-0.64		-1.07		-0.53		0.53		-0.90		-0.46		-0.37		0.07		0.43
206	Platelet basic protein	PPBP	6.6297	0.0001	3.8570	0.00		-0.58	*	-1.06	*	-1.00		-0.72		0.48		-0.48		0.41		0.14		-0.07		-0.34		-0.27
207	Platelet factor 4	PF4	11.1400	0.0000	6.3227	0.00	*	-0.96	*	-1.57	*	-1.46	*	-1.07		0.61		-0.61		0.49		0.11		-0.12		-0.50		-0.39
208	Pleckstrin homology domain-containing family S member 1	PLEKHS1	3.6140	0.0098	2.0091	0.02	*	4.44		0.18		0.55		1.11		4.26		-4.26		3.89		3.32		-0.37		-0.93		-0.57
209	Polypeptide N-acetylgalactosaminyltransferase 17	GALNT17	5.1797	0.0010	2.9870	0.00	*	-1.32	*	-1.38	*	-0.98		-0.62		0.07		-0.07		-0.34		-0.70		-0.40		-0.76		-0.36
210	Prefoldin subunit 1	PFDN1	5.6348	0.0005	3.2641	0.00		0.33		0.80	*	1.16		0.50		-0.47		0.47	*	-0.83		-0.17		-0.36		0.30		0.66
211	Pregnancy zone protein	PZP	6.6080	0.0001	3.8443	0.00	*	-0.72	*	-0.89	*	-0.88	*	-0.78		0.17		-0.17		0.16		0.06		0.00		-0.11		-0.11
212	PRELI domain-containing protein 2	PRELID2	4.3662	0.0033	2.4833	0.01	*	-0.65		-0.11		-0.19	*	-0.76		-0.54		0.54		-0.46		0.11		0.08		0.65		0.57
213	Probable asparagine--tRNA ligase_ mitochondrial	NARS2	5.0356	0.0013	2.8986	0.00		-0.67		-0.89		-1.04		-1.57		0.22		-0.22		0.37	*	0.91		0.15		0.68		0.53
214	Probable ATP-dependent RNA helicase DDX17	DDX17	4.3378	0.0034	2.4655	0.01	*	0.80		0.52		0.63		0.56		0.28		-0.28		0.17		0.24		-0.11		-0.04		0.07
215	Probable non-functional immunoglobulin kappa variable 2D-24	IGKV2D-24	11.6490	0.0000	6.5798	0.00	*	2.03	*	3.84	*	3.51	*	2.53	*	-1.82	*	1.82		-1.48		-0.51		0.34		1.31		0.97
216	Probable rRNA-processing protein EBP2	EBNA1BP2	3.9280	0.0062	2.2079	0.02	*	-0.70		-0.50		-0.17		0.06		-0.20		0.20		-0.53	*	-0.76		-0.33		-0.55		-0.23
217	Profilin-1	PFN1	3.5350	0.0110	1.9589	0.03	*	-0.80		-0.12	*	-0.72		-0.78		-0.68		0.68		-0.08		-0.01		0.60		0.67		0.07
218	Proline/serine-rich coiled-coil protein 1	PSRC1	5.3709	0.0008	3.1039	0.00	*	-0.90		-0.40		-0.12		-0.29		-0.49		0.49	*	-0.78		-0.60		-0.28		-0.11		0.17
219	Prostaglandin-H2 D-isomerase	PTGDS	7.8091	0.0000	4.5365	0.00	*	3.68	*	2.92	*	2.51	*	1.94		0.75		-0.75		1.16		1.73		0.41		0.98		0.57
220	Proteasomal ATPase-associated factor 1	PAAF1	3.1423	0.0195	1.7089	0.04		-0.27		0.22		-0.31		-0.59		-0.49		0.49		0.04		0.32		0.53	*	0.81		0.28
221	Protein CIP2A	CIP2A	4.7453	0.0019	2.7193	0.01		-0.19	*	-0.63	*	-0.79		-0.57		0.44		-0.44	*	0.60		0.38		0.16		-0.06		-0.22
222	Protein FAM153B	FAM153B	4.1749	0.0043	2.3634	0.01	*	1.05		0.59		0.46		0.65		0.46		-0.46		0.58		0.39		0.13		-0.06		-0.19
223	Protein hinderin	KIAA1328	3.0891	0.0211	1.6750	0.04	*	0.92		0.50		0.81		0.74		0.42		-0.42		0.11		0.18		-0.31		-0.24		0.07
224	Protein MMS22-like	MMS22L	9.3706	0.0000	5.3976	0.00	*	-0.90	*	-1.50	*	-1.11		-0.64		0.60		-0.60		0.21		-0.26		-0.39	*	-0.86		-0.47
225	Protein phosphatase 1 regulatory subunit 12C	PPP1R12C	2.9914	0.0244	1.6127	0.05		0.27		-1.55	*	-1.76		-1.53		1.82		-1.82		2.02		1.80		0.20		-0.02		-0.23
226	Protein phosphatase 1D	PPM1D	3.8855	0.0066	2.1810	0.02		-0.16	*	-0.45	*	-0.50		-0.38		0.29		-0.29		0.35		0.23		0.05		-0.07		-0.12
227	Protein piccolo	PCLO	4.1776	0.0043	2.3650	0.01		-0.12		-0.25	*	-0.55		-0.25		0.13		-0.13	*	0.43		0.13		0.30		0.00		-0.30
228	Protein S100-A9	S100A9	3.3328	0.0148	1.8303	0.03		3.10		-0.59		1.50		0.58	*	3.69	*	-3.69		1.60		2.52		-2.09		-1.17		0.92
229	Protein shortage in chiasmata 1 ortholog	SHOC1	3.9463	0.0060	2.2194	0.02		-0.42		-0.47		0.04		-0.49		0.05		-0.05		-0.46		0.07		-0.50		0.02		0.53
230	Protein-arginine deiminase type-6	PADI6	5.5189	0.0006	3.1939	0.00	*	3.32	*	2.33	*	2.99	*	1.66		0.99		-0.99		0.33		1.67		-0.66		0.68		1.34
231	Prothrombin	F2	8.7796	0.0000	5.0768	0.00	*	-0.58	*	-0.89	*	-0.74	*	-0.58		0.31		-0.31		0.16		0.00		-0.15		-0.31		-0.16
232	PTB-containing_ cubilin and LRP1-interacting protein	PID1	6.4201	0.0002	3.7336	0.00	*	-0.92	*	-1.50	*	-1.42	*	-1.21		0.58		-0.58		0.49		0.29		-0.09		-0.29		-0.20
233	Putative DENN domain-containing protein 10 B	DENND10P1	8.6891	0.0000	5.0271	0.00	*	1.43	*	1.73	*	2.22	*	1.39		-0.31		0.31		-0.79		0.03		-0.48		0.34		0.82
234	Regulator of G-protein signaling 10	RGS10	16.9500	0.0000	9.0348	0.00	*	0.91	*	2.01	*	1.86	*	0.80	*	-1.10	*	1.10	*	-0.94		0.11		0.16	*	1.21	*	1.05
235	Retinol-binding protein 4	RBP4	10.3050	0.0000	5.8925	0.00	*	-1.06	*	-0.84	*	-0.91	*	-0.83		-0.22		0.22		-0.16		-0.23		0.06		-0.01		-0.07
236	Rho guanine nucleotide exchange factor 17	ARHGEF17	6.9045	0.0001	4.0176	0.00	*	1.05	*	2.10	*	1.38		1.43		-1.06		1.06		-0.33		-0.38		0.72		0.67		-0.05
237	S-adenosylmethionine decarboxylase proenzyme	AMD1	17.0560	0.0000	9.0800	0.00	*	1.69	*	1.34	*	1.56	*	1.64		0.35		-0.35		0.14		0.05		-0.21		-0.30		-0.09
238	Schlafen-like protein 1	SLFNL1	4.2515	0.0039	2.4114	0.01		1.49	*	2.24		1.63		1.04		-0.75		0.75		-0.14		0.45		0.60		1.20		0.59
239	Sciellin	SCEL	3.0590	0.0221	1.6558	0.05		0.02	*	-0.55		-0.27		-0.10		0.57		-0.57		0.29		0.13		-0.28		-0.44		-0.17
240	Secernin-2	SCRN2	5.5681	0.0006	3.2237	0.00		1.51	*	2.70	*	3.45		0.78		-1.18		1.18		-1.94		0.73		-0.75		1.92	*	2.67
241	Septin-6	SEPTIN6	3.9025	0.0064	2.1917	0.02	*	0.60		0.04		0.35		0.41	*	0.56	*	-0.56		0.25		0.19		-0.31		-0.37		-0.06
242	Serine/threonine-protein kinase MRCK beta	CDC42BPB	4.1867	0.0043	2.3707	0.01		-0.29		0.61		0.11		0.78	*	-0.90	*	0.90		-0.40	*	-1.07		0.50		-0.17		-0.68
243	Serine/threonine-protein kinase TAO3	TAOK3	9.9853	0.0000	5.7250	0.00	*	2.12	*	2.61	*	3.31	*	1.85		-0.49		0.49		-1.18		0.27		-0.69		0.76		1.46
244	Serine/threonine-protein kinase/endoribonuclease IRE1	ERN1	3.9777	0.0058	2.2392	0.02		0.64	*	2.01		1.27		0.78	*	-1.36	*	1.36		-0.63		-0.14		0.74		1.23		0.49
245	Serine-threonine kinase receptor-associated protein	STRAP	4.8804	0.0016	2.8029	0.01		-0.62		0.37		-0.06		-0.58	*	-0.99	*	0.99		-0.56		-0.04		0.43	*	0.95		0.52
246	Serum amyloid A-1 protein	SAA1	3.7730	0.0078	2.1099	0.02	*	1.70		-0.04		1.73		0.86	*	1.73	*	-1.73		-0.03		0.84		-1.76		-0.89		0.87
247	Serum amyloid A-2 protein	SAA2	3.5312	0.0111	1.9565	0.03		2.32		1.02	*	4.97		0.74		1.30		-1.30		-2.65		1.59		-3.95		0.28	*	4.23
248	Serum amyloid A-4 protein	SAA4	6.5150	0.0002	3.7896	0.00		0.11	*	-0.55		0.12		0.00	*	0.66	*	-0.66		-0.01		0.12	*	-0.67	*	-0.55		0.12
249	Serum amyloid P-component	APCS	4.9124	0.0015	2.8227	0.01		-0.17	*	-0.68	*	-0.51		-0.45	*	0.51	*	-0.51		0.35		0.28		-0.17		-0.24		-0.07
250	Serum paraoxonase/arylesterase 1	PON1	8.3206	0.0000	4.8233	0.00	*	-1.18	*	-1.07	*	-1.12		-0.65		-0.11		0.11		-0.05		-0.53		0.06		-0.42		-0.48
251	SH2 domain-containing protein 6	SH2D6	4.6152	0.0023	2.6386	0.01		0.04	*	0.91		0.44		-0.19	*	-0.87	*	0.87		-0.40		0.22		0.47	*	1.10		0.63
252	Sialic acid-binding Ig-like lectin 9	SIGLEC9	7.1227	0.0001	4.1441	0.00	*	-1.22	*	-0.90		-0.70		-0.50		-0.32		0.32		-0.52	*	-0.72		-0.20		-0.41		-0.21
253	Sorting nexin-4	SNX4	6.5538	0.0002	3.8124	0.00	*	-0.95	*	-1.12	*	-0.88		-0.30		0.17		-0.17		-0.07		-0.65		-0.24	*	-0.81		-0.58
254	Spectrin beta chain_ non-erythrocytic 2	SPTBN2	6.5456	0.0002	3.8076	0.00		0.35	*	0.81		0.49		0.12		-0.46		0.46		-0.14		0.23		0.32	*	0.69		0.37
255	Spectrin beta chain_ non-erythrocytic 5	SPTBN5	5.7074	0.0005	3.3080	0.00	*	-1.31	*	-1.24	*	-0.98		-0.82		-0.08		0.08		-0.34		-0.49		-0.26		-0.42		-0.16
256	Spermidine synthase	SRM	5.1293	0.0011	2.9561	0.00	*	-0.76		-0.16	*	-0.79		-0.05		-0.60		0.60		0.04		-0.71		0.64		-0.11	*	-0.75
257	Succinate dehydrogenase [ubiquinone] iron-sulfur subunit_ mitochondrial	SDHB	12.0310	0.0000	6.7697	0.00	*	1.06	*	1.63	*	1.30	*	1.06		-0.58		0.58		-0.24		-0.01		0.33		0.57		0.23
258	Superoxide dismutase [Mn]_ mitochondrial	SOD2	10.8180	0.0000	6.1583	0.00	*	4.68	*	5.77	*	4.93	*	4.08		-1.09		1.09		-0.25		0.60		0.84		1.69		0.85
259	T cell receptor beta variable 24-1	TRBV24-1	3.6837	0.0088	2.0533	0.02		-0.39		0.53		0.04		-0.44	*	-0.93	*	0.93		-0.43		0.05		0.50	*	0.98		0.48
260	T-complex protein 11 X-linked protein 1	TCP11X1	3.5087	0.0114	1.9422	0.03	*	-0.90		-0.92		-0.94	*	-1.25		0.02		-0.02		0.03		0.35		0.01		0.33		0.32
261	Tenascin-X	TNXB	8.8925	0.0000	5.1385	0.00	*	1.89	*	1.93	*	1.98		0.85		-0.04		0.04		-0.09		1.04		-0.05		1.08		1.13
262	Thioredoxin domain-containing protein 15	TXNDC15	13.7080	0.0000	7.5787	0.00	*	2.09	*	1.58	*	2.19	*	1.29		0.50		-0.50		-0.11		0.80		-0.61		0.29		0.90
263	Thioredoxin-like protein 1	TXNL1	7.2969	0.0001	4.2445	0.00		-0.58	*	-1.08	*	-0.84		-0.51		0.50		-0.50		0.27		-0.07		-0.23	*	-0.57		-0.34
264	Thrombospondin-1	THBS1	8.5202	0.0000	4.9340	0.00	*	-0.47	*	-0.78	*	-0.62	*	-0.50		0.32		-0.32		0.16		0.03		-0.16		-0.28		-0.13
265	Transcription cofactor vestigial-like protein 1	VGLL1	15.2770	0.0000	8.3004	0.00	*	-3.16	*	-3.09	*	-2.90	*	-2.56		-0.07		0.07		-0.26		-0.60		-0.19		-0.52		-0.34
266	Transcription factor ETV7	ETV7	9.2616	0.0000	5.3389	0.00	*	-1.12	*	-0.75		-0.42		-0.40		-0.37		0.37	*	-0.70	*	-0.72		-0.33		-0.35		-0.02
267	Transcriptional adapter 2-alpha	TADA2A	4.8355	0.0017	2.7752	0.01	*	-0.85		-0.11	*	-0.64		-0.32	*	-0.74	*	0.74		-0.21		-0.53		0.53		0.22		-0.32
268	Transthyretin	TTR	14.4510	0.0000	7.9248	0.00	*	-1.49	*	-1.37	*	-1.37	*	-1.11		-0.12		0.12		-0.11		-0.37		0.00		-0.26		-0.26
269	Trinucleotide repeat-containing gene 18 protein	TNRC18	3.7551	0.0080	2.0985	0.02		0.48		-0.43		-0.54		0.37		0.90		-0.90	*	1.01		0.10		0.11		-0.80		-0.91
270	Trinucleotide repeat-containing gene 6A protein	TNRC6A	31.1230	0.0000	14.1640	0.00	*	3.63	*	4.86	*	4.37	*	3.69	*	-1.23	*	1.23		-0.74		-0.06		0.48		1.16		0.68
271	tRNA N(3)-methylcytidine methyltransferase METTL6	METTL6	3.0399	0.0227	1.6436	0.05		0.46		-0.46		0.23		0.03	*	0.92	*	-0.92		0.23		0.43		-0.69		-0.49		0.20
272	TSC22 domain family protein 3	TSC22D3	14.9530	0.0000	8.1537	0.00	*	1.72	*	1.15	*	1.26	*	1.59		0.57		-0.57		0.46		0.13		-0.11		-0.44		-0.33
273	t-SNARE domain-containing protein 1	TSNARE1	10.2360	0.0000	5.8569	0.00	*	-0.98	*	-1.02	*	-0.84	*	-0.76		0.04		-0.04		-0.14		-0.22		-0.18		-0.26		-0.08
274	Twinkle mtDNA helicase	TWNK	10.3720	0.0000	5.9277	0.00	*	3.62	*	5.49	*	5.04	*	3.22		-1.87		1.87		-1.42		0.39		0.45		2.26		1.82
275	Tyrosine-protein kinase Tec	TEC	10.4610	0.0000	5.9742	0.00	*	3.76	*	4.59	*	4.24	*	3.08		-0.83		0.83		-0.48		0.68		0.34		1.50		1.16
276	Ubiquitin D	UBD	3.1302	0.0199	1.7013	0.04		0.25		-0.34		-0.19		0.09		0.58		-0.58		0.44		0.16		-0.14		-0.42		-0.28
277	Ubiquitin domain-containing protein 1	UBTD1	7.9416	0.0000	4.6113	0.00		-1.50	*	-2.66	*	-2.15		-1.15		1.16		-1.16		0.65		-0.35		-0.51	*	-1.51		-1.00
278	Ubl carboxyl-terminal hydrolase 18	USP18	5.5240	0.0006	3.1970	0.00	*	-0.91		-0.42	*	-0.63	*	-0.67		-0.49		0.49		-0.28		-0.24		0.21		0.25		0.04
279	UL16-binding protein 1	ULBP1	45.6650	0.0000	18.0900	0.00	*	4.21	*	4.70	*	4.83	*	4.15		-0.49		0.49		-0.62		0.06		-0.12		0.55		0.67
280	Uncharacterized protein C3orf20	C3orf20	5.0084	0.0013	2.8819	0.00	*	-0.62	*	-0.88	*	-0.74	*	-0.89		0.26		-0.26		0.12		0.27		-0.14		0.01		0.15
281	Uncharacterized protein C9orf153	C9orf153	3.2639	0.0164	1.7865	0.04	*	1.23		0.98		1.10		0.12		0.24		-0.24		0.13		1.11		-0.11		0.86		0.98
282	Unconventional myosin-XV	MYO15A	5.9788	0.0003	3.4712	0.00	*	-0.88	*	-0.69		-0.32		-0.27		-0.19		0.19	*	-0.56	*	-0.61		-0.37		-0.42		-0.05
283	Vacuolar protein sorting-associated protein 13D	VPS13D	5.9811	0.0003	3.4725	0.00	*	-0.47	*	-0.57	*	-0.64	*	-0.53		0.10		-0.10		0.18		0.06		0.08		-0.04		-0.12
284	Vascular cell adhesion protein 1	VCAM1	24.0530	0.0000	11.8170	0.00	*	1.24	*	2.06	*	1.94	*	1.05	*	-0.82	*	0.82		-0.70		0.19		0.12	*	1.01	*	0.89
285	Vasorin	VASN	6.0699	0.0003	3.5256	0.00		0.12	*	1.85	*	0.80		0.53	*	-1.73	*	1.73		-0.68		-0.41		1.05		1.31		0.26
286	Villin-like protein	VILL	6.4553	0.0002	3.7544	0.00	*	-0.96	*	-0.70		-0.33		-0.30		-0.26		0.26	*	-0.63	*	-0.66		-0.37		-0.39		-0.03
287	Vitamin D-binding protein	GC	4.6710	0.0021	2.6732	0.01		-0.48	*	-0.56	*	-0.64	*	-0.65		0.08		-0.08		0.16		0.17		0.08		0.09		0.01
288	Vitamin K-dependent protein C	PROC	10.9900	0.0000	6.2466	0.00	*	-1.24	*	-2.39	*	-1.93		-0.98		1.14		-1.14		0.69		-0.26		-0.45	*	-1.41	*	-0.95
289	Vitronectin	VTN	4.4209	0.0030	2.5174	0.01		-0.46	*	-0.71	*	-0.67		-0.41		0.24		-0.24		0.20		-0.06		-0.04		-0.30		-0.26
290	Voltage-dependent calcium channel subunit alpha-2/delta-3	CACNA2D3	3.6863	0.0088	2.0549	0.02		0.11		-0.31	*	-0.46		-0.32		0.42		-0.42	*	0.57		0.43		0.15		0.00		-0.14
291	von Willebrand factor	VWF	13.8900	0.0000	7.6643	0.00	*	1.90	*	2.34	*	2.26	*	1.13		-0.44		0.44		-0.36		0.77		0.09	*	1.22	*	1.13
292	WD repeat-containing protein 31	WDR31	5.7861	0.0004	3.3554	0.00	*	-0.88		0.14		-0.28	*	-0.81	*	-1.02	*	1.02		-0.60		-0.07		0.42		0.95		0.53
293	WD repeat-containing protein 53	WDR53	4.1400	0.0046	2.3414	0.01	*	-1.46		-1.13		-0.71		-0.66		-0.33		0.33		-0.76		-0.80		-0.43		-0.47		-0.05
294	WD repeat-containing protein 87	WDR87	4.1695	0.0044	2.3599	0.01		-0.32		-0.37		0.08		-0.26		0.05		-0.05		-0.40		-0.06	*	-0.45		-0.11		0.34
295	Zinc finger CCCH domain-containing protein 15	ZC3H15	4.3525	0.0034	2.4747	0.01		0.69	*	1.26		0.99		0.29		-0.58		0.58		-0.30		0.40		0.27	*	0.98		0.70
296	Zinc finger protein 211	ZNF211	4.1175	0.0047	2.3273	0.01	*	-1.10		-0.70		-0.77		-0.73		-0.41		0.41		-0.34		-0.38		0.07		0.03		-0.04
297	Zinc finger protein 300	ZNF300	4.8378	0.0017	2.7766	0.01		0.42	*	0.95		0.58		0.25		-0.53		0.53		-0.16		0.17		0.38	*	0.70		0.33
298	Zinc finger protein 532	ZNF532	3.9114	0.0063	2.1974	0.02	*	-2.02		-1.03		-1.05		-0.89		-0.99		0.99		-0.97	*	-1.13		0.02		-0.14		-0.15
299	Zinc finger protein neuro-d4	DPF1	5.6802	0.0005	3.2916	0.00	*	-0.55	*	-0.62	*	-0.68		-0.36		0.07		-0.07		0.13		-0.19		0.06		-0.26		-0.31
300	Zygote arrest protein 1	ZAR1	11.5510	0.0000	6.5307	0.00	*	-1.30	*	-0.80	*	-0.72	*	-0.74		-0.49		0.49	*	-0.58		-0.55		-0.09		-0.06		0.03
