# Supplementary material for: Unveiling novel serum biomarkers in intrahepatic cholangiocarcinoma: a pilot proteomic exploration
Source: Front Pharmacol. 2024 Sep 2;15:1440985. doi: 10.3389/fphar.2024.1440985 (PMC11403330; doi:10.3389/fphar.2024.1440985)
Supplement: Supplementary file 4 [file DataSheet1.pdf]

Supplementary methods. LC-MS^E and search parameters

Description of experiment settings for LC-MS^E analysis and identification of proteins

**LC-Parameters**	
LC gradient<br>A: 0.1 % formic acid in water<br>B: 0.1 % formic acid in acetonitrile	0min-5% B-5m-5%-95m-40%-100m-85%-105m-85%-106m-5%-120m-5%
Flow rate	0.300µL/min
Column temperature	55°C
**MS^E-Parameters**	
Lock spray	GluFib precursor 785.8426 m/z
Acquisition times	10-110min
Acquisition mode	positive, resolution
Mass range	50-2000Da
Capillary voltage	3kV
Sampling cone	40V
Source offset	80V
Source temperature	80°C
Nebuliser gas flow	6.5 bar
Trap collision energy	4V
Transfer collision energy	2V
**Protein search parameter**	
Name of peak list-generating software and release version (number or date)	Progenesis QI v3.0 (Waters Corporation)
Name of the search engine and release version (number or date)	MSE built in search engine of Progenesis QI v2.0 (Waters Corporation)
Name of database searched and release version/date	Swissprot database release 2022/03 limited to human entries
Enzyme specificity considered	Fully tryptic
# of missed cleavages permitted	1
Fixed modification(s) (including residue specificity)	carbamidomethylation of cysteine
Variable modification(s) (including residue specificity)	oxidation of methionine
Fragment ions per protein	5
Fragment ions per peptide	2
Threshold score for accepting protein identification	≥1 significant peptides
Threshold score/E-value for accepting individual MS/MS Spectra	False discovery rate at peptide level <4%
software/method used to evaluate site assignment	No PTM reported
